# Probing the Histamine H_1_ Receptor Binding Site to Explore Ligand Binding Kinetics

**DOI:** 10.1021/acs.jmedchem.4c02043

**Published:** 2024-12-26

**Authors:** Sebastiaan Kuhne, Reggie Bosma, Albert J. Kooistra, Rick Riemens, Marc C. M. Stroet, Henry F. Vischer, Chris de Graaf, Maikel Wijtmans, Rob Leurs, Iwan J. P. de Esch

**Affiliations:** Amsterdam Institute of Molecular and Life Sciences (AIMMS), Division of Medicinal Chemistry, Faculty of Science, Vrije Universiteit Amsterdam, De Boelelaan 1108, 1081 HZ Amsterdam, The Netherlands

## Abstract

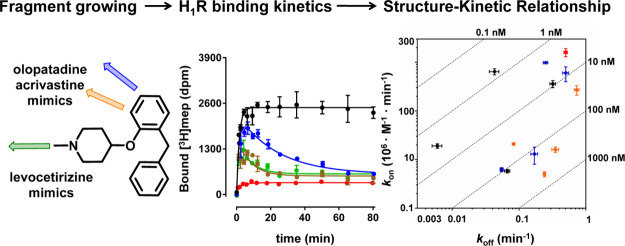

Analysis of structure-kinetic relationships (SKR) can contribute to an improved understanding of receptor–ligand interactions. Here, fragment **1** (4-(2-benzylphenoxy)-1-methylpiperidine) was used in different fragment growing approaches to mimic the putative binding mode of the long residence time (RT) ligands olopatadine, acrivastine, and levocetirizine at the histamine H_1_ receptor (H_1_R). SKR analyses reveal that introduction of a carboxylic acid moiety can increase RT at H_1_R up to 11-fold. Ligand efficiency (LE) decreases upon the introduction of the negatively charged group, whereas kinetic efficiency (KE) increases up to 8.5-fold. The olopatadine/acrivastine mimics give up to 15-fold differences in the RT, while the levocetirizine mimics afford similar RTs with only a 3-fold difference. Therefore, the levocetirizine mimics are less sensitive to structural changes. This study illustrates that for H_1_R, there are several ways to increase RT but the different strategies differ significantly in SKR.

## Introduction

G protein-coupled receptors (GPCRs) modulate a wide range of cellular activities and form one of the largest protein classes in humans.^1^ These membrane-bound receptors play an important role in many disease areas and around 34% of the currently marketed drugs target GPCRs.^2,3^ In particular aminergic GPCRs have been extensively studied by structure–activity relationships (SAR) using ligand binding affinity and activity data. More recently, there has been a growing interest in studying ligand binding kinetics as they describe different aspects of the molecular recognition process.^4,5^ There is considerable debate on the impact that receptor–ligand binding kinetics has on the clinical efficacy of drug candidates,^6,7^ and studying binding kinetics early on in the drug discovery process has been postulated to provide a strategy to decrease the late-stage attrition rate.^8^ Receptor–ligand binding is characterized by the association rate constant (*k*_on_) and dissociation rate constant (*k*_off_), which together determine the equilibrium dissociation constant *K*_D_ (=*k*_off_/*k*_on_). A parameter that describes how long the ligand–receptor complex exists is the target residence time (RT) of a ligand, defined as the reciprocal of the *k*_off_ value. Structural and molecular determinants of binding affinity (*K*_D_) to aminergic GPCRs are well investigated, but fewer studies explore the molecular features that govern kinetic binding rate constants.

**Figure 1:**
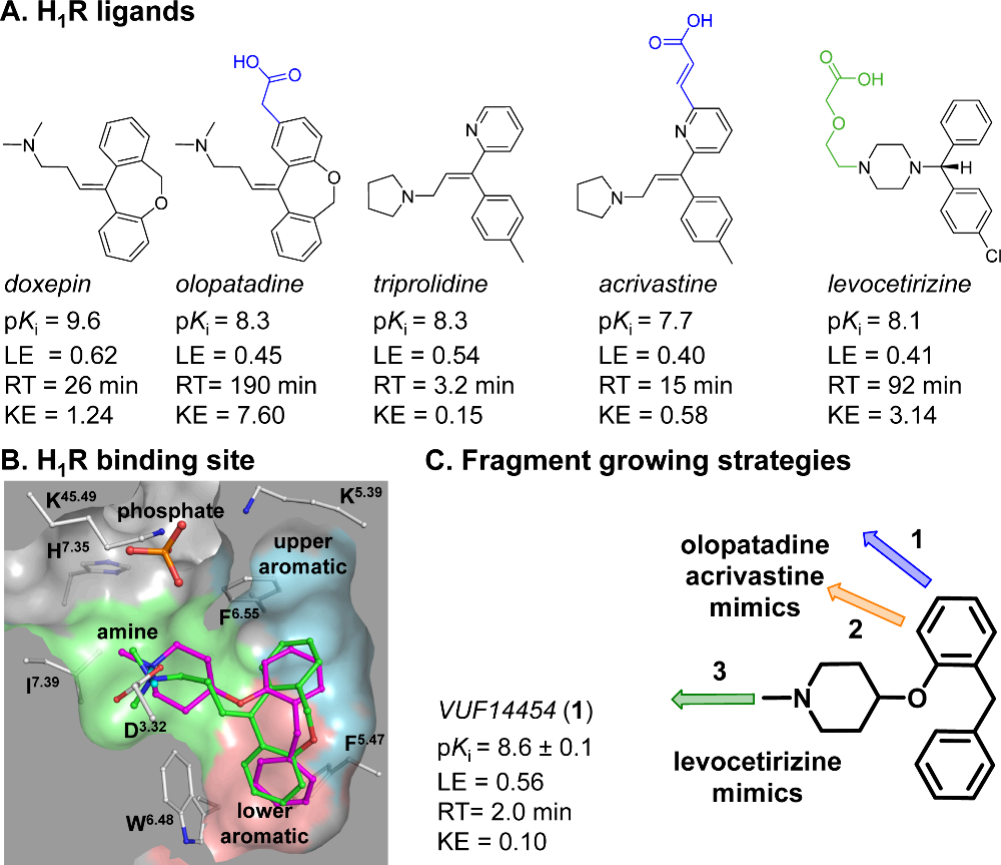
(A) Antihistamines targeting H_1_R with corresponding binding affinities (p*K*_i_) and RT, as determined in this study. Ligand efficiency (LE)^17^ = −*R*·*T*·ln(*K*_i_)/*N*, where *N* is the number of heavy atoms (HA, i.e., non-hydrogen atoms), and *R* = (8.31447215 J K^–1^ mol^–1^ and 4184 J = 1 kcal), and *T* = 298.15 K. Kinetic efficiency (KE)^20^ = RT/*N*, where RT is the residence time, and *N* is the number of HAs. (B) H_1_R X-ray structure (PDB-code: 3RZE) with the cocrystallized ligand doxepin (green carbon atoms) and the proposed binding mode of fragment **1** (magenta carbon atoms). Important binding site residues are represented as ball-and-sticks with light gray carbon atoms. Nitrogen, oxygen, and hydrogen atoms are colored blue, red, and cyan, respectively. Polar hydrogen atoms of the ligands are shown, but are absent for the binding site residues. H_1_R binding site surface is shown and colored to designate the four different regions of the binding site, i.e., the amine binding region, the lower aromatic binding region, the upper aromatic binding region, and the phosphate binding region. UniProt numbers (first) and the Ballesteros–Weinstein^21^ numbering (second in superscript) are reported for each residue throughout this manuscript. (C) Schematic 2D representation of fragment **1**, binding affinity and kinetic parameters (*k*_on_, *k*_off_, RT) as determined in this study, and the three different design strategies to probe the H_1_R SKR.

In this study, the structure–kinetic relationships (SKR) of a set of ligands binding to H_1_R are investigated. Historically, H_1_R ligands have been classified into two groups, i.e., the first- and second-generation (Figure 1A). First-generation ligands (e.g., doxepin and triprolidine) most often have two (fused) aromatic rings and a basic amine. Many second-generation ligands possess an additional carboxylic acid moiety, either attached to one of the aromatic rings (olopatadine and acrivastine) or to the basic amine (levocetirizine).^9^ Major advances in the structural understanding of ligand binding to H_1_R^10−12^ have been a welcome and essential contribution to the field, although such structures typically shed limited light on the kinetics of ligand binding. The first-generation ligand doxepin has been cocrystallized bound to the orthosteric binding site of H_1_R (Figure 1B).^11^ The structure reveals that doxepin binds between the transmembrane domains 3, 4, 5, and 6. The amine moiety of doxepin interacts with H_1_R residue D107^3.32^, a hallmark feature of all biogenic amine receptors. The aromatic moieties of the ligand are accommodated by two distinct hydrophobic pockets that are formed by (mostly) aromatic residues. In the doxepin-bound H_1_R crystal structure, a phosphate ion was also identified, binding between the orthosteric pocket and the extracellular vestibule, coordinated by the residues K191^5.39^, K179^45.49^, H435^7.35^, and Y431^6.51^ (Figure 1B). It has been suggested by molecular modeling and site-directed mutagenesis studies, that some of the second-generation antihistamines, e.g., levocetirizine, occupy this phosphate pocket with their anionic carboxylate group.^11,13−16^ The second-generation antihistamines olopatadine, acrivastine, and levocetirizine have decreased ligand efficiencies (LE, defined as the binding energy divided by the number of heavy atoms of a ligand^17^), but remarkably longer RTs^14,16,18^ and higher kinetic efficiencies (KE, defined as the RT divided by the number of heavy atoms of a ligand) than some of the first-generation ligands such as doxepin and triprolidine (Figure 1A). Therefore, targeting residues in the phosphate pocket with anionic moieties, such as carboxylic acids may represent a promising design strategy to increase RT for H_1_R receptor ligands.

We have previously identified fragment **1** as a high-affinity ligand for the human H_1_R. The fragment has been used to systematically probe different H_1_R binding regions, including the amine-binding region (D107^3.32^, W428^6.48^, Y431^6.51^, I454^7.39^, and Y458^7.43^), the upper (Y108^3.33^, W158^4.56^, Y431^6.51^, F432^6.52^, and F435^6.55^) and lower (F199^5.47^, F424^6.44^, and W428^6.48^) aromatic region, and was also used to explore the role of solvation in the H_1_R binding site on ligand binding interactions. In this study, well-defined binding hot spots were identified and it was shown that fragment **1** was targeting those hot spots optimally.^19^ The current study aims to build upon this molecular understanding of H_1_R–ligand interaction and to gain an understanding of H_1_R SKR. To this end, hit fragment **1** (RT = 2.0 ± 0.1 min) was used as a scaffold to design and synthesize a set of derivatives to explore H_1_R binding kinetics. Ligands interrogating distinct binding regions of H_1_R (Figure 1B,C) were designed and synthesized. Three different fragment growing and modification strategies were employed to modulate the binding kinetics by introducing carboxylic acid moieties at (1) the 5-position of the aromatic ring, (2) the 6-position of the aromatic ring, both mimicking olopatadine and acrivastine, and (3) the basic amine, mimicking levocetirizine (Figure 1C).

## Results

### Design and Synthesis of Tool Compounds to Investigate H_1_R SAR and SKR

**Figure 2:**
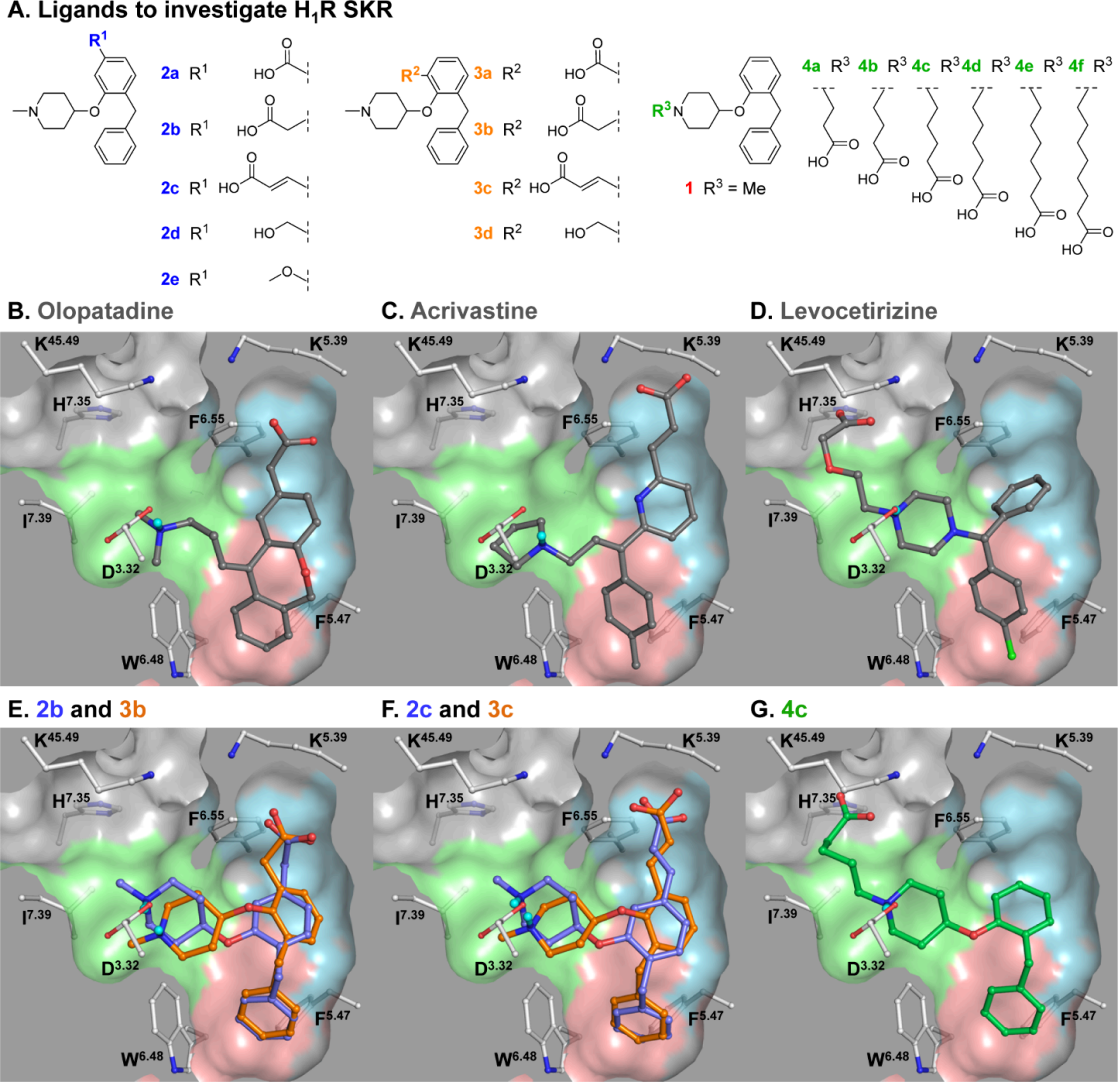
(A) Fragment growing of hit fragment **1**, mimicking the H_1_R reference compounds olopatadine, acrivastine, and levocetirizine, to study the H_1_R SKR. Proposed binding mode of the H_1_R reference compounds (B) olopatadine (gray carbon atoms), (C) acrivatine (gray carbon atoms), and (D) levocetirizine (gray carbon atoms) in H_1_R. Proposed binding mode of the (E) olopatadine mimics **2b** (blue carbon atoms) and **3b** (orange carbon atoms), (F) acrivastine mimics **2c** (blue carbon atoms) and **3c** (orange carbon atoms), and (G) levocetirizine mimics binding of **4c** (green carbon atoms) to H_1_R. Binding modes were obtained by docking the compounds in the H_1_R crystal structure (pdb: 3RZE).^11^ Rendering and color-coding are the same as in Figure 1.

To modulate H_1_R binding kinetics and hence investigate the SKR, we designed and synthesized a diverse set of 15 analogs of **1** (Figure 2A). Fragment **1** is believed to target the same aromatic regions and amine-binding region as doxepin in the H_1_R crystal structure, but not the anionic phosphate pocket. The designed ligands mimic the binding mode of the long-RT H_1_R reference compounds olopatadine, acrivastine, and levocetirizine as suggested by molecular modeling studies (Figure 2B–G).

#### Binding Mode Predictions

Binding modes of ligand **2b**, **3b**, **2c**, **3c**, **4c**, olopatadine, acrivastine, and levocetirizine were obtained using PLANTS docking in the doxepin-bound H_1_R crystal structure (PDB: 3RZE)^11^ (Figure 2B–G). As designed, the poses of ligand **2b** and **3b** have a similar positioning of the acetic acid moiety compared to the docking pose of olopatadine (Figure 2B,E), justifying growing from both the 5- and 6-postion of the aromatic ring of fragment **1**. The docking poses of **2c** and **3c** show a slight shift of the acrylic acid moiety compared to the docking pose of acrivastine (Figure 2C,F). The docking pose of **4c** shows that the carboxylic acid moiety can be positioned in a similar position as levocetirizine (Figure 2D,G), justifying growing from the basic nitrogen atom of fragment **1**. Furthermore, the docking pose of levocetirizine is in line with the binding mode described by Kooistra et al.^13^ Overlays of the binding modes between the antihistamines olopatadine, acrivastine, and levocetirizine together with **2b**, **3b**, **2c**, **3c**, and **4c**, that mimic these binding modes, are shown in Supporting Figure 1.

#### Probing the Olopatadine and Acrivastine Binding Region

**Scheme 1:**
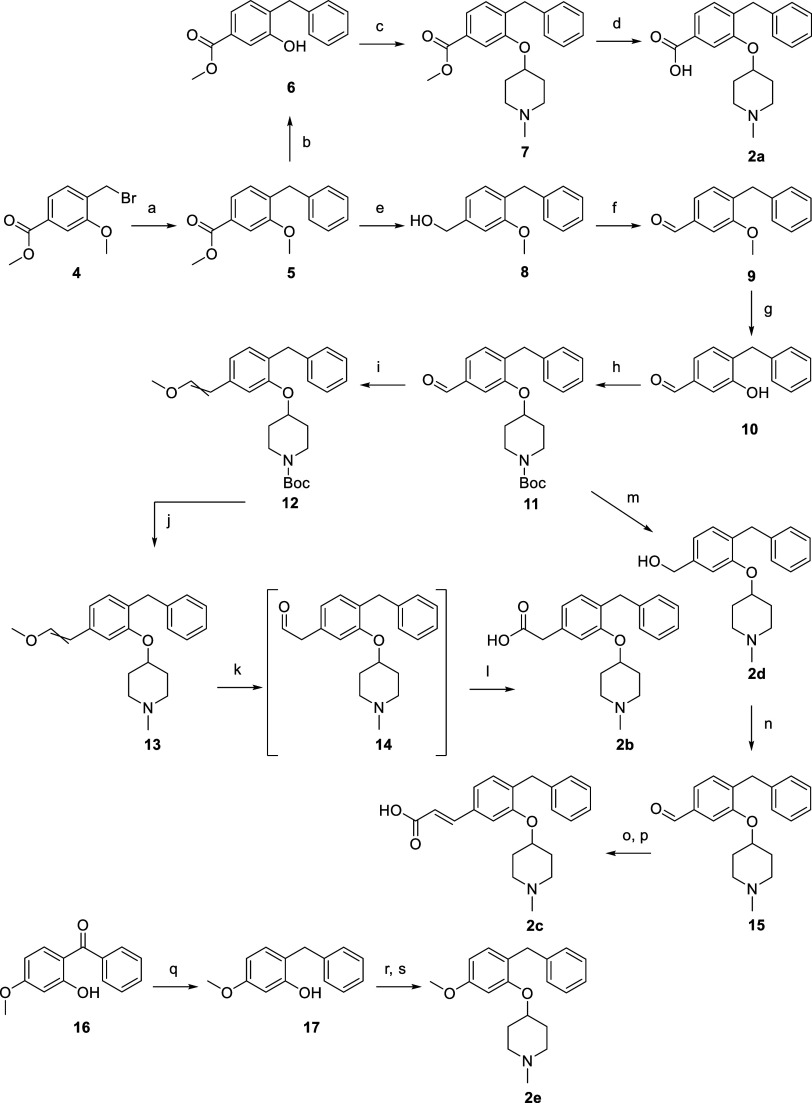
Synthesis of Ligands to Probe the Olopatadine and Acrivastine Binding Region: Growing from the 5-Position of the Aromatic Ring Reagents and conditions: (a) AlCl_3_, benzene, rt, 165 min, 73%. (b) BBr_3_, DCM, 0 °C to rt, 3 h, 70%. (c) 1-methylpiperidin-4-ol, PPh_3_, DEAD (40 wt % in toluene), THF, 0 °C to rt, 24 h, 31%. (d) 1.0 M NaOH (aq), MeOH, reflux, 5 h, 33%. (e) 1.0 M LAH in THF, THF, −78 °C to rt, 2.5 h, 96%. (f) MnO_2_, DCM, rt, 42 h, 93%. (g) EtSNa, DMF, 120 °C, 3 h, 61%. (h) *tert*-butyl 4-((methylsulfonyl)oxy)piperidine-1-carboxylate, Cs_2_CO_3_, DMF, 60 °C, 16 h, 74%. (i) *t*-BuOK, (methoxymethyl)triphenylphosphonium chloride, THF, rt, 6 h, 66%. (j) 1.0 M LAH in THF, THF, 0–45 °C, 8.5 h, 92%. (k) 1.0 M HCl in dioxane, H_2_O, rt, 1 h. (l) NaH_2_PO_4_·H_2_O, 2-methylbut-2-ene, NaClO_2_, *t*-BuOH, H_2_O, rt, 1 h, 6% over two steps. (m) 1.0 M LAH in THF, THF, 0 °C to reflux, 1.5 h, 38%. (n) MnO_2_, DCM, rt, 16 h. (o) Methyl (triphenylphosphoranylidene)acetate, toluene, 0 °C to rt, 48 h. (p) 2.0 M NaOH (aq), MeOH, reflux, 6 h, 7% over three steps. (q) Et_3_SiH, TFA, DCM, rt, 17 h, 52%. (r) *tert*-Butyl 4-((methylsulfonyl)oxy)piperidine-1-carboxylate, Cs_2_CO_3_, DMF, 65 °C, 22 h. (s) 1.0 M LAH in THF, THF, 0–50 °C, 4 h, 54% over two steps. Compound **2d** was converted to a hemifumaric acid salt and compound **2e** to a fumaric acid salt.

**Scheme 2:**
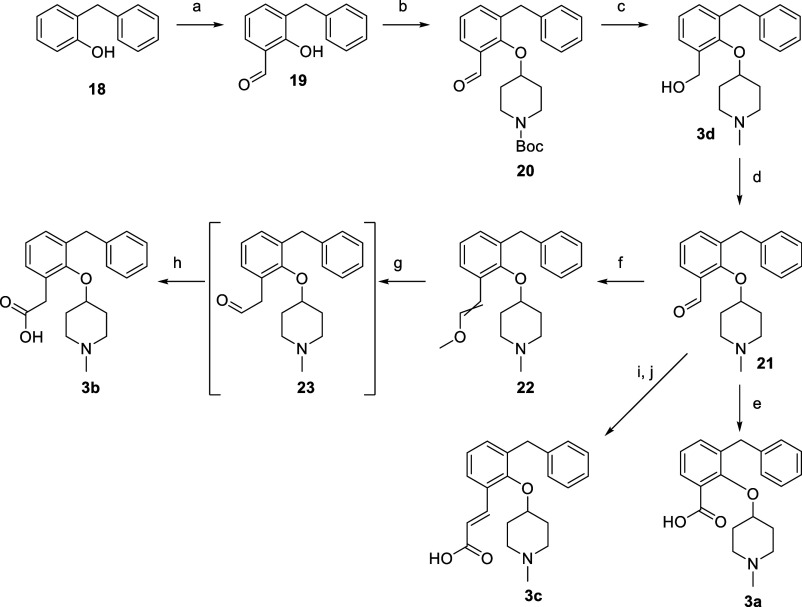
Synthesis of Ligands to Probe the Olopatadine and Acrivastine Binding Region: Growing from the 6-Position of the Aromatic Ring Reagents and conditions: (a) MgCl_2_, TEA, paraformaldehyde, THF, reflux, 4 h, 66%. (b) *tert*-butyl 4-((methylsulfonyl)oxy)piperidine-1-carboxylate, Cs_2_CO_3_, DMF, 60 °C, 32 h, 70%. (c) 1.0 M LAH in THF, THF, 0–60 °C, 4 h, 17%. (d) MnO_2_, DCM, rt, 42 h, 80%. (e) NaH_2_PO_4_·H_2_O, 2-methylbut-2-ene, NaClO_2_, *t*-BuOH, H_2_O, rt, 2 h, 2%. (f) *t*-BuOK, (methoxymethyl)triphenylphosphonium chloride, THF, rt, 16 h, 73%. (g) 1.0 M HCl in dioxane, H_2_O, rt, 1 h. (h) NaH_2_PO_4_.H_2_O, 2-methylbut-2-ene, NaClO_2_, *t*-BuOH, H_2_O, rt, 1 h, 9% over two steps. (i) Methyl (triphenylphosphoranylidene)acetate, THF, 0 °C to rt, 48 h. (j) 2.0 M NaOH (aq), MeOH, reflux, 6 h, 24% over two steps.

To mimic the binding mode of olopatadine and acrivastine, zwitterions **2b**/**3b** bearing an acetic acid moiety and **2c**/**3c** bearing an acrylic acid moiety (Figure 2A–C,E,F) were synthesized (Schemes 1 and 2). In addition, compounds with alternative spacer length (**2a**/**3a**) and small nonanionic polar groups (**2d**, **2e**, **3d**) (Figure 2A) were synthesized (Schemes 1 and 2). The compounds probing the olopatadine and acrivastine binding region, growing from the 5-position (**2a–e**), were synthesized as outlined in Scheme 1. Friedel-Craft alkylation of benzene by commercially available benzyl bromide **4** gave intermediate **5** (Scheme 1). Demethylation using BBr_3_ gave phenol **6** that was subsequently used in a Mitsunobu reaction to afford tertiary amine **7**. Hydrolysis of **7** gave zwitterion **2a**. Ester **5** was reduced with LAH to benzyl alcohol **8**, and subsequent oxidation with MnO_2_ yielded aldehyde **9**. Demethylation using sodium ethanethiolate afforded phenol **10**, and subsequent reaction with *tert*-butyl 4-((methylsulfonyl)oxy)piperidine-1-carboxylate^19^ yielded aldehyde **11**. A Wittig reaction of aldehyde **11** with (methoxymethyl)triphenylphosphonium chloride gave after column chromatography an inseparable mixture of *E*/*Z* isomers of enol ether **12**. Subsequent reduction of the Boc group in **12** with LAH gave tertiary amine **13** as an inseparable mixture of *E*/*Z* isomers. Enol ether deprotection using HCl gave aldehyde **14**, which owing to the presence of the potentially unstable aryl-acetaldehyde moiety, was immediately subjected to an in situ Pinnick oxidation to afford after reversed-phase column chromatography zwitterion **2b**. Aldehyde **11** was also used to synthesize benzyl alcohol **2d** applying LAH to reduce both the aldehyde and Boc-group to an alcohol and methyl group, respectively. Oxidation of benzyl alcohol **2d** with MnO_2_ gave crude aldehyde **15**, which was subjected to a tandem Wittig reaction and saponification reaction to afford zwitterion **2c**. Treatment of ketone **16** with Et_3_SiH and TFA gave phenol **17**. Coupling with *tert*-butyl 4-((methylsulfonyl)oxy)piperidine-1-carboxylate^19^ and subsequent reduction of the Boc group afforded MeO-derivative **2e**.

The compounds probing the olopatadine and acrivastine binding region, growing from the 6-position (**3a–d**), were synthesized as outlined in Scheme 2. *ortho*-Formylation^22,23^ of commercially available phenol **18** using paraformaldehyde, MgCl_2_ and TEA gave aldehyde **19** (Scheme 2). Compound **19** was reacted with *tert*-butyl 4-((methylsulfonyl)oxy)piperidine-1-carboxylate^19^ to afford aldehyde **20**. The aldehyde and Boc group in compound **20** were reduced with LAH to an alcohol and methyl group, respectively, to give benzyl alcohol **3d**. Oxidation of **3d** with MnO_2_ yielded aldehyde **21**, a versatile building block. It was used in a Pinnick oxidation to give zwitterion **3a** (albeit in very low isolated yield) and in a Wittig reaction with (methoxymethyl)triphenylphosphonium chloride to give enol ether **22** as an inseparable mixture of *E*/*Z* isomers after column chromatography. The enol ether mixture was deprotected with HCl to give aldehyde **23** and subsequent in situ Pinnick oxidation furnished after reversed-phase chromatography zwitterion **3b**. Aldehyde **21** was also used for a Wittig reaction with methyl (triphenylphosphoranylidene)acetate and a subsequent hydrolysis reaction to obtain zwitterion **3c**.

#### Probing the Levocetirizine Binding Region

**Scheme 3:**
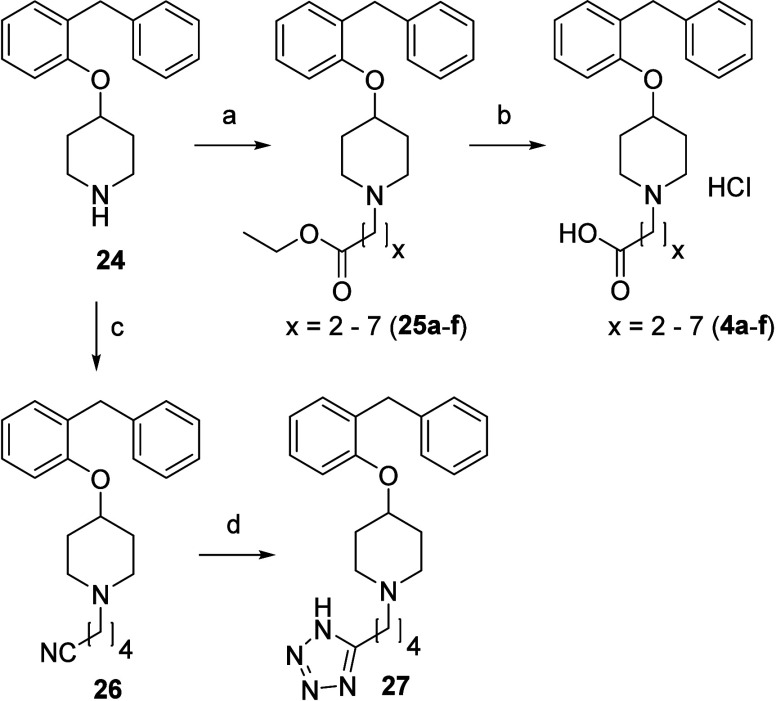
Synthesis of Ligands to Probe the Levocetirizine Binding Region: Growing from the Basic Nitrogen Atom Reagents and conditions: (a) corresponding bromo-ester, DMF, K_2_CO_3_, 80 °C, 3–9 h, 32–71%. (b) [1] 2.0 M NaOH (aq), MeOH, reflux, 3 h. [2] 1.0 M HCl (aq), rt, 30 min, 30–50%. (c) 5-Bromopentanenitrile, K_2_CO_3_, DMF, 85 °C, 2 h, 28% as fumarate salt. (d) NaN_3_, NH_4_Cl, DMF, 100 °C, 72 h, 28%.

To mimic the binding mode of levocetirizine, zwitterions **4a–f** with an increasing number of methylene units (Figure 2A,D,G) were synthesized (Scheme 3). Compound **24**(19) was alkylated on the amine with the corresponding bromo-esters to afford intermediates **25a–f**. Subsequent ester hydrolysis yielded the desired zwitterions **4a–f**, which were isolated as the HCl salts. Compound **24** was also alkylated using 5-bromopentanenitrile to afford nitrile **26**, which was treated with NaN_3_ and NH_4_Cl to obtain tetrazole **27**.

### Pharmacological Evaluation

**Figure 3:**
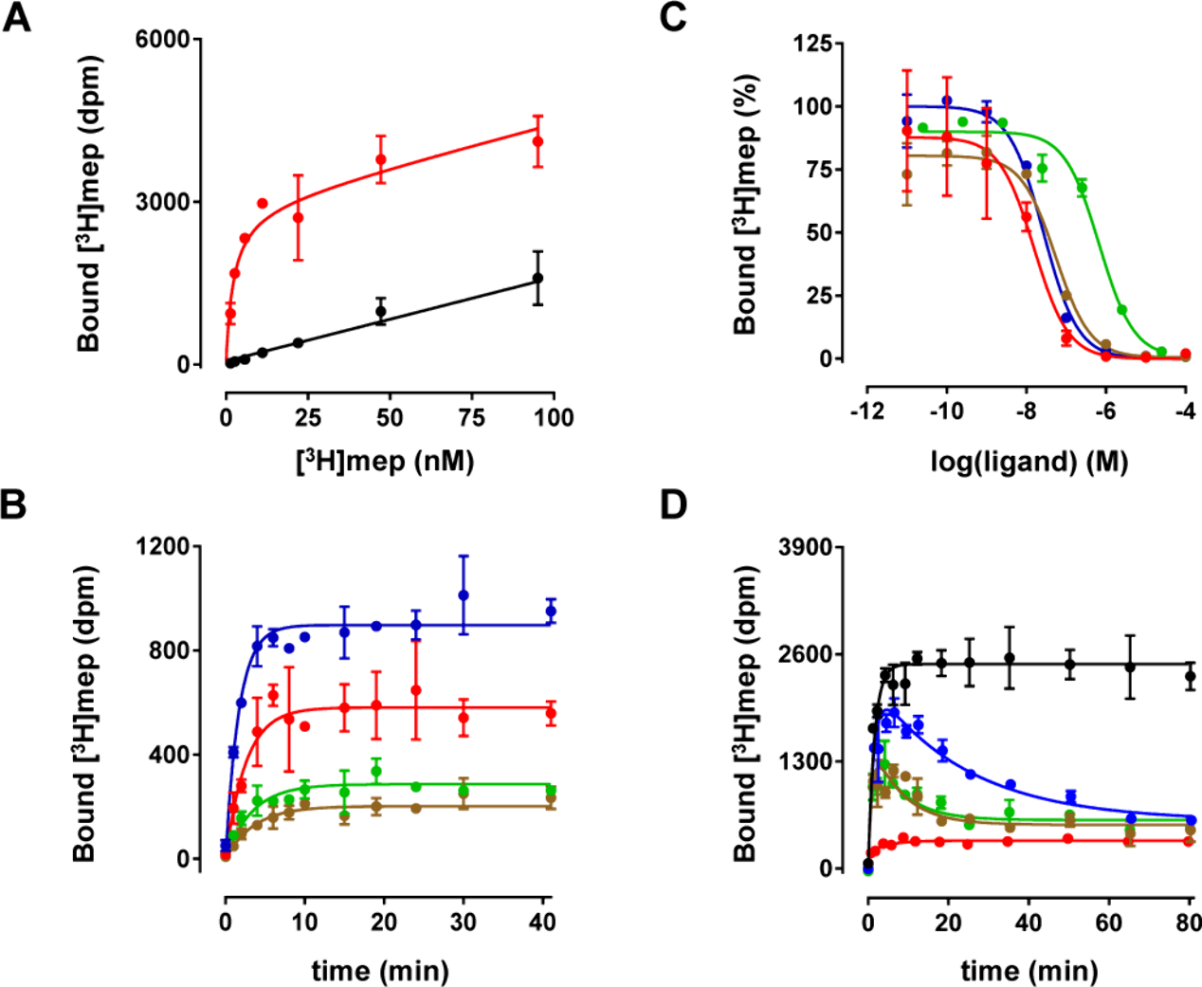
Characterization of ligand binding to a homogenate of HEK293T cells expressing H_1_R using [^3^H]-mepyramine. (A) HEK293T cell homogenate expressing H_1_R was incubated with increasing concentrations of [^3^H]-mepyramine for 4 h in the absence (red) or presence (black) of 10^–5^ M mianserin. (B) Cell homogenate was incubated with 2.9 nM (blue), 1.3 nM (red), 0.5 nM (green), and 0.3 nM (brown) of [^3^H]-mepyramine for various incubation times. (C) Cell homogenate was incubated with a single concentration [^3^H]-mepyramine with increasing concentrations of unlabeled ligand for 4 h. Ligands are depicted by the red curve (**1**), green curve (**2c**), brown curve (**4c**) and blue curve (levocetirizine). (D) Single concentration [^3^H]-mepyramine was coincubated for various times with cell homogenate in the absence (black) or presence of an approximate concentration of 10 times the respective *K*_i_ of these unlabeled ligands (red curve (**1**), green curve (**2c**), brown curve (**4c**) and blue curve (levocetirizine)). Representative graphs are shown of *N* ≥ 3 experiments with mean and SD of triplicate values (A, C) or duplicate values (B, D).

Equilibrium dissociation constants (*K*_i_) of the compounds (Tables 1 and 2) for human H_1_R were determined using [^3^H]-mepyramine radioligand-binding studies on HEK293T cell homogenates that transiently express H_1_R. The affinity of [^3^H]-mepyramine for H_1_R was determined by incubating increasing concentrations of radioligand with cell homogenate in the absence or presence of 10^–5^ M mianserin, reflecting the total (red) and nonspecific binding (black) of radioligand, respectively (Figure 3A). The affinity (p*K*_D_) and *B*_max_ values of specific [^3^H]-mepyramine binding were determined to be 8.6 ± 0.1 and 72 ± 12 pmol/mg protein. Kinetic binding rate constants were determined by incubating four different concentrations of [^3^H]-mepyramine with cell homogenate for increasing incubation times (Figure 3B). From the observed association binding the kinetic binding rate constants were determined for [^3^H]-mepyramine (*k*_on_: 110 ± 6 × 10^6^ M^–1^·min^–1^; *k*_off_: 0.22 ± 0.01 min^–1^). The calculated affinity of [^3^H]-mepyramine from its binding rate constants (p*K*_D,calc_) was 8.7 ± 0.0, which agrees well with the affinity determined in saturation binding experiments. Competition binding experiments were performed by incubating ±3 nM [^3^H]-mepyramine with increasing concentration of unlabeled ligand for 4 h and is depicted for a set of representative ligands in Figure 3C. Concentrations that displaced half-maximal binding of [^3^H]-mepyramine were used to calculate the binding affinity (p*K*_i_) of the unlabeled ligands using the Cheng–Prusoff equation^24^ (Tables 1 and 2). Finally, association binding of ±3 nM [^3^H]-mepyramine was measured in competition with a concentration of ±10 times the *K*_i_ of unlabeled test ligand (Figure 3D). For the unlabeled ligands levocetirizine (blue), **2c** (green) and **4c** (brown), but not for **1** (red), an overshoot in [^3^H]-mepyramine binding for early time points is clearly observed. This overshoot reflects the relatively low *k*_off_ of levocetirizine, **2c** and **4c** compared to [^3^H]-mepyramine, explaining the observed kinetic advantage of the radioligand for binding to H_1_R. Analysis of these curves using the Motulsky and Mahan model^25^ indeed showed that levocetirizine has the lowest dissociation rate constant (0.011 ± 0.001 min^–1^), followed by **4c** (0.045 ± 0.004 min^–1^) and **3c** (0.055 ± 0.003 min^–1^) and a high kinetic dissociation rate constant of **1** (0.55 ± 0.03 min^–1^). Kinetic binding rate constants and binding affinities for these and other unlabeled ligands are shown in Tables 1 and 2.

**Table 1:**
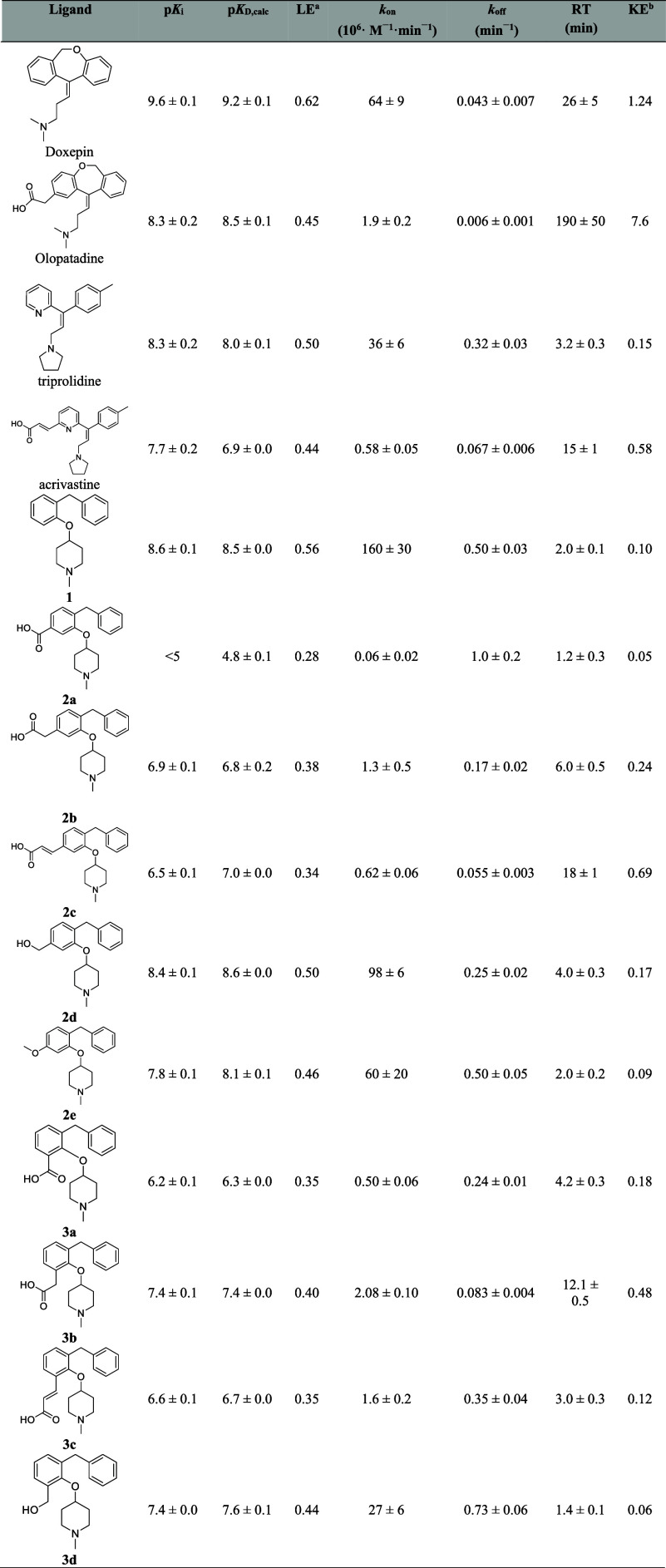
Pharmacological Evaluation of H_1_R Reference Compounds Olopatadine, Doxepin, Acrivastine, Triprolidine, and Ligands Probing the Olopatadine and Acrivastine Binding Region^a^

a Data were obtained from competition binding experiments to H_1_R using [^3^H]mepyramine as radioligand. *k*_on_ and *k*_off_ were obtained from the competitive binding of the radioligand over time, and p*K*_D_ and RT values were calculated for individual experiments from these binding rate constants. p*K*_i_ values were obtained from radioligand displacement curves after 4 h incubation with the radioligand. Data represent the mean and SEM of *N* ≥ 3.

b LE calculated as described in Figure 1.

c KE calculated as described in Figure 1.

**Table 2:**
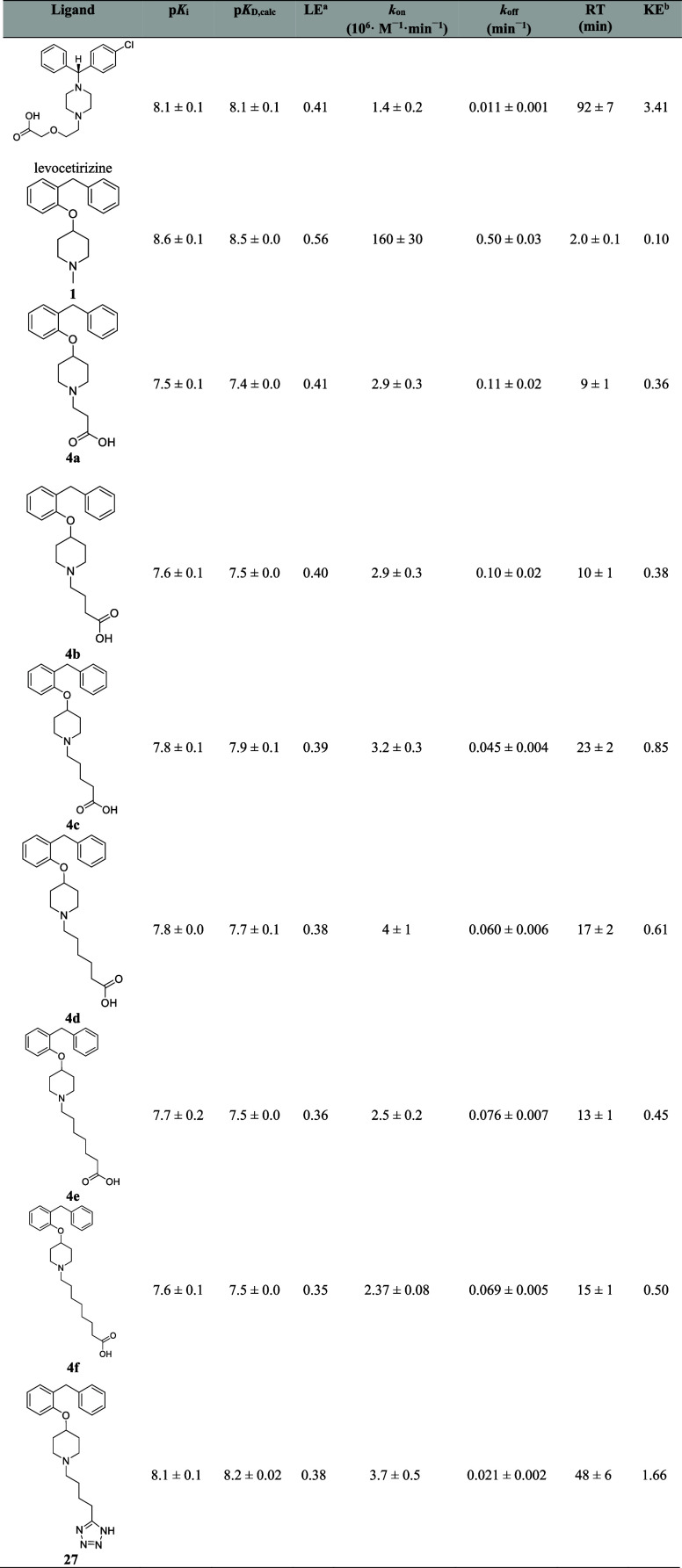
Pharmacological Evaluation of H_1_R Reference Compound Levocetirizine and Ligands Probing the Levocetirizine Binding Region^a^

a Data were obtained from competitive binding experiments to H_1_R using [^3^H]-mepyramine as radioligand. *k*_on_ and *k*_off_ were obtained from the competitive binding of the radioligand over time, and p*K*_D_ and RT values were calculated for individual experiments from these binding rate constants. p*K*_i_ values were obtained from radioligand displacement curves after a 4 h incubation with the radioligand. Data represent the mean and SEM of *N* ≥ 3.

b LE calculated as described in Figure 1.

c KE calculated as described in Figure 1.

### SKR of Ligands Targeting the Olopatadine and Acrivastine Binding Regions

The zwitterionic molecules **2a–c** and **3a–c** show a large decrease in binding affinity (and hence lower LE) and association rate constant compared to amine **1** (Table 1). This observation is in line with the lower affinities and association rate constants of the zwitterions olopatadine and acrivastine compared to their first generation analogs doxepin and triprolidine, respectively (Table 1). Zwitterions **2a–c** and **3a–c** show a wide range of RTs (and KEs) at H_1_R, ranging from 1.2 min for **2a** to 18 min for **2c**. Thus, a 9-fold increase in RT was achieved for compound **2c** compared with first-generation scaffold **1**. The RT of olopatadine increases 7-fold compared to doxepin, whereas a 5-fold increase in RT is observed for acrivastine compared to triprolidine. Benzylic alcohol **2d** has a binding affinity similar to **1**, resulting from a 2-fold decrease of the association rate and a 2-fold increase in RT. On the other hand, benzylic alcohol **3d** has a 10-fold lower binding affinity. Methoxy analog **2e** displays a decrease in binding affinity and association kinetics but has a similar RT compared to **1**. The calculated p*K*_D_ values (i.e., based on the kinetic rate constants) for the tested ligands correspond well with the affinity determined by competition binding experiments (p*K*_i_; Figure 3C).

### SKR Probing the Levocetirizine Binding Region

Upon introducing a carboxylic acid (**4a–f**, Table 2) or a tetrazole (**27**, Table 2) functionality to ligand **1,** the binding affinity (and hence the LE) was decreased as a consequence of a decrease in the association rate constants (40–65-fold), which was larger than the decrease in the dissociation rate constants. Concomitantly with a decreased *k*_off_, the RT (and hence KE) was increased 4.5–24-fold. Table 2 shows that the calculated p*K*_D_ for all ligands (**4a–f**, **27**) correspond well with their equilibrium binding affinity values (p*K*_i_).

## Discussion

**Figure 4:**
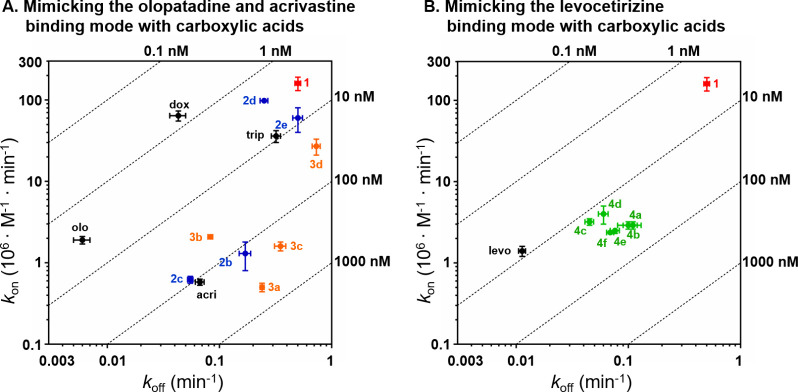
(A) Correlation between the *k*_on_ (*y*-axis) and the *k*_off_ (*x*-axis) for **1**, the ligands probing the olopatadine and acrivastine binding region **2b–e** and **3a–d**, and olopatadine (olo) and acrivastine (acri). Ligands substituted at the 5-position are colored blue (**2b–e**), and ligands substituted at the 6-position are colored orange (**3a–d**). Ligand **2a** is omitted for clarity purposes (*k*_on_: 0.06 × 10^6^ M^–1^·min^–1^; *k*_off_: 1 min^–1^). Parallel dashed lines represent constant *K*_D_ values (*K*_D_ = *k*_off_/*k*_on_) as indicated for the respective lines in the graph. Color of the molecule numbers corresponds to the color-coding in Figure 2A. (B) Correlation between the *k*_on_ (*y*-axis) and the *k*_off_ (*x*-axis) for **1**, the ligands probing the levocetirizine binding region (**4a–f**, green), and levocetirizine (levo). Corresponding pharmacological data can be found in Table 1 and 2.

A set of 16 analogs based on fragment **1** (Figure 2A) has been used to study and modulate H_1_R binding kinetics, by mimicking the binding mode of the long RT binders olopatadine, acrivastine, and levocetirizine (Figure 2B–G). The ligands **2b–e**, **3a–d**, **4a–f**, and H_1_R reference compounds olopatadine, acrivastine, and levocetirizine are plotted in Figure 4 to illustrate their SKR.

### Probing H_1_R Binding Kinetics by Mimicking the Olopatadine and Acrivastine Binding Mode

A carboxylic acid moiety was introduced directly on the phenyl ring (**2a** and **3a**), with a methylene linker to mimic the binding mode of H_1_R reference compound olopatadine (**2b** and **3b**), and with an ethylene linker to mimic the binding mode of acrivastine (**2c** and **3c**). This results in compounds that have a similar or higher RT at H_1_R ranging from 1.2 to 18 min compared to ligand **1**. The compounds with the longest RTs at H_1_R are **2c** (RT: 18 ± 1 min) and **3b** (RT: 12.1 ± 0.5), mimicking the binding mode of acrivastine and olopatadine, respectively. This 6–9 fold increase in RT relative to **1** (RT: 2.0 min) is similar to the relative increase in RTs of the second-generation antihistamines olopatadine and acrivastine compared to their first-generation analogs without a carboxylic acid moiety, i.e. doxepin and triprolidine. Mimicking the acrivastine binding mode, i.e., **2c**, results in a similar RT and KE to acrivastine. However, the ligands that mimic the binding mode of olopatadine (i.e., **2b** and **3b**) do not reach the RT and KE of olopatadine. We speculate that the remarkably long RT and high KE of doxepin and olopatadine are (partially) related to their rigid tricyclic core structure.^26^ The current set of compounds also showed that ligand-binding kinetics optimization is more delicate in the aromatic region, in line with our earlier finding^19^ that optimal placement of the aromatic rings is essential for binding H_1_R with high affinity. The proposed binding modes of **2a** and **3a** suggest that the absence of a linker results in compounds that cannot simultaneously bind favorably in the upper aromatic region and interact with the phosphate pocket residues, and hence show a lower binding affinity and RT. The vector to grow from (i.e., the 5- or 6-position) is also important for binding kinetics as exemplified by the compounds **2b** and **3b** as well as **2c** and **3c** having different RTs and binding affinities. Furthermore, polar nonanionic groups (**2d**, **2e**, and **3d**) growing from the aromatic ring do not significantly change RT at H_1_R.

### Probing H_1_R Binding Kinetics by Mimicking the Levocetirizine Binding Mode

Compounds **4a–f** were designed to mimic the levocetirizine binding mode. By introducing carboxylates linked with spacers of various lengths to the amine group of **1**, the acidic moiety is able to interact with residues within the phosphate pocket (K191^5.39^, K179^45.49^, and H435^7.35^) of H_1_R. Interestingly, all ligands (**4a–f**) have comparable binding kinetics as illustrated in Figure 4, with less than a 3-fold difference in their RT at H_1_R. When decorating this vector of the fragment, having a carboxylate results in low binding rate constants but the position of the carboxylate is not as strict (compare Figure 4A,B). This could potentially be ascribed to protein–ligand interactions that are made during ligand egress from the orthosteric binding site.

On a structural level, the relatively flat SKR for the levocetirizine mimics (Figure 4B) could be explained by the more accessible channel toward the phosphate pocket growing from the basic nitrogen (Figure 2G), compared to growing in the more narrow upper aromatic pocket (Figure 2E,F), which results in a scattered SKR for the olopatadine and acrivastine mimics. This is further supported by a previous study showing that analogs of **1** require optimal placement of the aromatic rings in the aromatic regions for a high binding affinity.^19^

Ligand **4c** shows the highest RT at H_1_R (23 min), mimics the side chain of the levocetirizine, and shows the best overlap in binding mode with levocetirizine of all carboxylic acid levocetirizine mimics (**4a–f**; Supporting Figure 2). However, the low *k*_off_ and hence high RT and KE of levocetirizine binding to H_1_R was not achieved (Figure 4). Gillard et al.^14^ showed that analogs of levocetirizine with an alcohol or ester group instead of the carboxylic acid moiety also have an RT exceeding that of **1** by >5-fold, indicating that the core scaffold of levocetirizine itself is partially responsible for its long RT.

**Figure 5:**
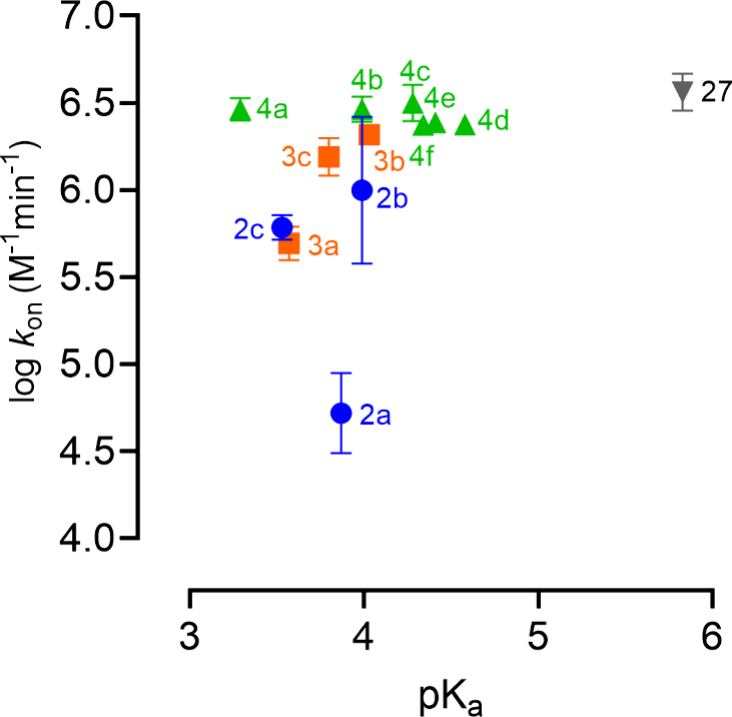
Correlation plot between the log *k*_on_ (*y*-axis) and the p*K*_a_ (*x*-axis) for all analogs of **1** with a p*K*_a_ below 5.5 (**2a–c**, **3a–c**, **4a–f**, **27**). Ligands probing the acrivastine/olopatadine binding region are depicted in blue and orange for analogs of **1** substituted at the 5-position (**2b–e**) or substituted at the 6-position (**3a–d**), respectively. Ligands probing the levocetirizine binding region (**4a–f**) are depicted in green, while **27** is depicted in gray. p*K*_a_ values were calculated using MarvinSketch. Corresponding pharmacological data can be found in Tables 1 and 2.

For all but one (low-affinity compound **2a**) of the carboxylic acid moiety-containing compounds, the dissociation rate is decreased, and hence the RT at H_1_R is increased (Figure 4). However, the decrease in *k*_off_ (1.4-fold to 11.1-fold), relative to **1**, is small compared to the differences observed in the *k*_on_. In fact, all analogs bearing a carboxylic acid moiety have a large decreased *k*_on_ relative to **1** of ≥40-fold. Analogs containing an alcohol (**2d** and **3d**) or a methoxy group (**2e**) instead of a carboxyl acid moiety have much smaller differences in their *k*_on_ values compared to **1** (1.6-fold to 5.9-fold decrease). This could potentially be rationalized as an increase in the desolvation energy for ligand–receptor binding, an effect shown to be rate limiting for ligand association to the β_2_-adrenergic receptor in silico^27^ and in a series inhibitors binding the heat shock protein 90.^28^ To probe this hypothesis, tetrazole **27** was investigated, which has a delocalized charge and has a higher p*K*_a_ value (calculated to be 5.83) than the carboxylic acid analogs **4a–4f** (3.29–4.58) and **27** therefore has a lower expected desolvation energy. However, it becomes clear that the observed differences in the *k*_on_ between our acidic analogs of **1** (**2a–c**, **3a–c**, **4a–f**, **27**) cannot be explained by their calculated p*K*_a_ value (Figure 5). This does not exclude that desolvation of the binding pocket might still be a relevant rate limiting step during ligand association, especially as the carboxylic moiety is likely to interact with positively charged residues (K191^5.39^, K179^45.49^, and H435^7.35^, see Figure 2) that might stabilize water molecules in the unbound pocket. However, the differences in the *k*_on_ observed for e.g. **2c** and **3c**, which have comparable binding modes (Figure 2F), suggests that other factors than the desolvation energy are important to explain these differences, like the shape and flexibility of the molecule and the path of ligand binding.

This study furthermore indicates that optimal placement of the ligand in the different binding regions is essential to maximize the H_1_R binding affinity as well as the H_1_R RT. Based on equilibrium binding affinity values the zwitterions would be deprioritized in a SAR-based ligand optimization, whereas based on their binding kinetics the zwitterions would be prioritized. This study clearly shows that LE is not a preferred metric to describe the quality of the zwitterionic compounds (**2a–c**, **3a–c**, and **4a–f**). Our study emphasizes the importance to measure binding kinetics early on in a drug discovery program and to use the KE metric as an additional selection criterion for subsequent hit and lead optimization, next to the standard equilibrium binding affinity determinations and their corresponding LE values.

In conclusion, a variety of ligands mimicking well-established H_1_R antihistamines olopatadine, acrivastine, and levocetirizine were designed and synthesized. This resulted in 15 compounds of which 12 with longer RTs and larger KEs (e.g., **2c** and **4c**) for binding H_1_R than hit fragment **1**. Introducing carboxylic acid moieties at the 5- and 6-position of the aromatic ring (**2a–e** and **3a–d**), and hence mimicking the binding mode of olopatadine and acrivastine, resulted in large differences in RT (up to 15-fold) for binding to H_1_R. However, for compounds that mimic levocetirizine by growing a carboxylate group from the basic nitrogen atom (**4a–f**), the differences in RTs at H_1_R were less pronounced (up to 3-fold). Ligand **4c**, with four methylene units between the carboxylic acid and the basic nitrogen atom, has the highest RT (23 min) for binding to H_1_R, which is more than a 10-fold increase compared to hit fragment **1**. The obtained SKR shows that the introduction of a carboxylic acid moiety was sufficient to reduce the *k*_on_, which often corresponded with a lower LE but higher KE. However, for the ligands containing a carboxylic acid moiety on the aromatic ring, an optimal KE was only achieved when a high LE was maintained. This could only be accomplished by putting a carboxylic acid group in exactly the right position of the aromatic ring as exemplified by the scattered SKR for the olopatadine and acrivastine mimics (Figure 4A). Interestingly, the introduction of a carboxylic acid group at the basic amine of **1** resulted in less variation in p*K*_D_ and RT at the H_1_R. The latter is illustrated by the ligands **4a**–**f** (Figure 4B), which have a different number of methylene units between the basic nitrogen atom and the carboxylic acid. A structure-based explanation for the observed difference in SKR between the levocetirizine mimics vs the olopatadine and acrivastine mimics is that the latter mimics need to fit in a narrow pocket, whereas the pocket to accommodate the levocetirizine mimics is larger. This study illustrates that carefully designed tool compounds growing from different vectors, and using a variety of linkers, can be used to investigate the structural requirements for binding to GPCRs with an elongated RT, as exemplified for H_1_R.

## Experimental Section

### Pharmacology

#### General Information

Cell culture medium DMEM (Dulbecco’s Modified Eagle Medium) and 1× trypsin solution were obtained from Sigma-Aldrich (St. Louis, USA). Medium supplements fetal bovine serum (FBS) and penicillin/streptomycin were obtained from GE healthcare (Uppsala, Sweden). Transfection reagent linear 25 kDa polyethylenimine (PEI) was bought from Polysciences (Warrington, USA). The Branson sonifier 250 was obtained from Branson Ultrasonics (Danbury, USA). BCA protein assay kit was purchased from Thermo scientific (Waltham, USA). Radioligand binding materials and equipment: [^3^H]-mepyramine, GF/C plates, Microscint-O, the Cell Harvester and the Wallac microbeta were obtained from PerkinElmer (Waltham, USA). The H_1_R reference compounds were obtained from commercial suppliers: doxepin hydrochloride (Tocris Bioscience, *E*/*Z* mixture with a ∼85:15 ratio), triprolidine hydrochloride monohydrate, (Tocris Bioscience), Olopatadine hydrochloride (BOC Sciences), acrivastine (BOC Sciences) and levocetirizine dihydrochloride (Biotrend).

#### DNA Constructs

Mammalian expression vector pcDEF3 containing the N-terminal HA-tagged human H_1_R (GenBank entry AB041380.1), as previously described.^18^

#### Cell Culture and Membrane

HEK293T cells were cultured in DMEM medium supplemented with 10% FBS and 1× penicillin/streptomycin at 37 °C and 5% CO_2_. Cells were lifted using a 1× trypsin solution after which 2 × 10^6^ cells were seeded in a 10 cm^2^ dish. Cells were left overnight and then transfected using a mixture containing 150 mM NaCl, 30 μg linear 25 kDa PEI and 5 μg pcDEF3 encoding human H_1_R as previously described.^29^ After 2 days, cells were collected and washed with a PBS solution (137 mM NaCl, 2.7 mM KCl, 10 mM Na_2_HPO_4_, and 2 mM KH_2_PO_4_) and consecutively stored as dry pellets at −20 °C until further experimentation.

#### Radioligand Binding Experiments

##### Saturation Binding

A frozen cell pellet expressing human H_1_R was reconstituted in 5.5 mL binding buffer (50 mM Na_2_HPO_4_/KH_2_PO_4_ pH 7.4) and homogenized using a Branson sonifier 250 set to a constant duty cycle and a microtip limit of 2, until a homogeneous suspension was obtained (4–8 s). Protein content of membrane homogenates was determined using a BCA protein assay. Increasing concentrations of [^3^H]-mepyramine (0–230 nM) were then incubated with 0.5–10 μg membrane homogenate, depending on the expressed receptor, for 4 h at 25 °C under gentle agitation. This was done both in the absence and presence of 10^–5^ M mianserin, which blocks the specific binding of [^3^H]-mepyramine to human H_1_R. Incubation was terminated by three rapid washing steps using the Cell Harvester with ice-cold Tris–HCl solution (50 mM Trizma base set to pH 7.4 using HCl) over GF/C plates that were coated with 0.5% branched PEI for at least 30 min. Filter plates were then dried in a stove at 52 °C before supplementing microscint-O to the filter plates and measuring radioactivity using a Wallac Microbeta counter. Membrane preparation, termination of the binding reaction and quantifying radioactivity as described here, were performed for all binding experiments.Total binding and nonspecific binding (i.e., in the presence of mianserin) was analyzed by nonlinear regression using eqs 1 (nonspecific binding) and 2 (total binding).

##### Competition Binding

Cell homogenate was incubated with a single concentration 2.5–5.5 nM [^3^H]-mepyramine together with increasing concentration compounds (10^–3^–10^–11^ M) for 4 h at 25 °C under gentle agitation. Binding was analyzed by nonlinear regression using eq 3 to obtain concentrations of unlabeled ligand that displaced 50% of the radioligand (IC_50_). These values were converted to *K*_i_ values using the Cheng–Prusoff equation.^24^

##### Association Binding

Cell homogenate was incubated at 25 °C with four concentrations of [^3^H]-mepyramine between 0.2–4 nM under gentle agitation for (0–41 min) in the absence or presence of 10^–5^ M mianserin. Binding in the presence of mianserin was assumed to be the baseline (i.e., *t* = 0 min). Binding over time was baseline corrected and then analyzed by nonlinear regression using eq 4.

##### Competition Association Binding

Membrane homogenate was incubated with a single concentration 2.5–5.5 nM [^3^H]-mepyramine at 25 °C under gentle agitation for (0–81 min) with either a ± 10 times *K*_i_ equipotent concentration unlabeled ligand or 10^–5^ M mianserin. Binding in the presence of mianserin was assumed to be the baseline (i.e., *t* = 0 min). Binding over time was baseline corrected and then analyzed by nonlinear regression using the Motulsky and Mahan model^25^ (eq 5). This model assumes that both the radioligand and the unlabeled ligand bind competitively to the same binding site according to a one-step binding mechanism.

#### Analysis

Analysis was performed in GraphPad Prism 6.0. Nonlinear regression was performed using the following models:

Total and nonspecific saturation binding
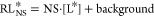
1
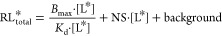
2

logIC_50_
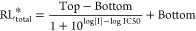
3

Association kinetics using multiple concentrations of ligand
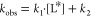

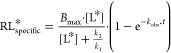
4

Kinetics of competitive binding (constrain *k*_1_ and *k*_2_)
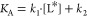

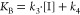

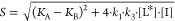

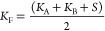

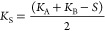

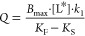

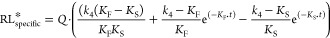
5RL* is the bound [^3^H]-mepyramine and *B*_max_ is the maximally possible specific binding of [^3^H]-mepyramine. [L*] stands for the concentration of [^3^H]mepyramine and [I] for the concentration unlabeled ligand. Association rate constants are denoted by *k*_1_ or *k*_3_ and the dissociation rate constants by *k*_2_ or *k*_4_ for [^3^H]-mepyramine or unlabeled ligand, respectively.

### Computational Methods

#### Preparation of the Ligands, Docking, and Scoring

The 2D-structures of the ligands **2b**, **2c**, **3b**, **3c**, **4c** and the reference compounds olopatadine, acrivastine and levocetirizine were built in ChemBioDraw Ultra 14.0.0.117, and subsequently transformed to SMILES format. Protonation (pH = 7.4) of the SMILES was performed using Chemaxon’s Calculator (version 5.1.4)^30^ and successively converted to MOL2 format using CORINA (version 3.49). PLANTS (version 1.2)^31^ docking was performed in 6-fold at search speed 2 and 25 generated poses per ligand with a clustering RMSD of 2.0. The H_1_R binding site was defined as a radius of 10.8 around the cocrystallized compound Doxepin in the 3RZE crystal structure. As previously reported, a filter was applied that only selects the docking poses possessing an H-bond and ionic interaction with D107^3.32^.^19,32^ The docking pose with the lowest PLANTS ChemPLP score^33^ (i.e., the energetically most favorable pose) was selected.

### Chemistry

#### General Information

THF, DCM and diethyl ether were dried using a PureSolv Micro Multi Unit solvent purification system from Inert. Anhydrous DMF was obtained by storing under activated mol sieves. All other solvents and chemicals were acquired from commercial suppliers and were used as received. ChemBioDraw Ultra 14.0.0.117 was used to generate systematic names for all molecules. All reactions were performed under a nitrogen atmosphere. TLC analyses were carried out with alumina silica plates (Merck F_254_) using staining and/or UV visualization. Column purifications were performed manually using Silicycle Ultra Pure silica gel or automatically using Biotage equipment. Reversed column chromatography was performed using Biotage equipment. The NMR spectra (^1^H, ^13^C, and 2D) were recorded on a Bruker 250 (250 MHz), Bruker 400 (400 MHz) or a Bruker 500 (500 MHz) spectrometer. Chemical shifts are reported in ppm (δ) and the residual solvent was used as internal standard (δ ^1^H NMR: CDCl_3_ 7.26; DMSO-*d*_6_ 2.50; CD_3_OD 3.31; δ ^13^C NMR: CDCl_3_ 77.16; DMSO-*d*_6_ 39.52; CD_3_OD 49.00). Data are reported as follows: chemical shift (integration, multiplicity (s = singlet, d = doublet, t = triplet, q = quartet, br = broad signal, m = multiplet), and coupling constants (Hz)). A Bruker microTOF mass spectrometer using ESI in positive ion mode was used to record HRMS spectra. A Shimadzu LC-20AD liquid chromatograph pump system linked to a Shimadzu SPD-M20A diode array detector with MS detection using a Shimadzu LC-MS-2010EV mass spectrometer was used to perform LC-MS analyses. An Xbridge (C18) 5 μm column (50, 4.6 mm) was used. The solvents that were used were the following: solvent B (acetonitrile with 0.1% formic acid) and solvent A (water with 0.1% formic acid), flow rate of 1.0 mL/min, start 5% B, linear gradient to 90% B in 4.5 min, then 1.5 min at 90% B, then linear gradient to 5% B in 0.5 min, then 1.5 min at 5% B; total run time of 8 min. All compounds have a purity of ≥95% (unless specified otherwise), calculated as the percentage peak area of the analyzed compound by UV detection at 230 nm (values are rounded). Notably, fumaric acid is also visible by UV (retention time ∼1 min).

##### 4-Benzyl-3-((1-methylpiperidin-4-yl)oxy)benzoic Acid (2a)

A mixture of ester **7** (120 mg, 0.35 mmol) and aqueous 1.0 M NaOH (0.530 mL, 0.53 mmol) in MeOH (5 mL) was stirred at reflux for 5 h. The volatiles were evaporated in vacuo. Purification by reversed phase column chromatography (H_2_O/MeCN 10:0 to 3:7) gave the title compound as a white solid (38 mg, 33%). ^1^H NMR (500 MHz, DMSO-*d*_6_) δ 7.47–7.41 (m, 2H), 7.29–7.23 (m, 3H), 7.23–7.19 (m, 2H), 7.18–7.13 (m, 1H), 4.51–4.40 (m, 1H), 3.93 (s, 2H), 2.48–2.38 (br, 2H, overlaps with solvent peak), 2.27–2.18 (m, 2H), 2.14 (s, 3H), 1.92–1.78 (m, 2H), 1.70–1.55 (m, 2H). ^13^C NMR (126 MHz, DMSO-*d*_6_ and one drop of D_2_O) δ 170.1, 154.2, 141.6, 137.3, 132.8, 130.9, 129.2, 129.0, 126.6, 122.3, 113.7, 68.5 (confirmed by HSQC), 51.1, 44.3, 36.5, 28.9. HR-MS [M + H]^+^ calcd for C_20_H_24_NO_3_^+^: 326.1751, found: 326.1754. HR-MS [M–H]^−^ calcd for C_20_H_22_NO_3_^–^: 324.1605, found: 324.1616.

##### 2-(4-Benzyl-3-((1-methylpiperidin-4-yl)oxy)phenyl)acetic Acid (2b)

Enol ether deprotection: a solution of amine **13** (150 mg, 0.45 mmol) in dioxane (5.0 mL) and 3 drops of water was cooled in an ice-bath briefly ensuring that dioxane did not freeze. Subsequently, 4 M HCl in dioxane (0.278 mL, 1.11 mmol) was added and the resulting mixture was stirred for 45 min at room temperature. LC-MS indicated full conversion to aldehyde **14** and the reaction mixture was used in the next step without any workup. Pinnick oxidation: KH_2_PO_4_.H_2_O (307 mg, 2.23 mmol) was dissolved in water (1.0 mL) and sodium chlorite (322 mg, 2.23 mmol) was added which led to gas evolution. This was added to the reaction mixture containing aldehyde **14** to which had been added *t*-BuOH (2.5 mL) and 2-methylbut-2-ene (1.65 mL, 15.6 mmol). The reaction mixture was stirred for 45 min at room temperature. The reaction mixture was added dropwise to a 1.0 M aqueous HCl solution (100 mL) and extracted with CHCl_3_ (5 × 40 mL). The combined organic phases were dried over Na_2_SO_4_, filtered and reduced in vacuo. Purification by reversed phase column chromatography (H_2_O/MeCN 10:0 to 3:7) gave the title compound as a white solid (9 mg, 6% over two steps, i.e., from **13**). ^1^H NMR (500 MHz, DMSO-*d*_6_) δ 12.90–10.97 (br, 1H), 7.28–7.22 (m, 2H), 7.21–7.17 (m, 2H), 7.17–7.12 (m, 1H), 7.10 (d, *J* = 7.6 Hz, 1H), 6.87 (s, 1H), 6.74 (dd, *J* = 7.6, 1.2 Hz, 1H), 4.44–4.34 (br, 1H), 3.86 (s, 2H), 3.50 (s, 2H), 2.52 (br, 2H, overlaps with solvent peak), 2.41–2.26 (br, 2H), 2.22 (s, 3H), 1.93–1.81 (m, 2H), 1.70–1.57 (m, 2H). ^13^C NMR (126 MHz, DMSO-*d*_6_) δ 172.8, 154.2, 141.2, 134.4, 130.3, 128.7, 128.3 (confirmed by D_2_O shake), 128.2, 125.7, 121.2, 114.1, 70.0 (confirmed by HSQC), 51.5, 45.3, 40.6, 35.5, 29.8. HR-MS [M + H]^+^ calcd for C_21_H_26_NO_3_^+^: 341.1907, found: 340.1910. HR-MS [M–H]^−^ calcd for C_21_H_24_NO_3_^–^: 338.1762, found: 338.1773.

##### (*E*)-3-(4-Benzyl-3-((1-methylpiperidin-4-yl)oxy)phenyl)acrylic Acid (2c)

Wittig reaction: to a cooled (0 °C) suspension of aldehyde **15** (390 mg) in toluene (10 mL) was added methyl 2-(triphenylphosphoranylidene)acetate (506 mg, 1.51 mmol). The resulting mixture was stirred for 48 h at room temperature and evaporated in vacuo. Purification by flash column chromatography (DCM/MeOH 10:0 to 9:1) gave a mixture (400 mg) of the corresponding ester product and PPh_3_O in a 1:0.6 molar ratio as determined by ^1^H NMR analysis. The product mixture was used in the next step without further purification. ^1^H NMR (500 MHz, CDCl_3_) δ 7.66–7.62 (d, *J* = 16.0 Hz (suggesting the *E*-isomer), 1H, overlaps with the PPh_3_O signal), 7.30–7.23 (m, 2H, overlaps with solvent peak), 7.22–7.15 (m, 4H), 7.11–7.05 (m, 1H), 6.97–6.91 (m, 1H), 6.37 (d, *J* = 16.0 Hz (suggesting the *E*-isomer), 1H), 4.56–4.35 (m, 1H), 3.98 (s, 2H), 3.81 (s, 3H), 2.66–2.33 (m, 4H), 2.29 (s, 3H), 2.19–1.99 (m, 2H), 1.92–1.80 (m, 2H). Saponification: the obtained mixture containing the ester (392 mg) and aqueous 2.0 M NaOH (0.555 mL, 1.11 mmol) in MeOH (10 mL) were stirred at reflux for 6 h. The volatiles were evaporated in vacuo. Purification by reversed phase column chromatography (H_2_O/MeCN 10:0 to 7:3) gave the title compound as a white solid (50 mg, 7% over three steps, i.e., from **2d**). ^1^H NMR (500 MHz, DMSO-*d*_6_) δ 7.51 (d, *J* = 16.0 Hz (suggesting the *E*-isomer), 1H), 7.30 (s, 1H), 7.28–7.22 (m, 2H), 7.22–7.18 (m, 3H), 7.18–7.12 (m, 2H), 6.53 (d, *J* = 16.0 Hz (suggesting the *E*-isomer), 1H), 4.59–4.48 (m, 1H), 3.89 (s, 2H), 2.48–2.40 (br, 2H, overlaps with solvent peak), 2.24–2.15 (m, 2H), 2.13 (s, 3H), 1.90–1.81 (m, 2H), 1.65–1.52 (m, 2H). ^13^C NMR (126 MHz, DMSO-*d*_6_) δ 167.9, 154.9, 143.6, 140.7, 134.0, 132.4, 130.9, 128.7, 128.2, 125.8, 120.5, 119.4, 112.0, 70.6 (confirmed by HSQC), 52.0, 45.9, 35.7, 30.3. HR-MS [M + H]^+^ calcd for C_22_H_26_NO_3_^+^: 352.1907, found: 352.1904. HR-MS [M – H]^−^ calcd for C_22_H_24_NO_3_^–^: 350.1762, found: 350.1774.

##### (4-Benzyl-3-((1-methylpiperidin-4-yl)oxy)phenyl)methanol Hemifumarate (2d)

To a cooled (0 °C) solution of aldehyde **11** (200 mg, 0.51 mmol) in THF (10 mL) was added dropwise a 1.0 M LAH in THF solution (0.51 mL, 0.51 mmol). The resulting mixture was stirred for 2 h at 60 °C. The reaction mixture was cooled to room temperature, quenched with 2.0 M aqueous NaOH (100 mL) and extracted with DCM (3 × 40 mL). The combined organic phases were dried over Na_2_SO_4_, filtered and evaporated in vacuo. Purification by flash column chromatography (EtOAc/MeOH/TEA 95:2.5:2.5 to 90:5:5) gave a colorless oil (145 mg, 92%). The free base (140 mg, 0.45 mmol) was converted to a hemifumaric acid salt, with the method described by Kuhne et al.^19^ to obtain the title compound as a white solid (63 mg, 38%). ^1^H NMR (500 MHz, DMSO-*d*_6_) δ 7.27–7.21 (m, 2H), 7.20–7.10 (m, 4H), 6.92 (s, 1H), 6.81 (d, *J* = 7.6 Hz, 1H), 6.53 (s, 1H), 5.08 (br, 1H, D_2_O exchangeable), 4.49–4.38 (m, 3H), 3.87 (s, 2H), 2.65–2.53 (m, 2H), 2.49–2.38 (m, 2H, overlaps with solvent peak), 2.30–2.23 (m, 3H), 1.95–1.85 (m, 2H), 1.74–1.61 (m, 2H). ^13^C NMR (126 MHz, DMSO-*d*_6_) δ 167.2, 154.3, 142.2, 141.4, 134.7, 130.4, 128.5, 128.9 (confirmed by D_2_O shake), 128.2, 125.7, 118.3, 111.0, 69.2 (confirmed by HSQC), 62.9, 51.1, 44.7, 35.6, 29.3. HR-MS [M + H]^+^ calcd for C_20_H_26_NO_2_^+^: 312.1958, found: 312.1944.

##### 4-(2-Benzyl-5-methoxyphenoxy)-1-methylpiperidine Fumarate (2e)

Phenyl ether formation: phenol **17** (4.85 g, 22.6 mmol) was dissolved in DMF (65 mL). Subsequently, *tert*-butyl 4-((methylsulfonyl)oxy)piperidine-1-carboxylate (18.77 g, 67.2 mmol) and Cs_2_CO_3_ (19.18 g, 58.9 mmol) were added and the resulting mixture was stirred for 26 h at 65 °C. The mixture was cooled to room temperature, diluted with water (200 mL) and extracted with EtOAc (150 mL). The organic layer was washed with saturated aqueous NaHCO_3_ solution, dried over Na_2_SO_4_, filtered and evaporated in vacuo. Purification by flash column chromatography ((*n*-heptane/TEA)/EtOAc 100:0 to 75:25(99:1)) gave a mixture (11.76 g) of the alkylated phenol and the elimination product (*tert*-butyl 3,6-dihydropyridine-1(2*H*)-carboxylate) in a 1:0.7 molar ratio as determined by ^1^H NMR analysis. The product mixture was used in the next step without further purification. Reduction: to a cooled (0 °C) solution of 1.0 M LAH in THF (7.77 mL, 7.77 mmol) was added the mixture containing the alkylated phenol (1.01 g) in THF (16 mL). The resulting mixture was stirred for 4 h at room temperature and 96 h at 50 °C. The reaction mixture was cooled to 0 °C, quenched with 1.0 M aqueous NaOH and extracted with DCM (3 × 70 mL). The combined organic phases were dried over Na_2_SO_4_, filtered and evaporated in vacuo to afford the title compound (325 mg, 54% over two steps, i.e., from **17**). The free base (325 mg, 1.04 mmol) was converted to a fumaric acid salt, with the method described by Kuhne et al.^19^ to obtain the title compound as a white solid (327 mg, 73%). ^1^H NMR (500 MHz, DMSO-*d*_6_) δ 7.26–7.22 (m, 2H), 7.19–7.11 (m, 3H), 7.08 (d, *J* = 8.3 Hz, 1H), 6.57–6.55 (m, 2H), 6.53 (s, 1H), 6.45 (dd, *J* = 8.3, 2.3 Hz, 1H), 4.50–4.43 (m, 1H), 3.82 (s, 2H), 3.71 (s, 3H), 2.58–2.43 (m, 4H, overlaps with solvent peak), 2.27 (s, 3H), 1.93–1.85 (m, 2H), 1.71–1.62 (m, 2H). ^13^C NMR (126 MHz, DMSO-*d*_6_) δ 166.7, 159.1, 155.1, 141.6, 134.4, 131.0, 128.4, 128.1, 125.6, 122.1, 104.7, 100.2, 69.0 (confirmed by HSQC), 55.1, 50.9, 44.6, 35.2, 29.1. HR-MS [M + H]^+^ calcd for C_20_H_26_NO_2_^+^: 312.1958, found: 312.1956.

##### 3-Benzyl-2-((1-methylpiperidin-4-yl)oxy)benzoic Acid (3a)

KH_2_PO_4_·H_2_O (557 mg, 4.04 mmol) was dissolved in water (2.0 mL) and sodium chlorite (365 mg, 4.04 mmol) in water (2.0 mL) was added which led to gas evolution. This was added to a mixture containing aldehyde **21** (250 mg, 0.81 mmol) to which had been added *t*-BuOH (3.0 mL) and 2-methylbut-2-ene (4.29 mL, 40.4 mmol). The reaction mixture was stirred for 2 h at room temperature. The reaction mixture was taken up in DCM (25 mL) and 1.0 M aqueous HCl (75 mL) and the phases were separated. The aqueous phase was extracted with DCM (5 × 25 mL). The combined organic phases were dried over Na_2_SO_4_, filtered and reduced in vacuo. Purification by reversed phase column chromatography (H_2_O/MeCN 10:0 to 4:6) gave the title compound as a white solid (6 mg, 2%). ^1^H NMR (500 MHz, DMSO-*d*_6_) δ 7.43 (dd, *J* = 7.6, 1.6 Hz, 1H), 7.30–7.25 (m, 2H), 7.24–7.15 (m, 4H), 7.03 (t, *J* = 7.6 Hz, 1H), 4.03–3.90 (m, 3H), 2.75–2.66 (m, 2H), 2.16 (s, 3H), 2.04–1.90 (m, 2H), 1.86–1.78 (m, 2H), 1.75–1.60 (m, 2H). ^13^C NMR (126 MHz, DMSO-*d*_6_) δ 168.8, 153.6, 140.8, 135.1, 132.9, 128.8, 128.6, 128.4, 126.0, 122.8, 79.6, 53.0, 45.4, 35.5, 31.2. One quaternary carbon was not detected. HR-MS [M + H]^+^ calcd for C_20_H_24_NO_3_^+^: 326.1751, found: 326.1739. HR-MS [M – H]^−^ calcd for C_20_H_22_NO_3_^–^: 324.1605, found: 324.1608.

##### 2-(3-Benzyl-2-((1-methylpiperidin-4-yl)oxy)phenyl)acetic Acid (3b)

Enol ether deprotection: a solution of enol ether **22** (240 mg, 0.71 mmol) in dioxane (6.0 mL) and 3 drops of water was cooled in an ice-bath briefly ensuring that dioxane did not freeze. Subsequently, 4 M HCl in dioxane (0.445 mL, 1.78 mmol) was added and the resulting mixture was stirred for 1 h at room temperature. LC-MS indicated full conversion to aldehyde **23** and the reaction mixture was used in the next step without any workup. Pinnick oxidation: KH_2_PO_4_·H_2_O (491 mg, 3.56 mmol) was dissolved in water (1.0 mL) and sodium chlorite (322 mg, 3.56 mmol) in water (0.5 mL) was added which led to gas evolution. This was added to the reaction mixture containing aldehyde **23** to which had been added *t*-BuOH (2.5 mL) and 2-methylbut-2-ene (3.78 mL, 35.6 mmol). The reaction mixture was stirred for 1 h at room temperature. The reaction mixture was added dropwise to a 1.0 M aqueous HCl solution (150 mL) and extracted with CHCl_3_ (8 × 30 mL). The combined organic phases were dried over Na_2_SO_4_, filtered and reduced in vacuo. Purification by reversed phase column chromatography (H_2_O/MeCN 10:0 to 3:7) gave the title compound as a white solid (22 mg, 9% over 2 steps). ^1^H NMR (500 MHz, DMSO-*d*_6_) δ 7.29–7.25 (m, 2H), 7.20–7.15 (m, 3H), 7.13–7.09 (m, 1H), 7.00–6.96 (m, 2H), 3.97 (s, 2H), 3.77–3.70 (m, 1H), 3.57 (s, 2H), 2.74–2.66 (m, 2H), 2.11 (s, 3H), 1.89–1.76 (m, 4H), 1.73–1.63 (m, 2H). ^13^C NMR (126 MHz, DMSO-*d*_6_) δ 172.9, 153.7, 140.9, 134.0, 129.8, 129.6, 129.0, 128.7, 128.4, 125.9, 123.4, 79.5, 53.4, 45.6, 36.1, 35.7, 31.6. HR-MS [M + H]^+^ calcd for C_21_H_26_NO_3_^+^: 340.1907, found: 340.1911. HR-MS [M – H]^−^ calcd for C_21_H_24_NO_3_^–^: 338.1762, found: 338.1769.

##### (*E*)-3-(3-Benzyl-2-((1-methylpiperidin-4-yl)oxy)phenyl)acrylic Acid (3c)

Wittig reaction: to a solution of aldehyde **21** (250 mg, 0.81 mmol) in THF (8 mL) was added methyl 2-(triphenylphosphoranylidene)acetate (324 mg, 0.97 mmol). The resulting mixture was stirred for 48 h at room temperature and evaporated in vacuo. Purification by flash column chromatography (DCM/MeOH 10:0 to 9:1) gave a mixture (330 mg) of the corresponding ester and PPh_3_O in a 1:0.64 molar ratio as determined by ^1^H NMR analysis. The product mixture was used in the next step without further purification. ^1^H NMR (250 MHz, DMSO-*d*_6_) δ 7.93 (d, *J* = 16.2 Hz (suggesting the *E*-isomer), 1H), 7.35–7.05 (m, 8H), 6.60 (d, *J* = 16.2 Hz (suggesting the *E*-isomer), 1H), 4.01 (s, 2H), 3.82–3.66 (m, 4H), 2.75–2.63 (m, 2H), 2.11 (s, 3H), 1.89–1.64 (m, 6H). Saponification: the obtained mixture containing the ester (330 mg) and aqueous 2.0 M NaOH (0.454 mL, 0.91 mmol) in MeOH (10 mL) were stirred at reflux for 6 h. The volatiles were evaporated in vacuo. Purification by reversed phase column chromatography (H_2_O/MeCN 10:0 to 3:7) gave the title compound as a white solid (69 mg, 24% over two steps, i.e., from **21**). ^1^H NMR (500 MHz, DMSO-*d*_6_) δ 7.84 (d, *J* = 16.1 Hz (suggesting the *E*-isomer), 1H), 7.63 (dd, *J* = 7.8, 1.6 Hz, 1H), 7.32–7.24 (m, 2H), 7.22–7.14 (m, 4H), 7.09 (t, *J* = 7.6 Hz, 1H), 6.47 (d, *J* = 16.1 Hz (suggesting the *E*-isomer), 1H), 4.00 (s, 2H), 3.78–3.66 (m, 1H), 2.75–2.66 (m, 2H), 2.12 (s, 3H), 1.92–1.78 (m, 4H), 1.77–1.65 (m, 2H). ^13^C NMR (126 MHz, DMSO-*d*_6_) δ 167.8, 154.4, 140.6, 139.0, 135.1, 132.9, 128.7, 128.6, 128.4, 126.0, 125.6, 124.2, 120.0, 80.8, 53.1, 45.5, 35.3, 31.5. HR-MS [M + H]^+^ calcd for C_22_H_26_NO_3_^+^: 352.1907, found: 352.1911. HR-MS [M–H]^−^ calcd for C_22_H_24_NO_3_^–^: 350.1762, found: 350.1761.

##### (3-Benzyl-2-((1-methylpiperidin-4-yl)oxy)phenyl)methanol (3d)

To a cooled (0 °C) solution of 1.0 M LAH in THF (4.17 mL, 4.17 mmol) was added dropwise aldehyde **20** (1.10 g, 2.78 mmol) in THF (12 mL). The resulting mixture was stirred at reflux for 4 h. The reaction mixture was cooled to room temperature, quenched with 1.0 M aqueous NaOH (300 mL) and extracted with DCM (3 × 70 mL). The combined organic phases were dried over Na_2_SO_4_, filtered and evaporated in vacuo. Purification by flash column chromatography (EtOAc/MeOH/TEA 95:2.5:2.5) gave the title product as white solid (150 mg, 17%). ^1^H NMR (500 MHz, DMSO-*d*_6_) δ 7.31 (dd, *J* = 7.3, 1.7 Hz, 1H), 7.29–7.24 (m, 2H), 7.19–7.14 (m, 3H), 7.04–6.96 (m, 2H), 5.09 (t, *J* = 5.6 Hz, 1H), 4.52 (d, *J* = 5.6 Hz, 2H), 3.96 (s, 2H), 3.82–3.74 (m, 1H), 2.73–2.64 (m, 2H), 2.12 (s, 3H), 1.90–1.80 (m, 4H), 1.72–1.62 (m, 2H). ^13^C NMR (126 MHz, DMSO-*d*_6_) δ 152.6, 141.1, 135.7, 133.7, 129.3, 128.7, 128.3, 126.8, 125.8, 123.4, 79.5, 58.2, 53.4, 45.7, 35.6, 31.8. HR-MS [M + H] ^+^ calcd for C_20_H_26_NO_2_^+^: 312.1958, found: 312.1965.

##### 3-(4-(2-Benzylphenoxy)piperidin-1-yl)propanoic Acid Hydrochloride (4a)

A mixture of ester **25a** (190 mg, 0.52 mmol) and aqueous 2.0 M NaOH (0.284 mL, 0.57 mmol) in MeOH (6.0 mL) was stirred at reflux for 3 h. The volatiles were evaporated and 1.0 M aqueous HCl (∼25 mL) was added to the formed oil. The mixture was stirred vigorously at room temperature for approximately 30 min. A precipitate was formed, filtered off and washed with 1.0 M aqueous HCl. The white solid was dried in vacuo to give the title compound (74 mg, 38%). ^1^H NMR (500 MHz, D_2_O/DMSO-*d*_6_ 0.15/0.45 mL, 4.99 mg **4a**, 4.99 mg K_2_CO_3_) δ 7.25–7.01 (m, 7H), 6.90–6.84 (m, 1H), 6.83–6.78 (m, 1H), 4.59–4.53 (no HSQC signal present, assigned as water peak), 4.35–4.26 (br, 1H), 3.81 (s, 2H), 2.48–2.35 (m, 4H), 2.31–2.20 (br, 2H), 2.19–2.11 (m, 2H), 1.81–1.69 (m, 2H), 1.65–1.54 (m, 2H).^13^C NMR (126 MHz, D_2_O/DMSO-*d*_6_ 0.15/0.45 mL, 4.99 mg **4a**, 4.99 mg K_2_CO_3_) δ 181.6, 163.9, 156.0, 142.9, 132.5, 132.2, 130.3, 130.0, 129.4, 127.6, 122.5, 115.4, 72.6 (confirmed by HSQC), 55.9, 50.3, 37.4, 36.2, 31.0. HR-MS [M + H]^+^ calcd for C_21_H_26_NO_3_^+^: 340.1907, found 340.1896.

##### 4-(4-(2-Benzylphenoxy)piperidin-1-yl)butanoic Acid Hydrochloride (4b)

This compound was synthesized according to the procedure of **4a** using ester **25b** (245 mg, 0.64 mmol), 2.0 M aqueous NaOH (0.353 mL, 0.71 mmol), MeOH (6.0 mL) and a reflux time of 3 h. The title compound (124 mg, 50%) was obtained as a white solid. ^1^H NMR (500 MHz, D_2_O/DMSO-*d*_6_ 0.15/0.45 mL, 5.12 mg **4b**, 5.45 mg K_2_CO_3_) δ 7.23–6.99 (m, 7H), 6.87 (d, *J* = 8.2 Hz, 1H), 6.81 (t, *J* = 7.4 Hz, 1H), 4.60–4.52 (no HSQC signal present, assigned as water peak), 4.36–4.29 (m, 1H), 3.80 (s, 2H), 2.45–2.19 (m, 4H), 2.18–2.10 (m, 2H), 1.95 (t, *J* = 7.5 Hz, 2H), 1.79–1.69 (m, 2H), 1.65–1.48 (m, 4H). ^13^C NMR (126 MHz, D_2_O/DMSO-*d*_6_ 0.15/0.45 mL, 5.12 mg **4b**, 5.45 mg K_2_CO_3_) δ 183.1, 163.7, 156.0, 142.9, 132.6, 132.1, 130.3, 130.0, 129.4, 127.6, 122.5, 115.3, 72.8 (confirmed by HSQC), 58.9, 50.4, 37.4, 37.2, 30.8, 24.2. HR-MS [M + H]^+^calcd for C_22_H_28_NO_3_^+^: 354.2064, found 354.2058.

##### 5-(4-(2-Benzylphenoxy)piperidin-1-yl)pentanoic Acid Hydrochloride (4c)

This compound was synthesized according to the procedure of **4a** using ester **25c** (188 mg, 0.48 mmol), 2.0 M aqueous NaOH (0.261 mL, 0.52 mmol), MeOH (6.0 mL) and a reflux time of 2.5 h. The title compound (75 mg, 39%) was obtained as a white solid. ^1^H NMR (500 MHz, D_2_O/DMSO-*d*_6_ 0.15/0.45 mL, 5.09 mg **4c**, 5.29 mg K_2_CO_3_) δ 7.19–7.02 (m, 7H), 6.89–6.84 (m, 1H), 6.84–6.78 (m, 1H), 4.60–4.55 (no HSQC signal present, assigned as water peak), 4.38–4.28 (br, 1H), 3.80 (s, 2H), 2.41–2.09 (m, 6H), 2.01–1.94 (m, 2H), 1.77–1.67 (m, 2H), 1.65–1.54 (m, 2H), 1.40–1.24 (m, 4H). ^13^C NMR (126 MHz, D_2_O/DMSO-*d*_6_ 0.15/0.45 mL, 5.09 mg **4c**, 5.29 mg K_2_CO_3_) δ 183.7, 164.2, 156.0, 142.9, 132.6, 132.0, 130.3, 130.0, 129.4, 127.6, 122.4, 115.3, 72.5 (confirmed by HSQC), 59.0, 50.4, 39.0, 37.4, 30.8, 27.1, 25.6. HR-MS [M + H]^+^ calcd for C_23_H_30_NO_3_^+^: 368.2220, found 368.2208. HR-MS [M – H]^−^ calcd for C_23_H_28_NO_3_^–^: 366.2075, found: 366.2073.

##### 6-(4-(2-Benzylphenoxy)piperidin-1-yl)hexanoic Acid Hydrochloride (4d)

This compound was synthesized according to the procedure of **4a** using ester **25d** (191 mg, 0.47 mmol), 2.0 M aqueous NaOH (0.256 mL, 0.51 mmol), MeOH (6.0 mL) and a reflux time of 3 h The title compound (75 mg, 42%) was obtained as a white solid. ^1^H NMR (500 MHz, D_2_O/DMSO-*d*_6_ 0.15/0.45 mL, 5.03 mg **4d**, 5.06 mg K_2_CO_3_) δ 7.16–7.00 (m, 7H), 6.84 (d, *J* = 8.2 Hz, 1H), 6.80–6.76 (m, 1H), 4.60–4.53 (no HSQC signal present, assigned as water peak), 4.36–4.27 (br, 1H), 3.78 (s, 2H), 2.36–2.05 (m, 6H), 1.97 (t, *J* = 7.5 Hz, 2H), 1.76–1.66 (m, 2H), 1.65–1.54 (m, 2H), 1.42–1.34 (m, 2H), 1.32–1.23 (m, 2H), 1.14–1.04 (m, 2H). ^13^C NMR (126 MHz, D_2_O/DMSO-*d*_6_ 0.15/0.45 mL, 5.03 mg **4d**, 5.06 mg K_2_CO_33_) δ 183.9, 163.8, 156.0, 142.9, 132.6, 131.9, 130.2, 130.0, 129.4, 127.6, 122.4, 115.2, 72.4 (confirmed by HSQC), 59.2, 50.3, 39.1, 37.4, 30.7, 28.5, 27.3, 26.9. HR-MS [M + H]^+^ calcd for C_24_H_32_NO_3_^+^: 382.2377, found 382.2366.

##### 7-(4-(2-Benzylphenoxy)piperidin-1-yl)heptanoic Acid Hydrochloride (4e)

A mixture of ester **25e** (195 mg, 0.46 mmol) and aqueous 2.0 M NaOH (0.253 mL, 0.51 mmol) in MeOH (5.0 mL) was stirred at reflux for 3 h. The volatiles were evaporated and the formed oil was diluted with 1.0 M aqueous HCl (25 mL) and extracted with CHCl_3_ (3 × 25 mL). The combined organic phases were dried over Na_2_SO_4_, filtered and evaporated in vacuo. A 1.0 M aqueous HCl solution (25 mL) was added to the oil and the mixture was stirred vigorously at room temperature for approximately 30 min. A precipitate was formed, filtered off and washed with 1.0 M aqueous HCl. The white solid was dried in vacuo to give the title compound (60 mg, 30%). ^1^H NMR (500 MHz, D_2_O/DMSO-*d*_6_ 0.15/0.45 mL, 5.03 mg **4e**, 4.92 mg K_2_CO_3_) δ 7.05–6.90 (m, 7H), 6.74 (d, *J* = 8.2 Hz, 1H), 6.66 (t, *J* = 7.4 Hz, 1H), 4.60–4.52 (no HSQC signal present, assigned as water peak), 4.30–4.18 (br, 1H), 3.70 (s, 2H), 2.28–2.08 (br, 4H), 2.06–2.00 (m, 2H), 1.95 (t, *J* = 7.6 Hz, 2H), 1.72–1.59 (m, 2H), 1.59–1.48 (m, 2H), 1.40–1.30 (m, 2H), 1.27–1.18 (m, 2H), 1.14–1.02 (m, 4H). ^13^C NMR (126 MHz, D_2_O/DMSO-*d*_6_, 0.15/0.45 mL, 5.03 mg **4e**, 4.92 mg K_2_CO_3_) δ 183.9, 163.9, 156.0, 142.7, 132.4, 131.7, 130.1, 129.8, 129.3, 127.4, 122.1, 114.9, 72.0 (confirmed by HSQC), 59.4, 50.2, 39.2, 37.4, 30.7, 30.3, 28.4, 27.4, 27.0. HR-MS [M + H]^+^ calcd for C_25_H_34_NO_3_^+^: 396.2533, found 396.2520.

##### 8-(4-(2-Benzylphenoxy)piperidin-1-yl)octanoic Acid Hydrochloride (4f)

A mixture of ester **25f** (190 mg, 0.43 mmol) and aqueous 2.0 M NaOH (0.239 mL, 0.48 mmol) in MeOH (6.0 mL) was stirred at reflux for 3 h. The volatiles were evaporated and the formed oil was dissolved in a 1.0 M aqueous HCl solution (45 mL). The mixture was stirred vigorously. A white/yellowish precipitate was formed and the mixture was evaporated to dryness. Subsequently, a 1.0 M aqueous HCl solution (45 mL) was added and the resulting mixture was stirred vigorously at room temperature for approximately 30 min to give a suspension. The supernatant was decanted. The remaining wet solid was suspended in 1.0 M aqueous HCl and was filtered off and subsequently washed with 1.0 M aqueous HCl. The white solid was dried in vacuo to give the title compound (63 mg, 33%). ^1^H NMR (400 MHz, D_2_O/DMSO-*d*_6_ 0.15/0.45 mL, 5.04 mg **4f**, 4.81 mg K_2_CO_3_) δ 6.98–6.81 (m, 7H), 6.68 (d, *J* = 8.3 Hz, 1H), 6.60 (t, *J* = 7.4 Hz, 1H), 4.61–4.51 (no HSQC signal present, assigned as water peak), 4.24–4.13 (m, 1H), 3.66 (s, 2H), 2.24–2.04 (m, 4H), 2.03–1.87 (m, 4H), 1.69–1.44 (m, 4H), 1.42–1.28 (m, 2H), 1.26–1.15 (m, 2H), 1.14–0.95 (m, 6H). ^13^C NMR (101 MHz D_2_O/DMSO-*d*_6_ 0.15/0.45 mL, 5.04 mg **4f**, 4.81 mg K_2_CO_3_) δ 183.7, 163.5, 156.0, 142.6, 132.3, 131.5, 130.1, 129.7, 129.2, 127.3, 121.9, 114.6, 71.7 (confirmed by HSQC), 59.5, 50.1, 39.2 (confirmed by HSQC, overlaps with solvent peak), 37.5, 30.7, 30.5, 30.3, 28.6, 27.5, 27.2. HR-MS (EIMS): [M + H]^+^ calcd for C_26_H_36_NO_3_^+^: 410.2690, found 410.2681.

##### Methyl 4-Benzyl-3-methoxybenzoate (5)

Benzyl bromide **4** (24.0 g, 93 mmol) was dissolved in benzene (500 mL). Subsequently, aluminum trichloride (37.1 g, 278 mmol) was added in portions. The resulting mixture was stirred for 165 min at room temperature. The reaction mixture was poured carefully into ice–water with vigorous stirring and extracted with EtOAc (2 × 200 mL). The combined organic phases were washed with brine (200 mL), dried over Na_2_SO_4_, filtered and reduced in vacuo. The yellow oil was precipitated from *n*-heptane. This gave the title compound as slightly yellow solid (17.22 g, 73%). ^1^H NMR (500 MHz, CDCl_3_) δ 7.57 (dd, *J* = 7.8, 1.4 Hz, 1H), 7.52 (d, *J* = 1.4 Hz, 1H), 7.30–7.25 (m, 2H, overlaps with solvent peak), 7.24–7.16 (m, 3H), 7.11 (d, *J* = 7.8 Hz, 1H), 4.01 (s, 2H), 3.90 (s, 3H), 3.88 (s, 3H). ^13^C NMR (126 MHz, CDCl_3_) δ 167.3, 157.3, 140.2, 135.3, 130.2, 129.5, 129.1, 128.5, 126.2, 122.2, 111.1, 55.7, 52.3, 36.1.

##### Methyl 4-Benzyl-3-hydroxybenzoate (6)

To a cooled (0 °C) solution of **5** (500 mg, 1.95 mmol) in DCM (5.0 mL) was added dropwise a 1.0 M BBr_3_ in DCM solution (5.85 mL, 5.85 mmol). The resulting mixture was stirred for 1 h at 0 °C and 2 h at room temperature. The reaction mixture was added dropwise to MeOH and the resulting mixture was stirred for 10 min. A 1.0 M aqueous HCl solution (50 mL) was added. The mixture was extracted with DCM (3 × 40 mL). The combined organic phases were dried over Na_2_SO_4_, filtered and evaporated in vacuo. Purification by flash column chromatography (*n*-heptane/EtOAc 1:0 to 1:1) gave the title compound (329 mg, 70%). ^1^H NMR (500 MHz, DMSO-*d*_6_) δ 10.42–9.42 (br, 1H), 7.42 (d, *J* = 1.7 Hz, 1H), 7.33 (dd, *J* = 7.8, 1.7 Hz, 1H), 7.29–7.24 (m, 2H), 7.24–7.20 (m, 2H), 7.19–7.13 (m, 2H), 3.91 (s, 2H), 3.80 (s, 3H). ^13^C NMR (126 MHz, DMSO-*d*_6_) δ 166.2, 155.2, 140.4, 133.3, 130.5, 128.8, 128.6, 128.3, 125.9, 119.9, 115.3, 52.0, 35.2.

##### Methyl 4-Benzyl-3-((1-methylpiperidin-4-yl)oxy)benzoate (7)

To a solution of phenol **6** (300 mg, 1.24 mmol) and 1-methylpiperidin-4-ol (214 mg, 1.86 mmol) in THF (7.0 mL) was added PPh_3_ (487 mg, 1.86 mmol). The resulting mixture was stirred for 5 min at 0 °C. Subsequently, 40% DEAD in toluene (0.846 mL, 1.86 mmol) was added dropwise and the mixture was stirred for 24 h at room temperature. The reaction mixture was evaporated in vacuo. Purification by flash column chromatography (EtOAc/MeOH/TEA 100:0:0 to 90:5:5) gave the title compound (129 mg, 31%). ^1^H NMR (500 MHz, DMSO-*d*_6_) δ 7.49 (dd, *J* = 7.8, 1.5 Hz, 1H), 7.44 (d, *J* = 1.4 Hz, 1H), 7.33 (d, *J* = 7.8 Hz, 1H), 7.29–7.24 (m, 2H), 7.22–7.18 (m, 2H), 7.18–7.14 (m, 1H), 4.54–4.42 (m, 1H), 3.95 (s, 2H), 3.82 (s, 3H), 2.47–2.29 (m, 2H), 2.25–2.16 (m, 2H), 2.12 (s, 3H), 1.90–1.79 (m, 2H), 1.67–1.53 (m, 2H). ^13^C NMR (126 MHz, DMSO-*d*_6_) δ 166.1, 154.6, 140.2, 135.9, 130.9, 129.0, 128.7, 128.3, 126.0, 121.3, 112.8, 71.4, 52.2, 51.8, 45.9, 35.8, 30.2.

##### (4-Benzyl-3-methoxyphenyl)methanol (8)

Ester **5** (3.00 g, 11.7 mmol) was dissolved in THF (60 mL) and the mixture was cooled to −78 °C. Subsequently, a 1.0 M LAH in THF solution (23.4 mL, 23.4 mmol) was added dropwise. The resulting mixture was allowed to warm up to room temperature and stirred for 4 h. The reaction mixture was quenched with ice-cold water, and the aqueous phase was acidified using 4 M aqueous HCl to pH 1, after which extraction was performed with EtOAc (3 × 40 mL). The combined organic phases were dried over Na_2_SO_4_, filtered and reduced in vacuo to afford the title compound as a slightly yellow solid (2.56 g, 96%). ^1^H NMR (500 MHz, DMSO-*d*_6_) δ 7.28–7.21 (m, 2H), 7.19–7.12 (m, 3H), 7.04 (d, *J* = 7.6 Hz, 1H), 6.93 (s, 1H), 6.82–6.78 (m, 1H), 5.27–5.03 (br, 1H, D_2_O exchangeable), 4.45 (s, 2H), 3.86 (s, 2H), 3.76 (s, 3H). ^13^C NMR (126 MHz, DMSO-*d*_6_) δ 156.7, 142.3, 141.1, 129.8, 128.6, 128.2, 127.2, 125.7, 118.3, 109.0, 62.9, 55.3, 35.0.

##### 4-Benzyl-3-methoxybenzaldehyde (9)

MnO_2_ (4.87 g, 56.1 mmol) was added to a stirred solution of benzyl alcohol **8** (2.56 g, 11.2 mmol) in DCM (120 mL). The resulting mixture was stirred for 3 days at room temperature. Subsequently, the mixture was filtered over 5 × 0.2 μM filters. Water was added and the phases were separated. The aqueous phase was extracted with DCM (2 × 20 mL). The combined organic phases were dried over Na_2_SO_4_, filtered and evaporated in vacuo and gave the title compound as a slightly yellow oil (2.36 g, 93%). ^1^H NMR (500 MHz, DMSO-*d*_6_) δ 9.94 (s, 1H), 7.48 (dd, *J* = 7.5, 1.5 Hz, 1H), 7.45 (d, *J* = 1.4 Hz, 1H), 7.35 (d, *J* = 7.6 Hz, 1H), 7.30–7.25 (m, 2H), 7.24–7.16 (m, 3H), 3.98 (s, 2H), 3.87 (s, 3H). ^13^C NMR (126 MHz, DMSO-*d*_6_) δ 192.6, 157.4, 139.8, 136.6, 136.1, 130.7, 128.8, 128.4, 126.1, 123.3, 109.7, 55.7, 35.4.

##### 4-Benzyl-3-hydroxybenzaldehyde (10)

Sodium ethanethiolate (2.91 g, 31.2 mmol) was added to a solution of **9** (2.35 g, 10.4 mmol) in DMF (75 mL) and the resulting mixture was stirred for 3 h at 130 °C. The mixture was cooled to room temperature, diluted with water (500 mL) and extracted with EtOAc (3 × 75 mL). The combined organic phases were dried over Na_2_SO_4_, filtered and evaporated *in vacuo*. Purification by flash column chromatography (*n*-heptane/EtOAc 95:5 to 70:30) gave the title compound (1.35 g, 61%). ^1^H NMR (500 MHz, DMSO-*d*_6_) δ 10.46–9.87 (br, 1H), 9.85 (s, 1H), 7.32–7.20 (m, 7H), 7.20–7.14 (m, 1H), 3.94 (s, 2H). ^13^C NMR (126 MHz, DMSO-*d*_6_) δ 192.7, 155.7, 140.2, 135.8, 135.2, 130.9, 128.8, 128.3, 126.0, 121.8, 113.8, 35.5.

##### *tert*-Butyl 4-(2-Benzyl-5-formylphenoxy)piperidine-1-carboxylate (11)

Phenol **10** (1.34 g, 6.31 mmol) was dissolved in DMF (25 mL). Subsequently, *tert*-butyl 4-((methylsulfonyl)oxy)piperidine-1-carboxylate^19^ (7.05 g, 25.3 mmol) and Cs_2_CO_3_ (5.14 g, 15.8 mmol) were added and the resulting mixture was stirred for 16 h at 60 °C. The mixture was cooled to room temperature, diluted with water (400 mL) and brine (100 mL), and extracted with EtOAc (4 × 50 mL). The combined organic phases were dried over Na_2_SO_4_, filtered and evaporated in vacuo. Purification by flash column chromatography (*n*-heptane/EtOAc 9:1 to 8:2) gave the title compound as a colorless oil (1.85 g, 74%). ^1^H NMR (500 MHz, DMSO-*d*_6_) δ 9.93 (s, 1H), 7.50–7.41 (m, 3H), 7.27–7.21 (m, 2H), 7.21–7.12 (m, 3H), 4.76–4.65 (m, 1H), 3.98 (s, 2H), 3.45–3.37 (m, 2H), 3.28–3.17 (m, 2H), 1.87–1.77 (m, 2H), 1.56–1.44 (m, 2H), 1.40 (s, 9H). ^13^C NMR (126 MHz, DMSO-*d*_6_) δ 192.7, 155.0, 153.9, 140.1, 137.4, 136.1, 131.4, 128.7, 128.3, 126.0, 122.8, 112.0, 78.7, 71.5, 39.9 (confirmed by HSQC, overlaps with solvent peak), 36.0, 29.9, 28.1.

##### *tert*-Butyl 4-(2-Benzyl-5-(2-methoxyvinyl)phenoxy)piperidine-1-carboxylate (12)

To a mixture of (methoxymethyl)triphenylphosphonium chloride (7.80 g, 22.8 mmol) in THF (100 mL) at 0 °C was added potassium *tert*-butoxide (2.55 g, 22.8 mmol). The resulting mixture was stirred for 25 min at room temperature. A solution of aldehyde **11** (3.00 g, 7.59 mmol) in THF (25 mL) was added and the mixture was stirred overnight at room temperature. The reaction mixture was diluted with water (1.0 L) and extracted with DCM (3 × 300 mL). The combined organic phases were dried over Na_2_SO_4_, filtered and evaporated in vacuo. Purification by flash column chromatography (*n*-heptane/EtOAc 6:4) gave enol ether **12** as a colorless oil as an inseparable mixture of *E*/*Z* isomers (2.11 g, 66%). The following ^1^H NMR listing describes the peaks of both isomers: ^1^H NMR (500 MHz, DMSO-*d*_6_) δ 7.29–7.19 (m, 2.5H), 7.19–7.10 (m, 3.5H), 7.09–7.01 (m, 1.5H), 6.93–6.91 (m, 0.5H), 6.78 (dd, *J* = 7.7, 1.3 Hz, 0.5H), 6.25 (d, *J* = 7.0 Hz, 0.5H), 5.78 (d, *J* = 13.0 Hz, 0.5H), 5.18 (d, *J* = 7.1 Hz, 0.5H), 4.62–4.56 (m, 0.5H), 4.51–4.45 (m, 0.5H), 3.83 (m, 2H), 3.73 (s, 1.5H), 3.61 (s, 1.5H), 3.46–3.36 (m, 2H), 3.25–3.15 (m, 2H), 1.83–1.75 (m, 2H), 1.53–1.35 (m, 11H).

##### 4-(2-Benzyl-5-(2-methoxyvinyl)phenoxy)-1-methylpiperidine (13)

To a cooled (0 °C) solution of **12** (2.00 g, 4.72 mmol) in THF (100 mL) was added a 1.0 M LAH in THF solution (9.44 mL, 9.44 mmol). The resulting mixture was stirred for 30 min at 0 °C and subsequently 8 h at 45 °C. The reaction mixture was cooled to room temperature, quenched with 1.0 M aqueous NaOH (100 mL) and extracted with DCM (3 × 50 mL). The combined organic phases were dried over Na_2_SO_4_, filtered, evaporated in vacuo and coevaporated in vacuo with DCM twice to afford tertiary amine **13** as an inseparable mixture of *E*/*Z* isomers (1.46 g, 92%). The following ^1^H NMR listing describes the peaks of both isomers: ^1^H NMR (500 MHz, CDCl_3_) δ 7.26–6.97 (m, 7.45H), 6.76 (dd, *J* = 7.8, 1.6 Hz, 0.55H), 6.70–6.67 (m, 0.55H), 6.10 (d, *J* = 7.0 Hz, 0.45H), 5.76 (d, *J* = 12.9 Hz, 0.55H), 5.18 (d, *J* = 7.0 Hz, 0.45H), 4.45–4.31 (br, 1H), 3.95 (s, 0.9H), 3.93 (s, 1.1H), 3.76 (s, 1.35H), 3.67 (s, 1.65H), 2.58–2.40 (br, 2H), 2.38–2.21 (m, 5H), 2.00–1.92 (m, 2H), 1.87–1.77 (m, 2H).

##### 4-Benzyl-3-((1-methylpiperidin-4-yl)oxy)benzaldehyde (15)

MnO_2_ (1.82 g, 20.9 mmol) was added to a stirred solution of the free base of benzyl alcohol **2d** (1.30 g, 4.17 mmol) in DCM (40 mL). The resulting mixture was stirred for 16 h at room temperature. Subsequently, the mixture was filtered over 5 × 0.2 μM filters to afford the crude product (750 mg, which had sufficient purity for the next step. ^1^H NMR (400 MHz, CDCl_3_) δ 9.92 (s, 1H), 7.40–7.26 (m, 5H, overlaps with solvent peak), 7.23–7.15 (m, 3H), 4.62–4.39 (m, 1H), 4.04 (s, 2H), 2.61–2.34 (m, 4H), 2.28 (s, 3H), 2.13–1.98 (m, 2H), 1.97–1.80 (m, 2H).

##### 2-Benzyl-5-methoxyphenol (17)

To a solution of ketone **16** (10.00 g, 43.8 mmol) in DCM (30 mL) was added TFA (33.8 mL, 438 mmol) dropwise. Next, Et_3_SiH (27.8 mL, 175 mmol) was added and the resulting mixture was stirred for 17 h at room temperature. A saturated aqueous NH_4_Cl solution (500 mL) was added and the layers were separated. The aqueous phase was extracted with DCM (2 × 100 mL). The combined organic phases were washed with brine, dried over Na_2_SO_4_, filtered and evaporated in vacuo to give the title compound as a slightly yellow solid (4.85 g, 52%). ^1^H NMR (500 MHz, DMSO-*d*_6_) δ 9.52–9.32 (br, 1H), 7.26–7.20 (m, 2H), 7.20–7.15 (m, 2H), 7.16–7.09 (m, 1H), 6.91 (d, *J* = 8.3 Hz, 1H), 6.38 (d, *J* = 2.5 Hz, 1H), 6.30 (dd, *J* = 8.3, 2.5 Hz, 1H), 3.77 (s, 2H), 3.65 (s, 3H). ^13^C NMR (126 MHz, DMSO-*d*_6_) δ 158.6, 155.8, 141.8, 130.7, 128.5, 128.1, 125.5, 119.8, 104.2, 101.2, 54.8, 34.6.

##### 3-Benzyl-2-hydroxybenzaldehyde (19)

Anhydrous magnesium chloride beads (4.52 g, 47.4 mmol) and *para*-formaldehyde (2.14 g, 71.2 mmol) were added to an oven-dried reaction flask followed by THF (125 mL). Subsequently, TEA (6.61 mL, 47.4 mmol) was added dropwise, followed by 2-benzylphenol (**18**) (4.37 g, 23.7 mmol) dissolved in THF (12 mL). The resulting mixture was stirred at reflux for 4 h. The reaction mixture was cooled to room temperature, diluted with diethyl ether (100 mL) and washed with 1.0 M aqueous HCl (3 × 100 mL) and water (3 × 100 mL). The organic phase was dried over Na_2_SO_4_, filtered and evaporated in vacuo to obtain a yellow oil. The yellow oil was dissolved in EtOAc/*n*-heptane (1:1) and upon cooling (−78 °C) a precipitate was formed, which was filtered and dried in vacuo*.* This afforded the title compound as a slightly yellow solid (3.33 g, 66%). ^1^H NMR (500 MHz, DMSO-*d*_6_) δ 11.18 (s, 1H), 10.02 (s, 1H), 7.65 (dd, *J* = 7.6, 1.7 Hz, 1H), 7.48 (dd, *J* = 7.6, 1.7 Hz, 1H), 7.30–7.21 (m, 4H), 7.20–7.15 (m, 1H), 7.02 (t, *J* = 7.6 Hz, 1H), 3.96 (s, 2H). ^13^C NMR (126 MHz, DMSO-*d*_6_) δ 197.3, 158.3, 140.1, 137.5, 131.8, 129.4, 128.6, 128.4, 126.0, 120.9, 119.8, 34.2.

##### *tert*-Butyl 4-(2-benzyl-6-formylphenoxy)piperidine-1-carboxylate (20)

Aldehyde **19** (2.00 g, 9.42 mmol) was dissolved in DMF (25 mL). Subsequently, Cs_2_CO_3_ (9.21 g, 28.3 mmol) and *tert*-butyl 4-((methylsulfonyl)oxy)piperidine-1-carboxylate^19^ (6.58 g, 23.6 mmol) were added and the resulting mixture was stirred for 32 h at 65 °C. The mixture was cooled to room temperature, diluted with water (500 mL) and extracted with EtOAc (3 × 150 mL). The combined organic phases were washed with saturated aqueous NaHCO_3_ (150 mL), brine (150 mL), dried over Na_2_SO_4_, filtered and evaporated in vacuo. Purification by flash column chromatography (*n*-heptane/EtOAc 9:1 to 8:2) gave the title compound as a white solid (2.60 g, 70%). ^1^H NMR (500 MHz, DMSO-*d*_6_) δ 10.27 (s, 1H), 7.64 (dd, *J* = 7.7, 1.7 Hz, 1H), 7.47 (dd, *J* = 7.5, 1.7 Hz, 1H), 7.33–7.15 (m, 6H), 4.14–4.03 (m, 3H), 3.96–3.83 (m, 2H), 2.91–2.65 (br, 2H), 1.98–1.88 (m, 2H), 1.64–1.50 (m, 2H), 1.39 (s, 9H). ^13^C NMR (126 MHz, DMSO-*d*_6_) δ 190.4, 157.7, 153.7, 140.2, 137.2, 136.1, 129.8, 128.8, 128.5, 126.8, 126.2, 124.4, 82.4, 78.8, 40.9 (confirmed by HSQC), 35.0, 31.3, 28.1.

##### 3-Benzyl-2-((1-methylpiperidin-4-yl)oxy)benzaldehyde (21)

MnO_2_ (2.37 g, 27.3 mmol) was added to a stirred solution of benzyl alcohol **3d** (1.70 g, 5.46 mmol) in DCM (60 mL). The resulting mixture was stirred for 2 days at room temperature. Subsequently, the mixture was filtered over Celite and the solvent was evaporated in vacuo. Purification by column chromatography (EtOAc/(EtOAc/TEA/MeOH) 100:0 to 30:70(90:5:5)) gave the title compound as a yellowish solid (1.35 g, 80%). ^1^H NMR (500 MHz, DMSO-*d*_6_) δ 10.28 (s, 1H), 7.63 (dd, *J* = 1.8, 7.7 Hz, 1H), 7.47 (dd, *J* = 1.6, 7.5 Hz, 1H), 7.33–7.26 (m, 2H), 7.25–7.16 (m, 4H), 4.05 (s, 2H), 3.91–3.83 (m, 1H), 2.74–2.66 (m, 2H), 2.12 (s, 3H), 1.93–1.81 (m, 4H), 1.79–1.68 (m, 2H). ^13^C NMR (126 MHz, DMSO-*d*_6_) δ 190.3, 157.9, 140.2, 137.2, 135.9, 129.9, 128.6, 128.4, 126.6, 126.1, 124.2, 82.3, 53.1, 45.5, 35.0, 31.3.

##### 4-(2-Benzyl-6-(2-methoxyvinyl)phenoxy)-1-methylpiperidine (22)

Potassium *tert-*butoxide (326 mg, 2.91 mmol) was added to a solution of (methoxymethyl)triphenylphosphonium chloride (997 mg, 2.91 mmol) in THF (10 mL). The mixture was stirred for 15 min at room temperature. A solution of aldehyde **21** (300 mg, 0.97 mmol) was added and the resulting mixture was stirred for 16 h at room temperature. The reaction mixture was diluted with water (50 mL) and saturated aqueous NaHCO_3_ (50 mL). Extraction was performed with EtOAc (3 × 50 mL). The combined organic phases were dried over Na_2_SO_4_, filtered and evaporated in vacuo. Purification by flash column chromatography (DCM/MeOH 10:0 to 9:1) gave enol ether **22** as a colorless oil as an inseparable mixture of *E*/*Z* isomers (240 mg, 73%). The following ^1^H NMR listing describes the peaks of both isomers: ^1^H NMR (500 MHz, DMSO-*d*_6_) δ 7.78 (dd, *J* = 7.7, 1.8 Hz, 0.4H), 7.30–7.12 (m, 6.2H), 6.98–6.83 (m, 2H), 6.35 (d, *J* = 7.2 Hz, 0.4H), 5.93 (d, *J* = 13.1 Hz, 0.6H), 5.41 (d, *J* = 7.3 Hz, 0.4H), 3.95 (s, 2H), 3.82–3.72 (m, 2.2H), 3.64 (s, 1.8H), 2.74–2.65 (m, 2H), 2.15–2.09 (m, 3H), 1.90–1.79 (m, 4H), 1.79–1.65 (m, 2H).

##### Ethyl 3-(4-(2-Benzylphenoxy)piperidin-1-yl)propanoate (25a)

A mixture of **24**(19) (270 mg, 1.01 mmol), ethyl 3-bromopropanoate (0.353 mL, 2.75 mmol) and K_2_CO_3_ (391 mg, 2.83 mmol) in DMF (7.5 mL) was stirred at 80 °C for 9 h. The resulting mixture was concentrated in vacuo and subsequently diluted with water (100 mL) and extracted with EtOAc (3 × 50 mL). The combined organic phases were washed with brine, dried over Na_2_SO_4_, filtered and evaporated in vacuo. Purification by flash chromatography (DCM/(EtOAc/TEA) 100:0 to 75:25(99:1)) gave the title compound as a colorless oil (215 mg, 58%). ^1^H NMR (400 MHz, CD_3_OD) δ 7.25–7.09 (m, 7H), 6.91 (d, *J* = 8.1 Hz, 1H), 6.86 (t, *J* = 7.4 Hz, 1H), 4.49–4.38 (m, 1H), 4.14 (q, *J* = 7.1 Hz, 2H), 3.95 (s, 2H), 2.62 (t, *J* = 7.4 Hz, 2H), 2.57–2.44 (m, 4H), 2.42–2.31 (m, 2H), 1.96–1.83 (m, 2H), 1.79–1.68 (m, 2H), 1.26 (t, *J* = 7.1 Hz, 3H). ^13^C NMR (101 MHz, CD_3_OD) δ 173.9, 156.3, 142.9, 132.1, 131.4, 129.7, 129.2, 128.6, 126.7, 121.4, 113.7, 72.0, 61.6, 54.5, 50.7, 37.4, 32.8, 31.2, 14.5.

##### Ethyl 4-(4-(2-Benzylphenoxy)piperidin-1-yl)butanoate (25b)

This compound was synthesized according to the procedure for **25a** using **24**(19) (300 mg, 1.12 mmol), ethyl 4-bromobutanoate (0.181 mL, 1.26 mmol), K_2_CO_3_ (174 mg, 1.26 mmol), DMF (7.5 mL) and a heating time of 7.5 h at 80 °C. Purification by flash chromatography (DCM/(EtOAc/TEA) 100:0 to 75:25(99:1)) gave the title compound as a colorless oil (135 mg, 32%). ^1^H NMR (400 MHz, CD_3_OD) δ 7.28–7.10 (m, 7H), 6.97–6.87 (m, 2H), 4.59–4.51 (m, 1H), 4.15 (q, *J* = 7.1 Hz, 2H), 3.98 (s, 2H), 2.78–2.49 (m, 6H), 2.38 (t, *J* = 7.1 Hz, 2H), 2.02–1.79 (m, 6H), 1.26 (t, *J* = 7.1 Hz, 3H). ^13^C NMR (101 MHz, CD_3_OD) δ 174.5, 156.0, 143.1, 132.5, 131.1, 129.6, 129.4, 128.8, 126.9, 121.7, 113.5, 69.2 (confirmed by HSQC), 61.7, 58.1, 50.4, 37.6, 32.4, 30.0, 21.9, 14.5.

##### Ethyl 5-(4-(2-Benzylphenoxy)piperidin-1-yl)pentanoate (25c)

This compound was synthesized according to the procedure of **25a** using **24**(19) (200 mg, 0.75 mmol), ethyl 5-bromopentanoate (0.133 mL, 0.84 mmol), K_2_CO_3_ (116 mg, 0.84 mmol), DMF (8 mL) and a heating time of 3.5 h at 80 °C. Purification by flash chromatography (DCM/(EtOAc/TEA) 100:0 to 0:100(99:1)) gave the title compound as a colorless oil (209 mg, 71%). ^1^H NMR (500 MHz, CD_3_OD) δ 7.24–7.09 (m, 7H), 6.92 (d, *J* = 8.1 Hz, 1H), 6.89–6.83 (m, 1H), 4.48–4.42 (m, 1H), 4.12 (q, *J* = 7.1 Hz, 2H), 3.95 (s, 2H), 2.57–2.44 (m, 2H), 2.41–2.28 (m, 6H), 1.96–1.88 (m, 2H), 1.80–1.71 (m, 2H), 1.64–1.56 (m, 2H), 1.56–1.46 (m, 2H), 1.25 (t, *J* = 7.1 Hz, 3H). ^13^C NMR (126 MHz, CDCl_3_) δ 173.8, 155.2, 141.4, 131.0, 130.6, 129.0, 128.3, 127.4, 125.9, 120.4, 112.6, 71.9 (confirmed by HSQC), 60.4, 58.3, 50.5, 36.5, 34.3, 30.8, 26.7, 23.2, 14.4.

##### Ethyl 6-(4-(2-Benzylphenoxy)piperidin-1-yl)hexanoate (25d)

The compound was synthesized according to the procedure of **25a** using **24** (207 mg, 0.77 mmol), ethyl 6-bromohexanoate (0.155 mL, 0.87 mmol), K_2_CO_3_ (120 mg, 0.87 mmol), DMF (8 mL) and a heating time of 3 h at 80 °C. Purification by flash chromatography (DCM/(EtOAc/TEA) (100:0 to 0:100(99:1)) gave the title compound as a colorless oil (211 mg, 67%). ^1^H NMR (500 MHz, CD_3_OD) δ 7.24–7.09 (m, 7H), 6.92 (d, *J* = 8.0 Hz, 1H), 6.88–6.84 (m, 1H), 4.49–4.41 (m, 1H), 4.12 (q, *J* = 7.1 Hz, 2H), 3.95 (s, 2H), 2.57–2.26 (m, 8H), 1.97–1.87 (m, 2H), 1.81–1.71 (m, 2H), 1.68–1.60 (m, 2H), 1.53–1.45 (m, 2H), 1.38–1.28 (m, 2H), 1.25 (t, *J* = 7.1 Hz, 3H). ^13^C NMR (126 MHz, CD_3_OD) δ 175.4, 156.3, 142.9, 132.1, 131.3, 129.7, 129.2, 128.6, 126.7, 121.4, 113.7, 71.5 (confirmed by HSQC), 61.4, 59.5, 50.9, 37.4, 35.0, 31.1, 28.2, 27.2, 25.9, 14.6.

##### Ethyl 7-(4-(2-Benzylphenoxy)piperidin-1-yl)heptanoate (25e)

This compound was synthesized according to the procedure of **25a** using **24**(19) (203 mg, 0.76 mmol), ethyl 7-bromoheptanoate (0.166 mL, 0.85 mmol), K_2_CO_3_ (118 mg, 0.85 mmol), DMF (8 mL) and a heating time of 3 h at 80 °C. Purification by flash chromatography (DCM/(EtOAc/TEA) 100:0 to 0:100(99:1)) gave the title compound as a colorless oil (225 mg, 70%). ^1^H NMR (500 MHz, CD_3_OD) δ 7.24–7.09 (m, 7H), 6.92 (d, *J* = 8.1 Hz, 1H), 6.86 (td, *J* = 7.4, 1.0 Hz, 1H), 4.49–4.41 (m, 1H), 4.11 (q, *J* = 7.1 Hz, 2H), 3.95 (s, 2H), 2.57–2.25 (m, 8H), 1.96–1.87 (m, 2H), 1.81–1.71 (m, 2H), 1.66–1.58 (m, 2H), 1.53–1.44 (m, 2H), 1.40–1.29 (m, 4H), 1.24 (t, *J* = 7.1 Hz, 3H). ^13^C NMR (126 MHz, CD_3_OD) δ 175.6, 156.3, 142.9, 132.1, 131.4, 129.7, 129.2, 128.6, 126.7, 121.4, 113.7, 71.1 (confirmed by HSQC), 61.4, 59.6, 50.9, 37.4, 35.0, 31.1, 30.0, 28.4, 27.3, 26.0, 14.5.

##### Ethyl 8-(4-(2-Benzylphenoxy)piperidin-1-yl)octanoate (25f)

This compound was synthesized according to the procedure of **25a** using **24**(19) (265 mg, 0.99 mmol), ethyl 8-bromooctanoate (0.291 mL, 1.33 mmol), K_2_CO_3_ (184 mg, 1.33 mmol), DMF (7.5 mL) and a heating time of 3 h at 80 °C. Purification by flash chromatography (DCM/(EtOAc/TEA) 100:0 to 25:75(99:1)) gave the title compound as a colorless oil (220 mg, 51%). ^1^H NMR (400 MHz, CD_3_OD) δ 7.25–7.09 (m, 7H), 6.92 (d, *J* = 8.1 Hz, 1H), 6.86 (t, *J* = 7.4 Hz, 1H), 4.49–4.39 (m, 1H), 4.11 (q, *J* = 7.1 Hz, 2H), 3.96 (s, 2H), 2.63–2.34 (m, 4H), 2.34–2.24 (m, 4H), 1.97–1.86 (m, 2H), 1.82–1.71 (m, 2H), 1.67–1.57 (m, 2H), 1.53–1.44 (m, 2H), 1.41–1.29 (m, 6H), 1.24 (t, *J* = 7.1 Hz, 3H). ^13^C NMR (101 MHz, CD_3_OD) δ 175.6, 156.3, 142.9, 132.2, 131.3, 129.7, 129.2, 128.6, 126.7, 121.4, 113.7, 71.5 (confirmed by HSQC), 61.4, 59.7, 50.9, 37.4, 35.1, 31.0, 30.2, 30.1, 28.5, 27.4, 26.0, 14.5.

##### 5-(4-(2-Benzylphenoxy)piperidin-1-yl)pentanenitrile Fumarate (26)

A microwave vial was charged with amine **24**(19) (2.00 g, 7.48 mmol), K_2_CO_3_ (1.16 g, 8.42 mmol), 5-bromopentanenitrile (0.95 mL, 8.2 mmol) and DMF (50 mL). The resulting mixture was stirred for 2 h at 85 °C. The reaction mixture was concentrated in vacuo, diluted with water (1.0 L) and extracted with EtOAc (300 mL). The organic phase was washed with brine (100 mL), dried over Na_2_SO_4_, filtered and concentrated in vacuo. Purification by flash column chromatography ((EtOAc:TEA):(EtOAc:TEA:MeOH) (99:1):0 to 0:(90:5:5) gave the free base (1.27 g, 49%). Part of the free base (120 mg, 0.34 mmol) was converted to a fumaric acid salt, with the method described by Kuhne et al.,^19^ to obtain the title compound as a white solid (45 mg, 28%). ^1^H NMR (500 MHz, DMSO-*d*_6_) δ 7.28–7.22 (m, 2H), 7.21–7.12 (m, 5H), 6.98 (d, *J* = 8.1 Hz, 1H), 6.88–6.83 (m, 1H), 6.57 (s, 2H), 4.51–4.44 (bs, 1H), 3.90 (s, 2H), 2.71–2.55 (m, 2H), 2.54–2.51 (m, 2H), 2.48–2.41 (m, 4H), 1.97–1.86 (m, 2H), 1.73–1.63 (m, 2H), 1.60–1.50 (m, 4H). ^13^C NMR (126 MHz, DMSO-*d*_6_) δ 166.7, 154.4, 141.2, 134.4, 130.7, 129.9, 128.6, 128.2, 127.5, 125.7, 120.7, 120.3, 112.8, 56.0, 49.2, 35.8, 29.4, 24.6, 22.7, 16.0 (no ^13^C resonance was observed for the O*C*H moiety, but a HSQC signal was observed at 4.51–4.44 ppm (^1^H) and 69.9 ppm (^13^C)). HRMS: C_23_H_29_N_2_O^+^ [M + H]^+^ calcd: 349.2274, found 349.2271.

##### 1-(4-(1*H*-Tetrazol-5-yl)butyl)-4-(2-benzylphenoxy)piperidine (27)

A mixture of nitrile **26** (0.385 g, 1.11 mmol), NH_4_Cl (0.355 g, 6.63 mmol), NaN_3_ (0.431 g, 6.63 mmol) and DMF (15 mL) was stirred for 3 d at 100 °C. The reaction mixture was diluted with water (50 mL) and extracted with DCM (3 × 50 mL). The combined organic phases were dried over Na_2_SO_4_, filtered and concentrated in vacuo. Purification by reversed phase column chromatography (H_2_O:MeCN 95:5 to 20:80) gave the title compound as a white solid (120 mg, 28%). ^1^H NMR (500 MHz, DMSO-*d*_6_) δ 7.27–7.22 (m, 2H), 7.22–7.11 (m, 5H), 6.97 (d, *J* = 8.0 Hz, 1H), 6.85 (t, *J* = 7.4 Hz, 1H), 4.49–4.40 (bs, 1H), 3.89 (s, 2H), 2.85 (t, *J* = 7.4 Hz, 2H), 2.65–2.53 (m, 2H), 2.43–2.30 (m, 4H), 1.95–1.83 (m, 2H), 1.74–1.58 (m, 4H), 1.51–1.42 (m, 2H). ^13^C NMR (126 MHz, DMSO-*d*_6_) δ 156.9, 154.5, 141.1, 130.6, 129.9, 128.6, 128.1, 127.5, 125.7, 120.2, 112.9, 70.8, 56.8, 49.5, 35.7, 29.8, 25.3, 25.1, 23.0. HRMS: C_23_H_30_N_5_O^+^ [M + H]^+^ calcd: 392.2445, found 392.2443.

## Supporting Information Available

LC purities of all compounds and spectral and chromatography data for all final compounds (PDF)
PDB coordinates of all modeled ligand–H1R complexes (ZIP)
Molecular formula strings of all characterized analogues (CSV)

## Abbreviations

Acri: acrivastine
DMEM: Dulbecco’s Modified Eagle Medium
dox: doxepin
FBS: fetal bovine serum H_1_R
HA: non-hydrogen atoms
Histamine: H_1_ receptor
KE: kinetic efficiency
levo: levocetirizine
LE: ligand efficiency
mep: mepyramine
olo: olopatadine
PEI: polyethylenimine
RT: residence time
SKR: structure–kinetic relationship
TEA: trimethylamine
trip: triprolidine
