# Supplementary material for: Probing the Histamine H1 Receptor Binding Site to Explore Ligand Binding Kinetics
Source: J Med Chem. 2024 Dec 26;68(1):448–64. doi: 10.1021/acs.jmedchem.4c02043 (PMC11726634; doi:10.1021/acs.jmedchem.4c02043)
Supplement: Supplementary file 1 — jm4c02043_si_001.pdf [file jm4c02043_si_001.pdf]

SUPPORTING INFORMATION

Probing the Histamine H_1 Receptor Binding Site to Explore Ligand Binding Kinetics

*Sebastiaan Kuhne[†#], Reggie Bosma[†#], Albert J. Kooistra[†], Rick Riemens[†], Marc C.M. Stroet[†], Henry F. Vischer[†], Chris de Graaf[†], Maikel Wijtmans[†], Rob Leurs[†], Iwan J.P. de Esch[†] **

[†]Amsterdam Institute of Molecular and Life Sciences (AIMMS), Division of Medicinal Chemistry, Faculty of Science, Vrije Universiteit Amsterdam, De Boelelaan 1108, 1081 HZ Amsterdam, The Netherlands.

Table of contents

Supporting Figure 1 S3

Supporting Figure 2 S4

LC purities of all compounds S5

Spectral and chromatography data for all final compounds S6-S37

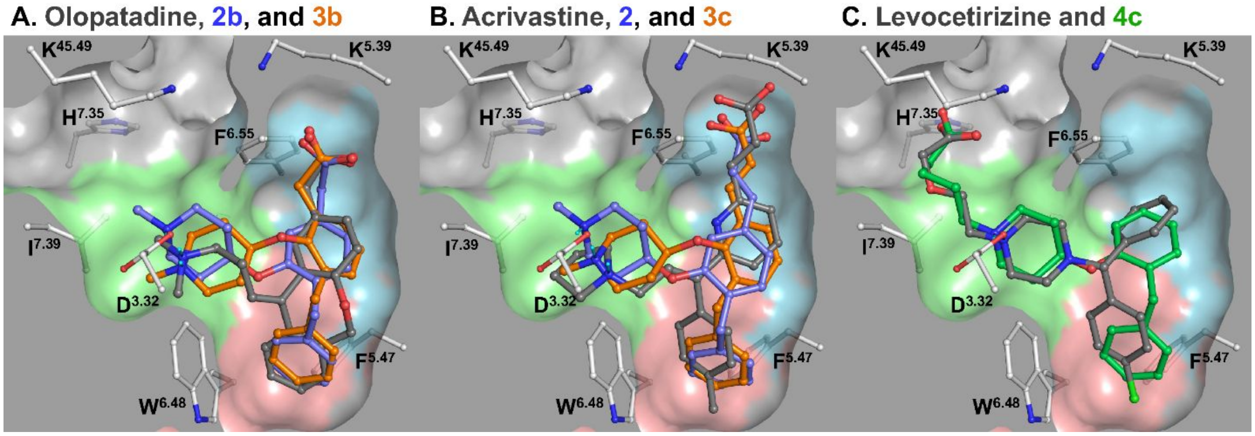

Supporting Figure 1. Proposed binding mode of the H_1R reference compounds together with their respective mimics to H_1R: (A) olopatadine (gray carbon atoms) together with **2b** (blue carbon atoms) and **3b** (orange carbon atoms); (B) acrivatine (gray carbon atoms) together with **2c** (blue carbon atoms) and **3c** (orange carbon atoms); (C) levocetirizine (gray carbon atoms) together with **4c** (green carbon atoms). Binding modes were obtained by docking the compounds in the H_1R crystal structure (pdb: 3RZE). Important binding site residues are represented as ball-and-sticks with light gray carbon atoms. Nitrogen, oxygen, and hydrogen atoms are colored blue, red, and cyan, respectively. Polar hydrogen atoms of the ligands are shown, but are absent for the binding site residues. The H_1R binding site surface is shown and colored to designate the four different regions of the binding site, i.e., the amine binding region, the lower aromatic binding region, the upper aromatic binding region, and the phosphate binding region.

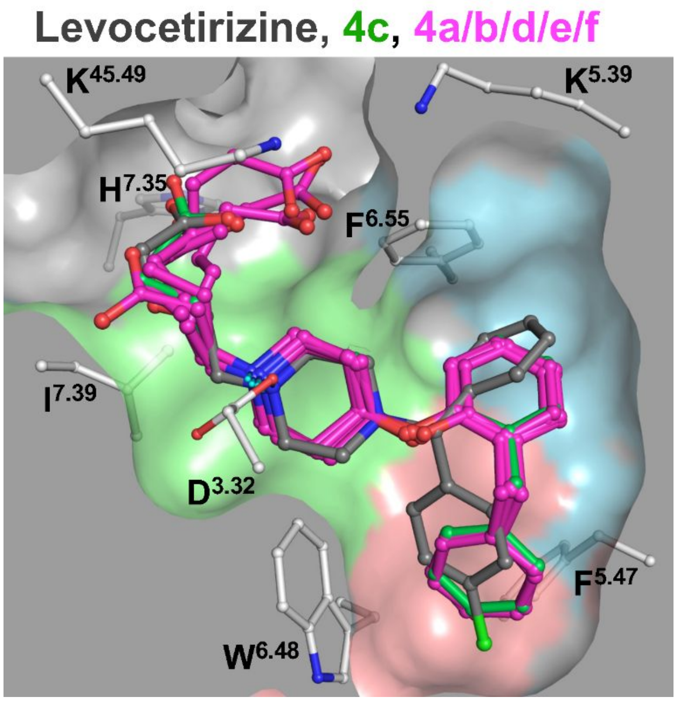

Supporting Figure 2. Proposed binding mode of levocetirizine together with its mimics to H_1R: levocetirizine (gray carbon atoms) together with **4c** (green carbon atoms) and **4a/b/d/e/f** (magenta carbon atoms). Binding modes were obtained by docking the compounds in the H_1R crystal structure (pdb: 3RZE). Important binding site residues are represented as ball-and-sticks with light gray carbon atoms. Nitrogen, oxygen, and hydrogen atoms are colored blue, red, and cyan, respectively. Polar hydrogen atoms of the ligands are shown, but are absent for the binding site residues. The H_1R binding site surface is shown and colored to designate the four different regions of the binding site, i.e., the amine binding region, the lower aromatic binding region, the upper aromatic binding region, and the phosphate binding region.

Table S1. Purities: calculated as the percentage peak area of the analyzed compound by UV detection at 230 nm.

Compound #	VUF#	Purity	t_R (min)	LR-MS $[M+H]^+$
**2a**	VUF15226	99%	3.0	326.15
**2b**	VUF15225	97%	3.0	340.15
**2c**	VUF15288	99%	3.2	352.10
**2d**	VUF15036	98%	3.0	312.15
**2e**	VUF14941	99%	3.6	312.00
**3a**	VUF15347	99%	3.1	326.10
**3b**	VUF15289	99%	3.1	340.15
**3c**	VUF15346	99%	3.3	352.10
**3d**	VUF14940	96%	3.1	312.00
**4a**	VUF14502	99%	3.9	340.05
**4b**	VUF14504	99%	3.8	354.10
**4c**	VUF14506	99%	4.1	368.05
**4d**	VUF14508	99%	4.1	382.10
**4e**	VUF14510	99%	4.3	396.10
**4f**	VUF14511	99%	4.1	410.15
**5**	-	98%	5.3	257.05
**6**	-	99%	4.6	243.10
**7**	-	98%	3.3	340.15
**8**	-	98%	4.5	Not found
**9**	-	99%	5.0	227.05
**10**	-	99%	4.3	213.10
**11**	-	99%	5.6	340.10; 296.15[a]
**12**	-	99%	5.9[b]	368.15; 324.15[a]
**13**	-	99%	3.7[b]	338.20
**15**	-	93%	3.2	310.10
**17**	-	99%	4.5	214.95
**19**	-	98%	5.0	213.04
**20**	-	97%	5.6	396.05; 339.95; 296.00[c]
**21**	-	98%	3.3	310.00
**22**	-	99%	3.5	338.20[b]
**25a**	-	98%	3.9	367.95
**25b**	-	97%	4.3	382.00
**25c**	-	96%	3.9	396.15
**25d**	-	99%	4.1	410.15
**25e**	-	97%	4.1	424.20
**25f**	-	95%	4.2	438.15
**26**	-	99%	4.0	349.05
**27**	VUF14989	99%	3.4	392.05

[a] Mass itself was not found but $[M+H\text{-isobutene}]^+$ and $[M+H\text{-isobutene-}CO_2]^+$ were found. [b] Combined purity of the E/Z-mixture due to overlapping peaks. [c] Small mass peak observed, but also the $[M+H\text{-isobutene}]^+$ and $[M+H\text{-isobutene-}CO_2]^+$ were found.**Compound 2a**

Name: 1H - SKE232
Date & Time: 2015-09-08T10:24:14
Nucleus: 1H
Solvent: DMSO
Number of Scans: 16
Spectrometer Frequency: 500.23 MHz
Temperature: 294.1 K
Pulse Sequence: zg30
Relaxation Time: 1 sec

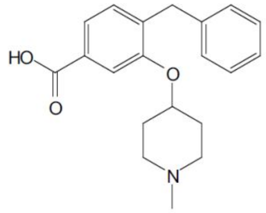

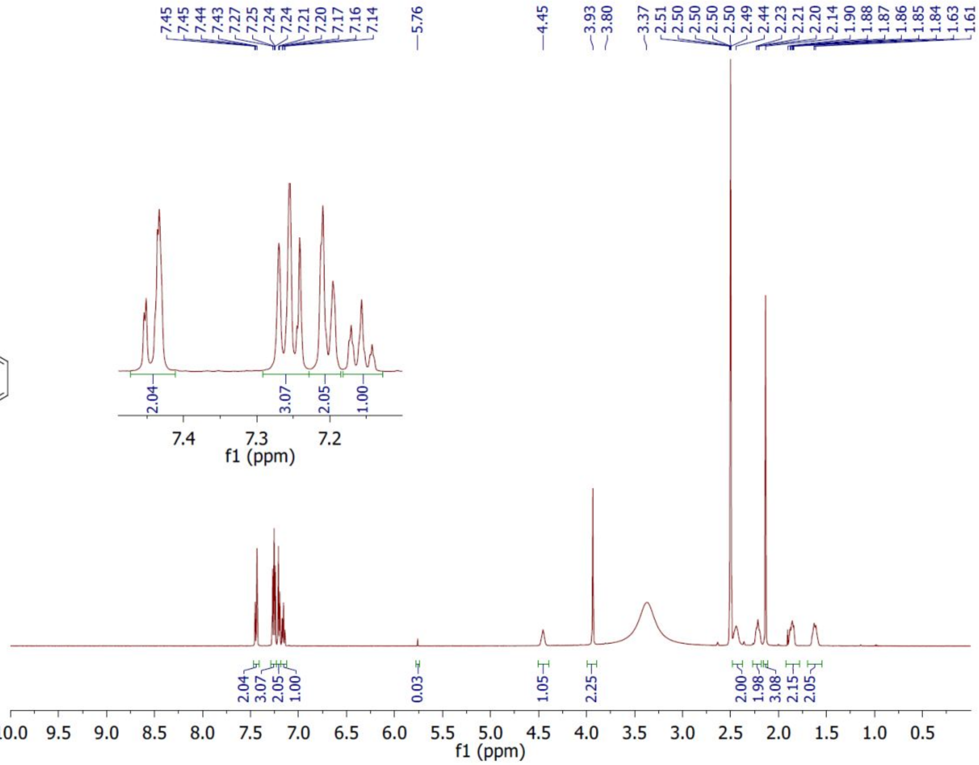

Name: 13C APT - SKE232
Date & Time: 2015-09-09T04:46:53
Nucleus: 13C
Solvent: DMSO
Number of Scans: 12000
Spectrometer Frequency: 125.8 MHz
Temperature: 294.1 K
Pulse Sequence: jmod
Relaxation Time: 2 sec

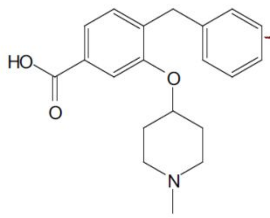

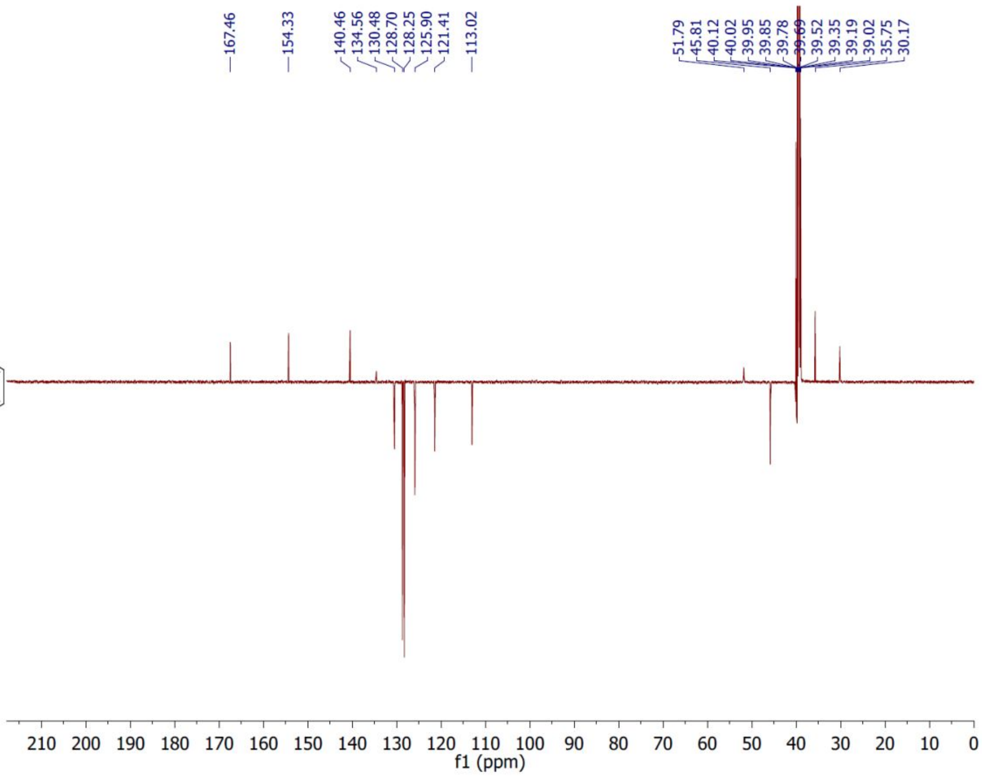

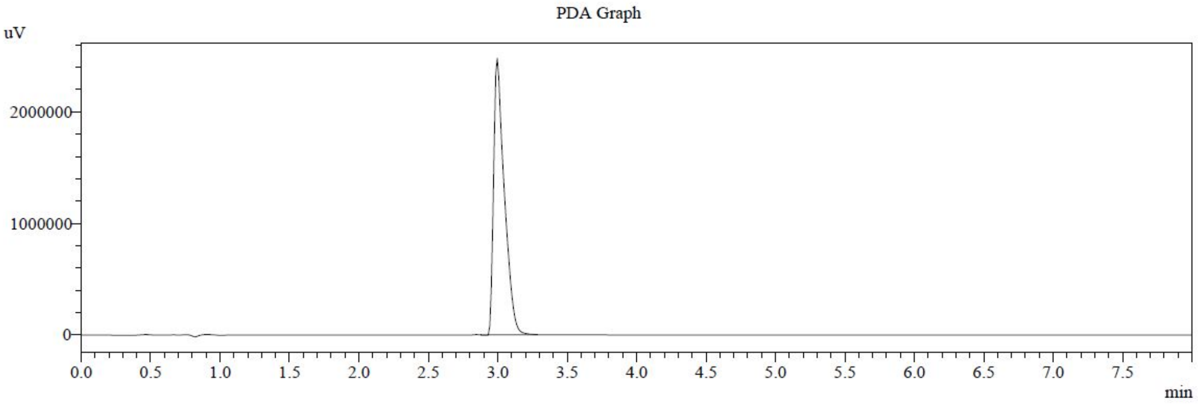

PDA Ch1 230nm 4nm

Peak#	Name	Ret. Time	Area	Area %
1		2.992	13266971	100.000

Compound 2b

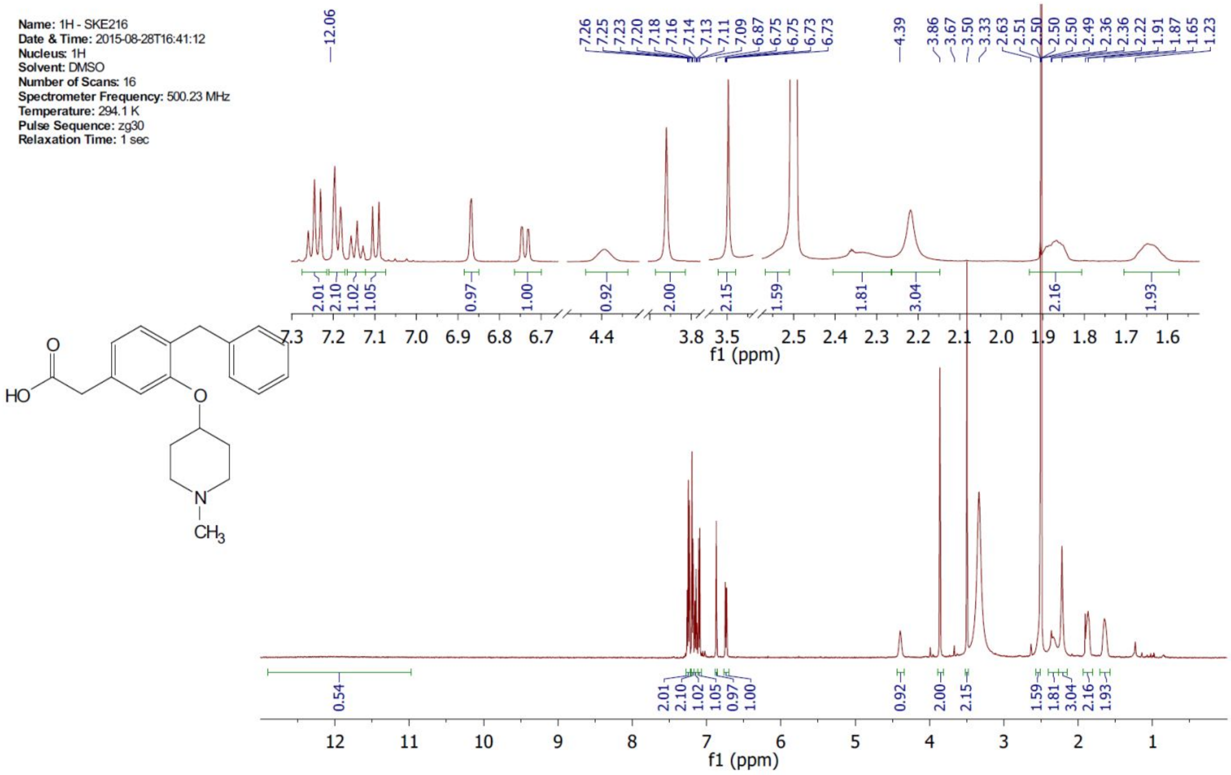
Name: 1H - SKE216
Date & Time: 2015-08-28T16:41:12
Nucleus: 1H
Solvent: DMSO
Number of Scans: 16
Spectrometer Frequency: 500.23 MHz
Temperature: 294.1 K
Pulse Sequence: zg30
Relaxation Time: 1 sec
f1 (ppm)

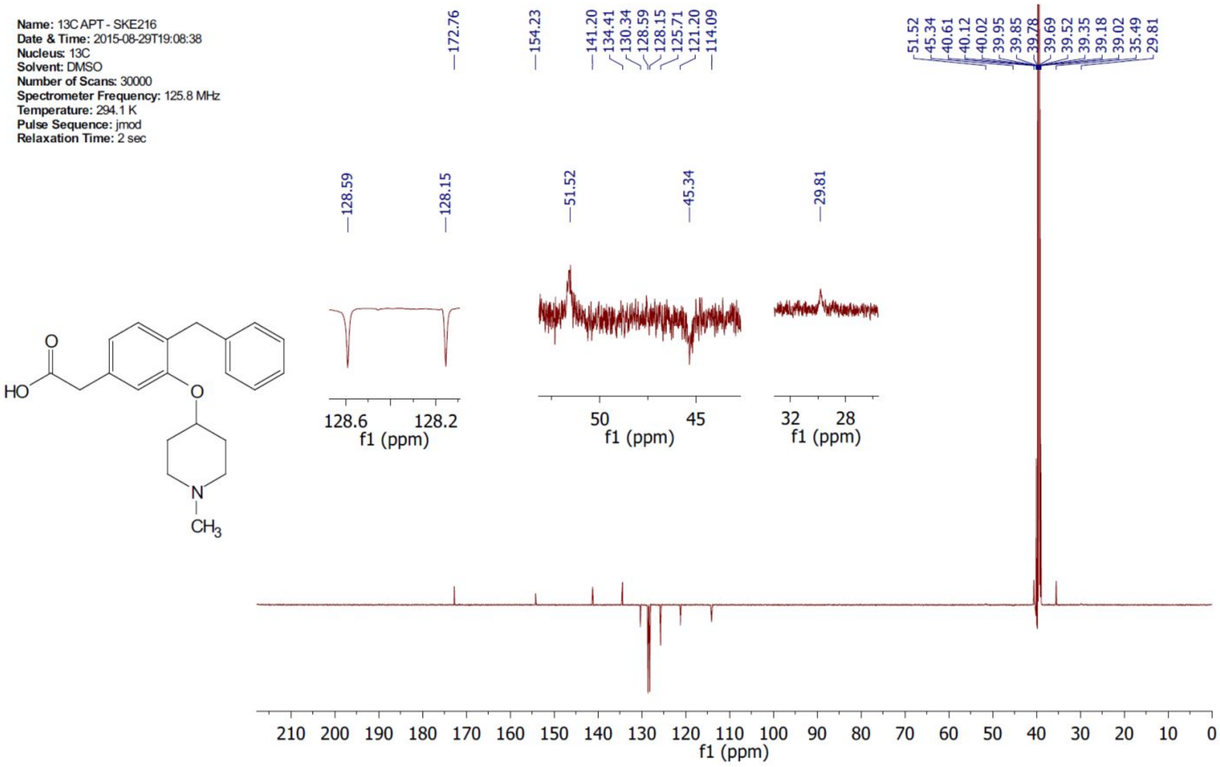
Name: 13C APT - SKE216
Date & Time: 2015-08-29T19:08:38
Nucleus: 13C
Solvent: DMSO
Number of Scans: 30000
Spectrometer Frequency: 125.8 MHz
Temperature: 294.1 K
Pulse Sequence: jmod
Relaxation Time: 2 sec
f1 (ppm)

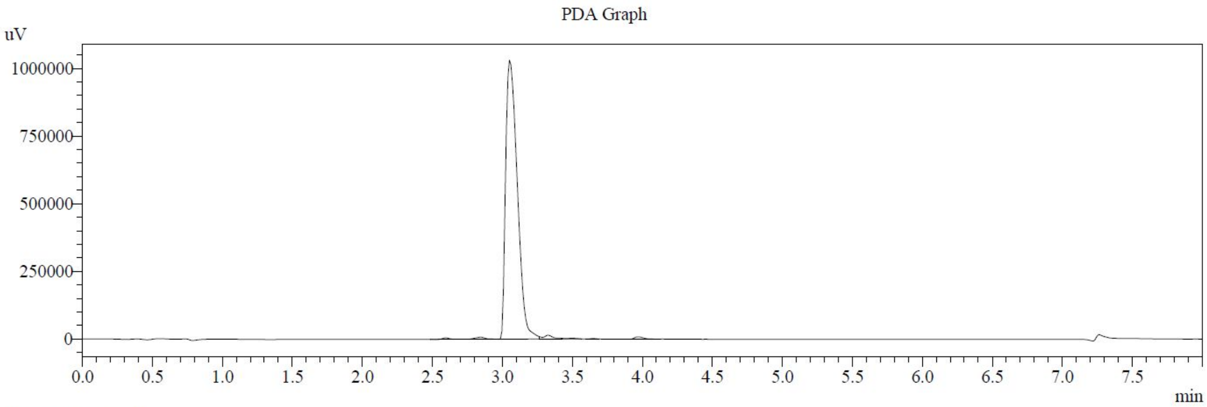

PDA Ch1 230nm 4nm

Peak#	Name	Ret. Time	Area	Area %
1		2.590	14991	0.243
2		2.840	33477	0.543
3		3.048	5991185	97.183
4		3.322	69471	1.127
5		3.492	14101	0.229
6		3.641	5381	0.087
7		3.966	36252	0.588

Compound 2c

Name: 1H - SKE241 F10
Date & Time: 2015-10-11T00:25:16
Nucleus: 1H
Solvent: DMSO
Number of Scans: 16
Spectrometer Frequency: 500.23 MHz
Temperature: 298.2 K
Pulse Sequence: zg30
Relaxation Time: 1 sec

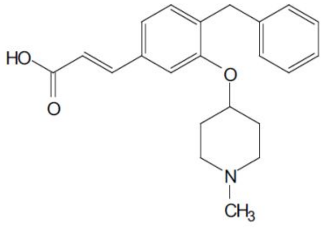

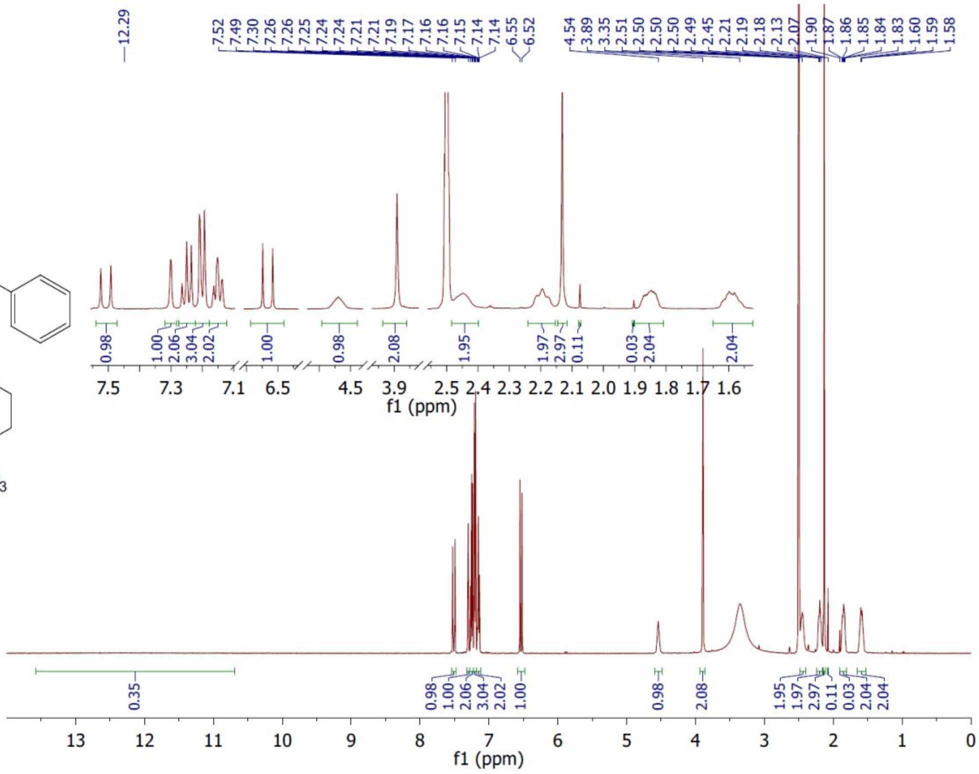

Name: 13C APT - SKE241 F10
Date & Time: 2015-10-11T06:38:18
Nucleus: 13C
Solvent: DMSO
Number of Scans: 7000
Spectrometer Frequency: 125.8 MHz
Temperature: 298.2 K
Pulse Sequence: jmod
Relaxation Time: 2 sec

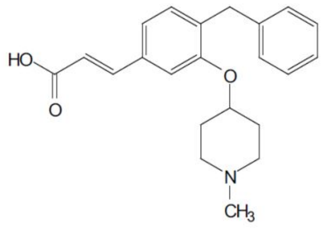

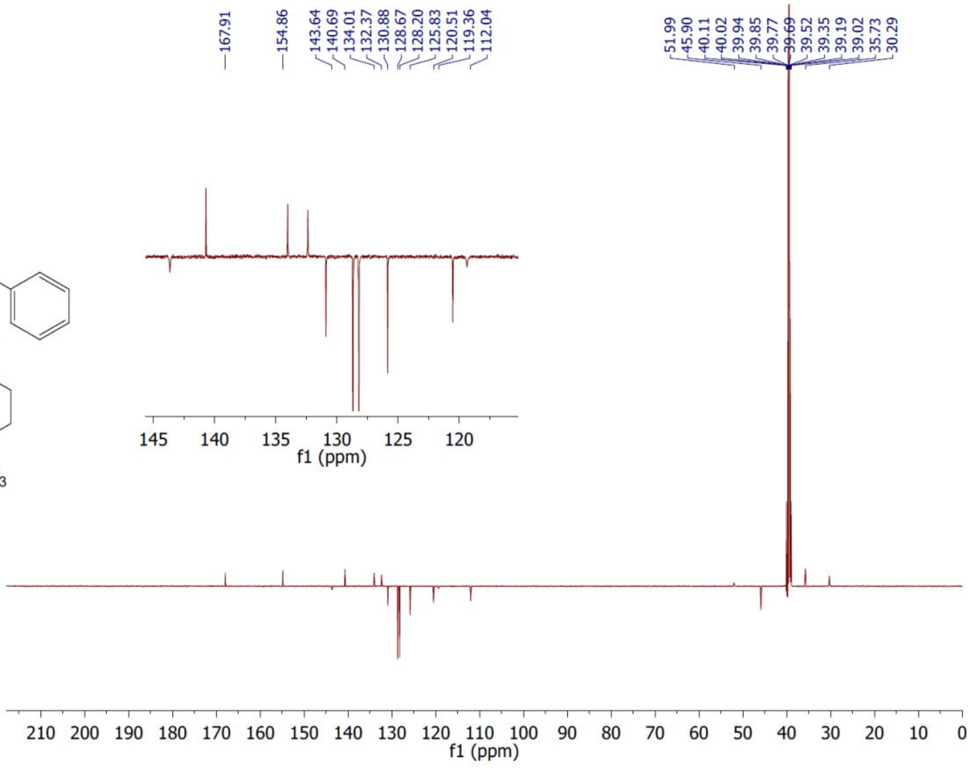

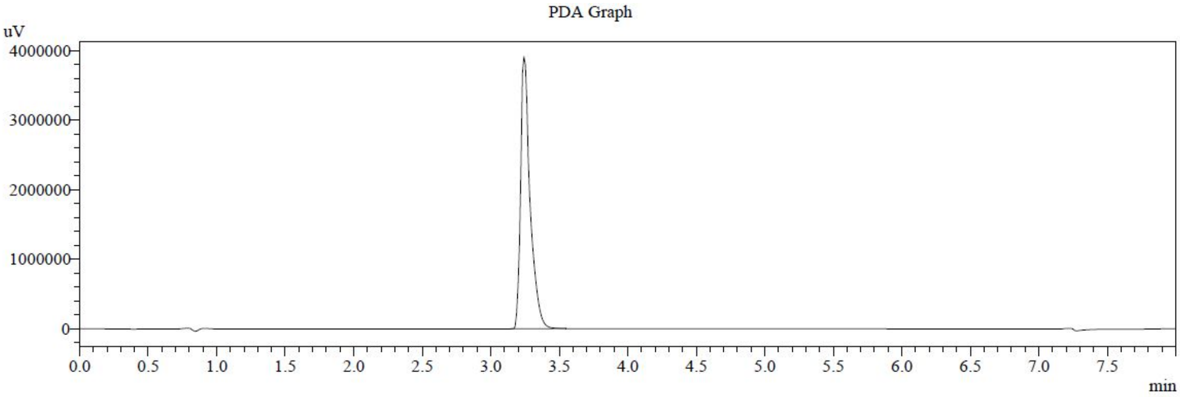

PDA Ch1 230nm 4nm

Peak#	Name	Ret. Time	Area	Area %
1		3.239	18928549	100.000

Compound 2d

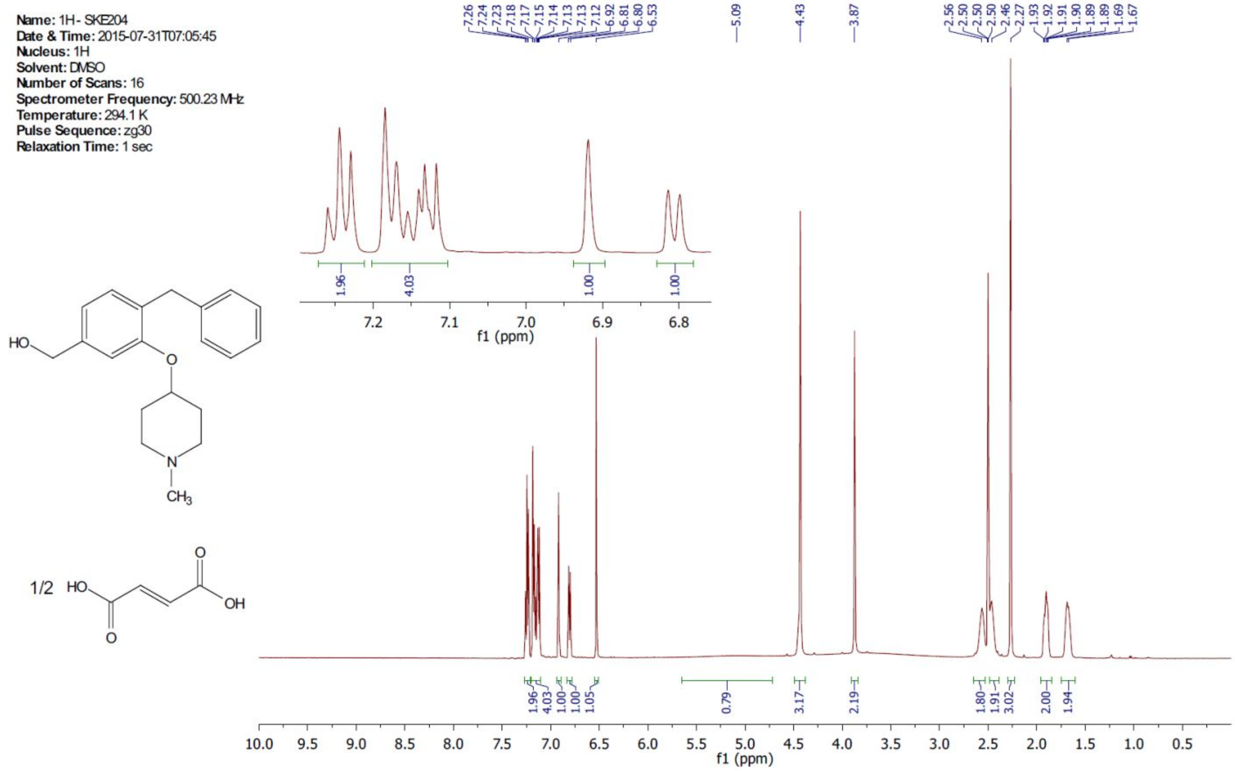

Name: 1H- SKE204
Date & Time: 2015-07-31T07:05:45
Nucleus: 1H
Solvent: DMSO
Number of Scans: 16
Spectrometer Frequency: 500.23 MHz
Temperature: 294.1 K
Pulse Sequence: zg30
Relaxation Time: 1 sec
7.26 7.24 7.23 7.18 7.17 7.15 7.14 7.13 7.13 7.12 6.92 6.81 6.80 6.53
5.09
4.43
3.87
2.56 2.50 2.50 2.50 2.46 2.27 1.93 1.92 1.91 1.90 1.89 1.89 1.69 1.67
HO
O
N
CH3
1/2 HO
O
OH
O
1.96 4.03 1.00 1.00
1.96 4.03 1.00 1.00 1.05 0.79 3.17 2.19 1.80 1.91 3.02 2.00 1.94
f1 (ppm)

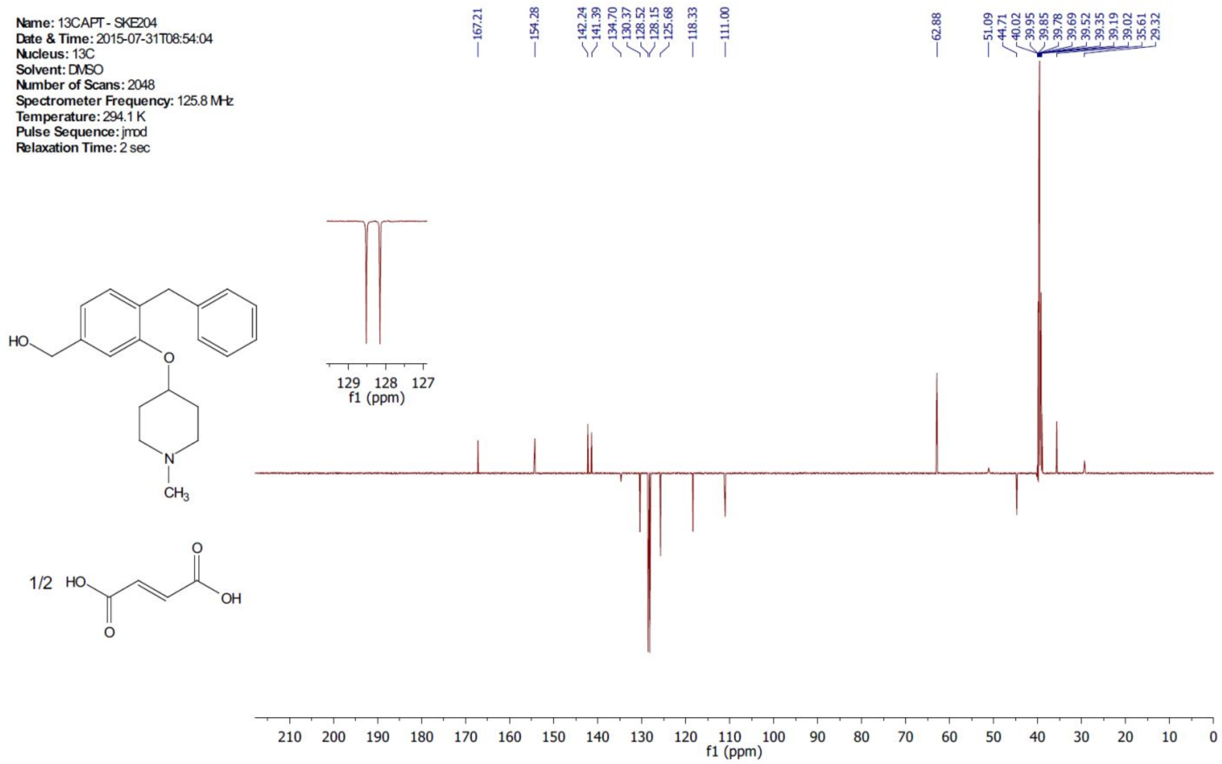

Name: 13CAPT - SKE204
Date & Time: 2015-07-31T08:54:04
Nucleus: 13C
Solvent: DMSO
Number of Scans: 2048
Spectrometer Frequency: 125.8 MHz
Temperature: 294.1 K
Pulse Sequence: jmod
Relaxation Time: 2 sec
167.21
154.28
142.24 141.39 134.70 130.37 128.52 128.15 125.68
118.33
111.00
62.88
51.09 44.71 40.02 39.95 39.85 39.78 39.69 39.52 39.35 39.19 39.02 35.61 29.32
HO
O
N
CH3
1/2 HO
O
OH
O
f1 (ppm)

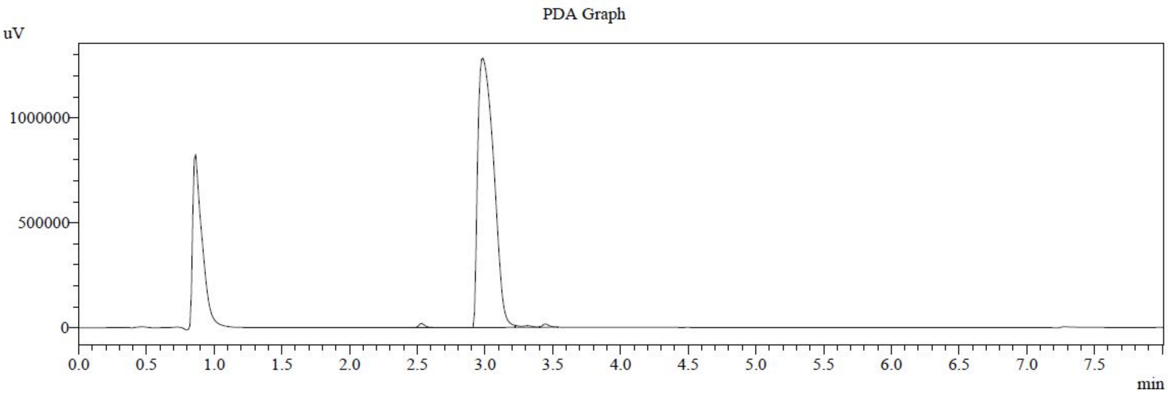

PDA Ch1 230nm 4nm

Peak#	Name	Ret. Time	Area	Area %
1		2.525	59444	0.558
2		2.978	10491820	98.468
3		3.309	53885	0.506
4		3.441	49894	0.468

Compound 2e

Name: 1H- MAST065
Date & Time: 2015-04-10T04:44:01
Nucleus: 1H
Solvent: DMSO
Number of Scans: 16
Spectrometer Frequency: 500.23 MHz
Temperature: 303.2 K
Pulse Sequence: zg30
Relaxation Time: 1 sec

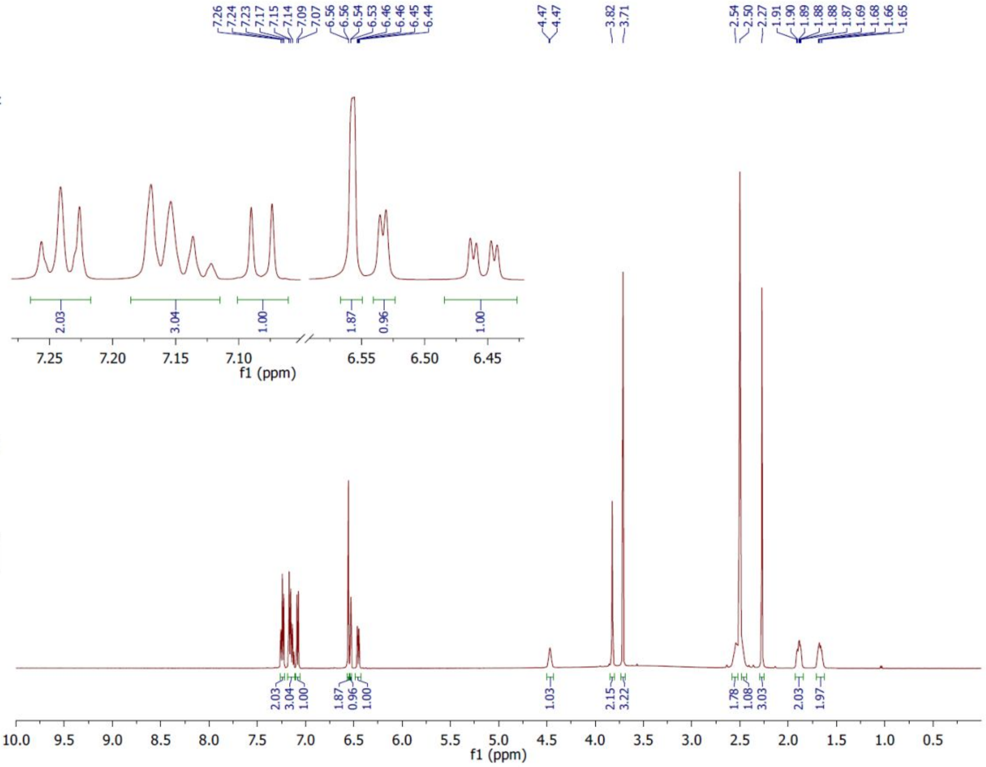

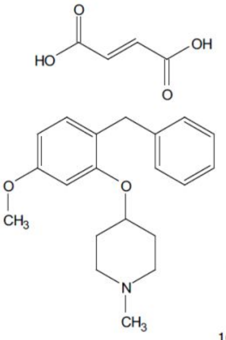

Name: 13CAPT- MAST065
Date & Time: 2015-04-10T06:32:50
Nucleus: 13C
Solvent: DMSO
Number of Scans: 2048
Spectrometer Frequency: 125.8 MHz
Temperature: 303.2 K
Pulse Sequence: jmod
Relaxation Time: 2 sec

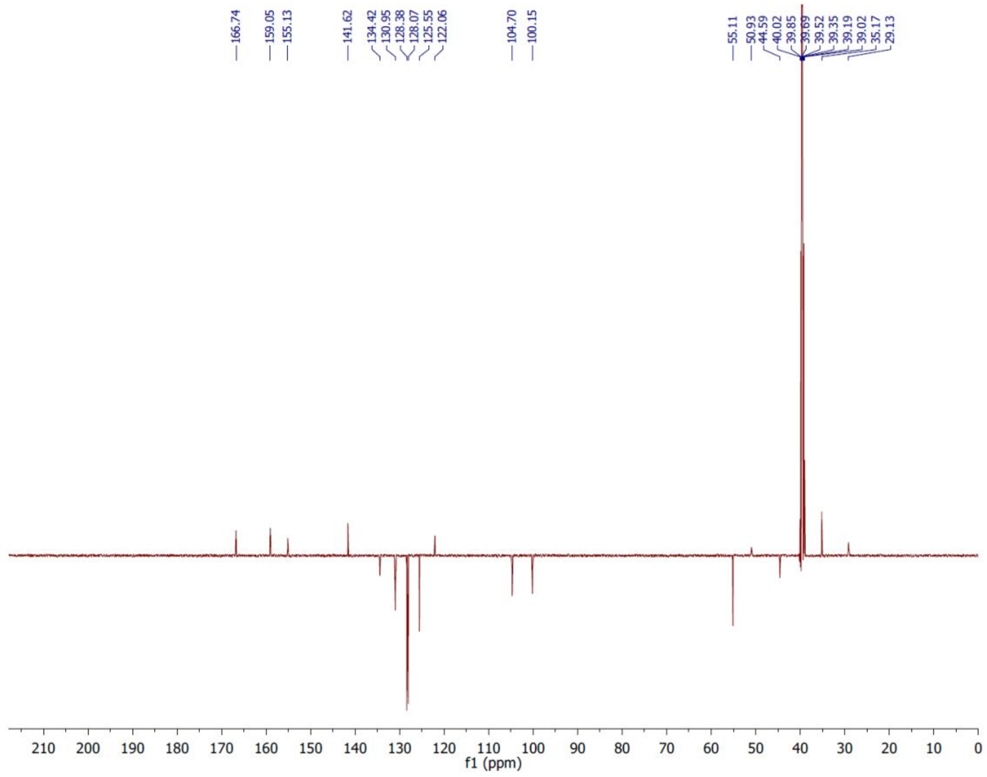

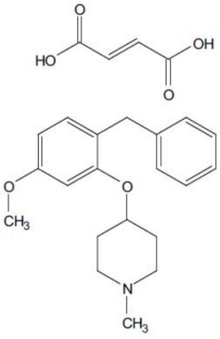

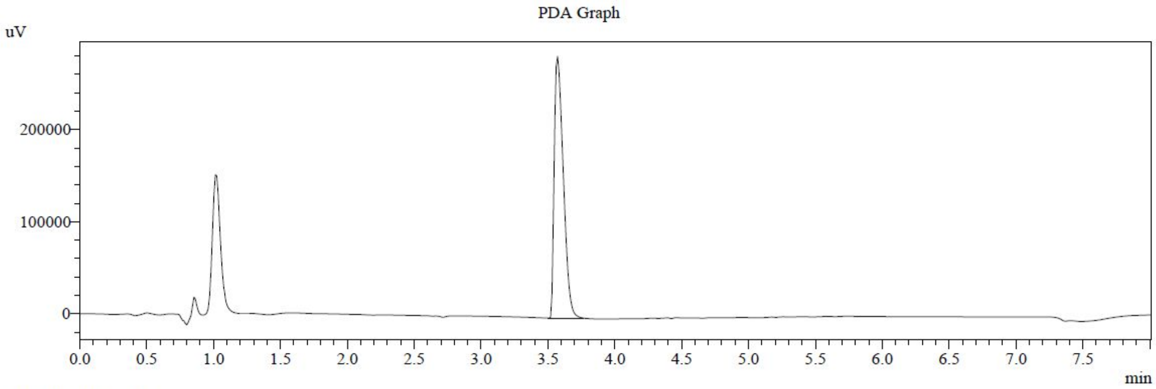

PDA Ch1 230nm 4nm

Peak#	Name	Ret. Time	Area	Area %
1		3.567	1350644	100.000

Compound 3a

Name: 1H- SKE252
Date & Time: 2015-11-10T16:54:46
Nucleus: 1H
Solvent: DMSO
Number of Scans: 16
Spectrometer Frequency: 500.23 MHz
Temperature: 298.2 K
Pulse Sequence: zg30
Relaxation Time: 1 sec

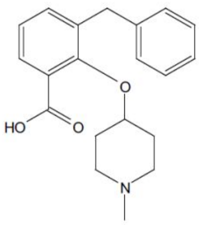

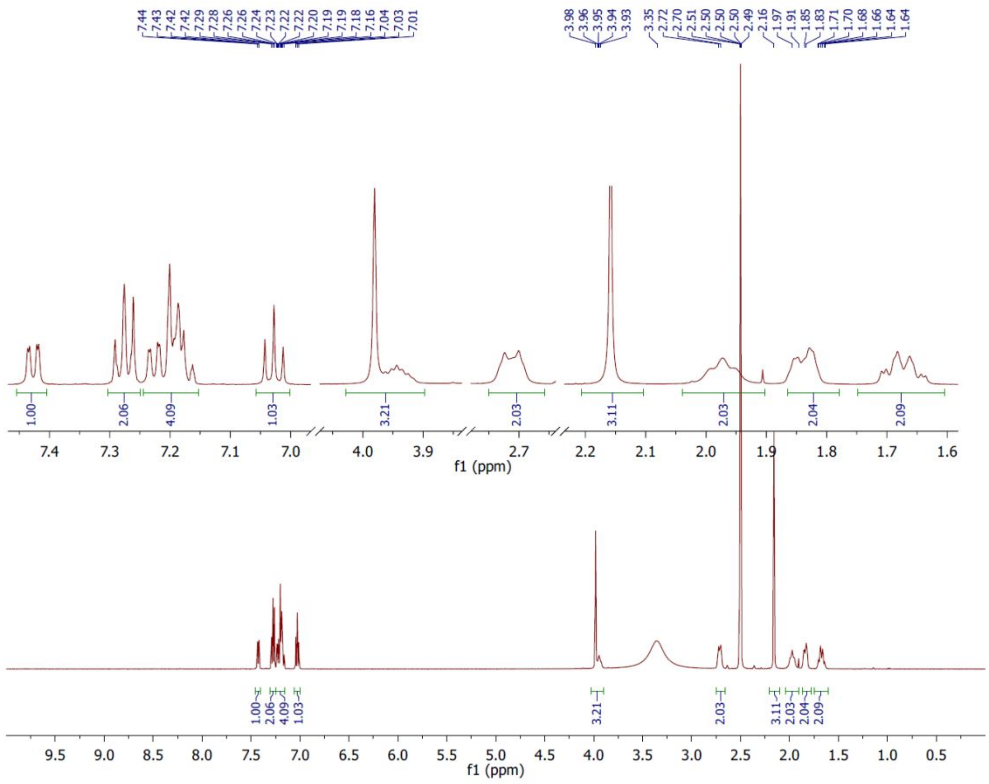

Name: 13CAPT- SKE252
Date & Time: 2015-11-11T01:41:54
Nucleus: 13C
Solvent: DMSO
Number of Scans: 10000
Spectrometer Frequency: 125.8 MHz
Temperature: 298.2 K
Pulse Sequence: jmod
Relaxation Time: 2 sec

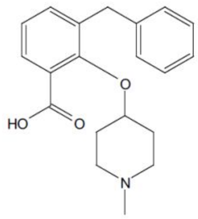

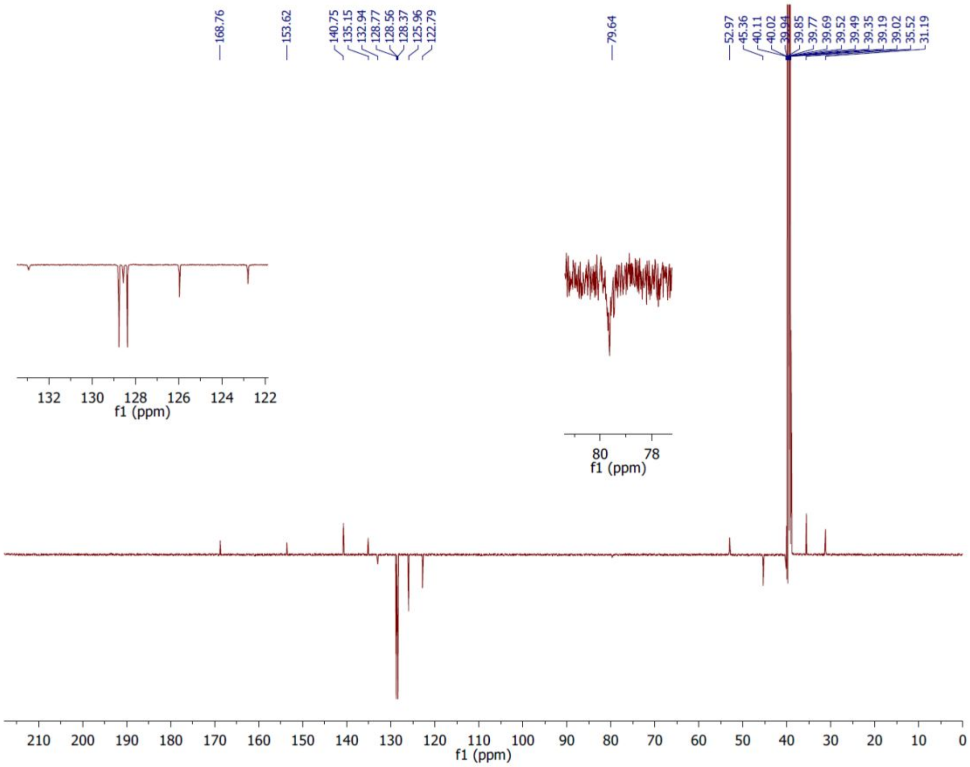

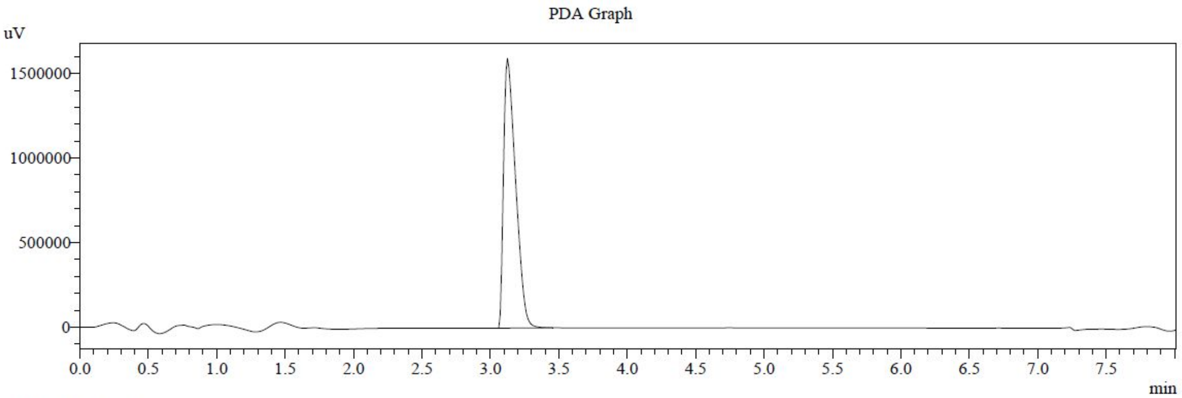

PDA Ch1 230nm 4nm

Peak#	Name	Ret. Time	Area	Area %
1		3.121	9782571	100.000

Compound 3b

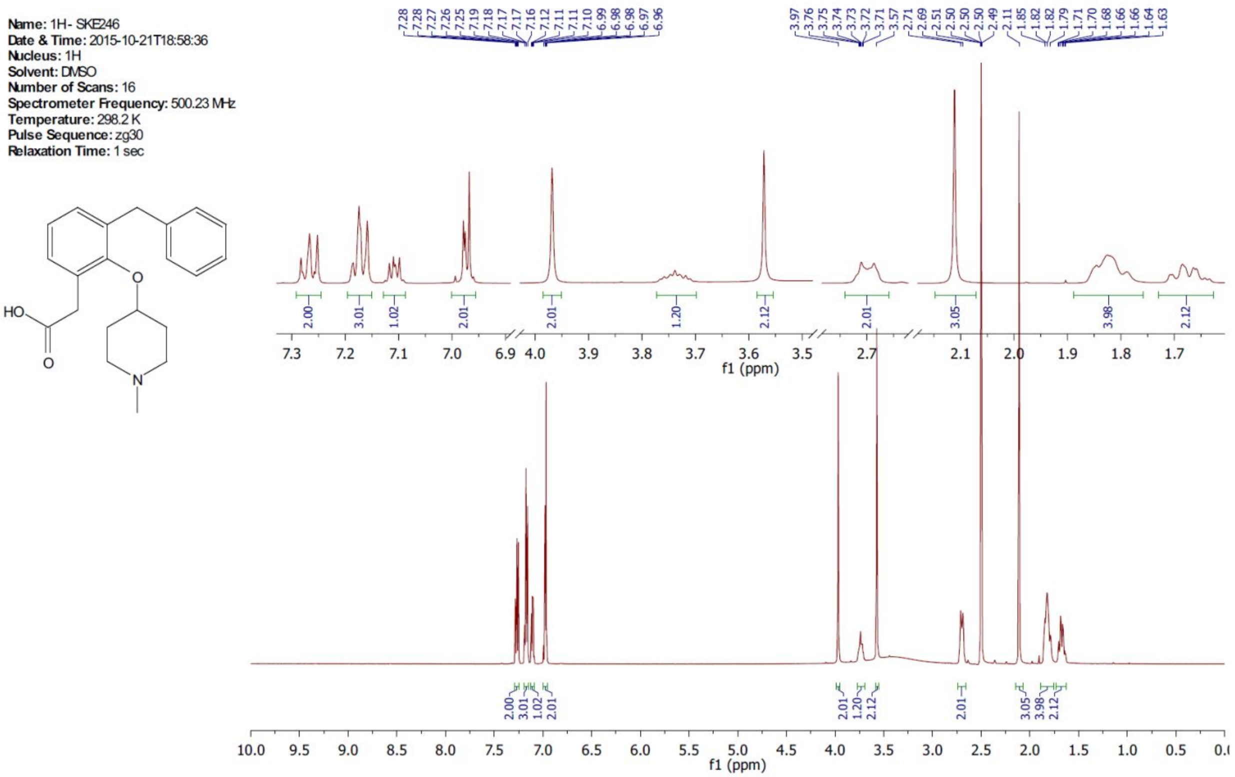

Name: 1H- SKE246
Date & Time: 2015-10-21T18:58:36
Nucleus: 1H
Solvent: DMSO
Number of Scans: 16
Spectrometer Frequency: 500.23 MHz
Temperature: 298.2 K
Pulse Sequence: zg30
Relaxation Time: 1 sec
HO
O
O
N
f1 (ppm)

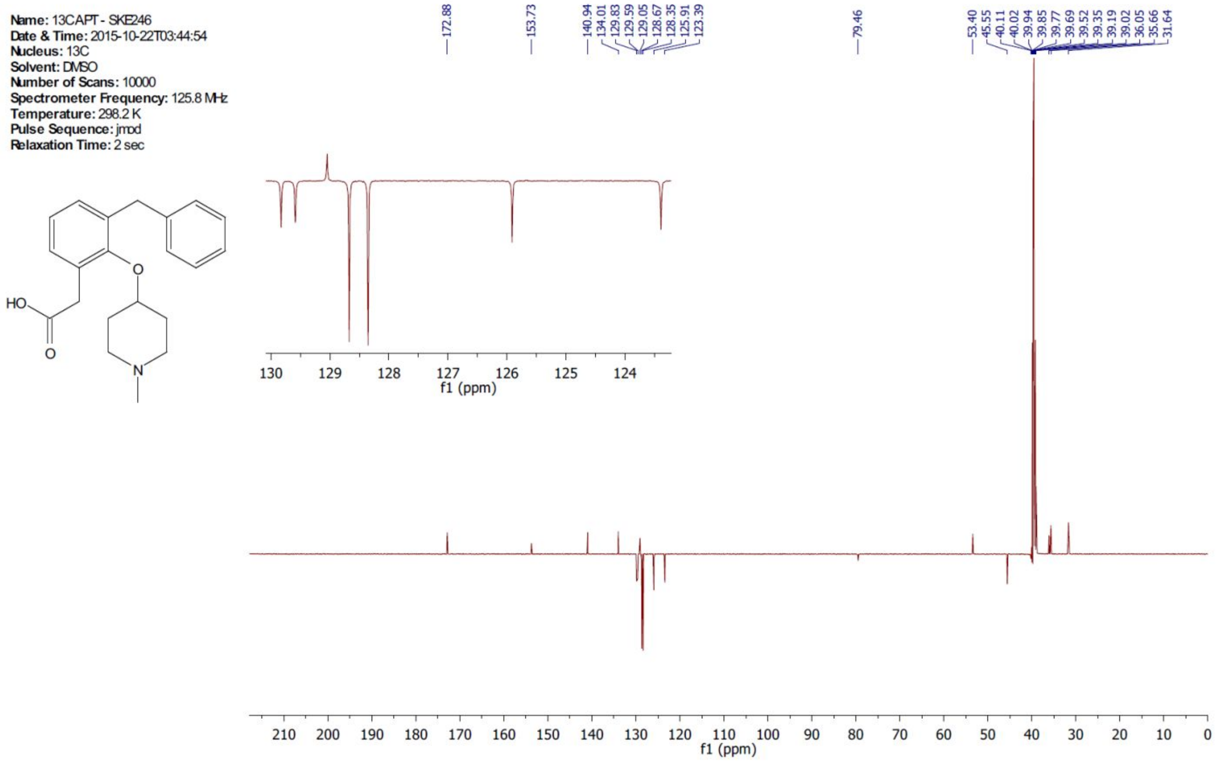

Name: 13CAPT - SKE246
Date & Time: 2015-10-22T03:44:54
Nucleus: 13C
Solvent: DMSO
Number of Scans: 10000
Spectrometer Frequency: 125.8 MHz
Temperature: 298.2 K
Pulse Sequence: jmod
Relaxation Time: 2 sec
HO
O
O
N
f1 (ppm)

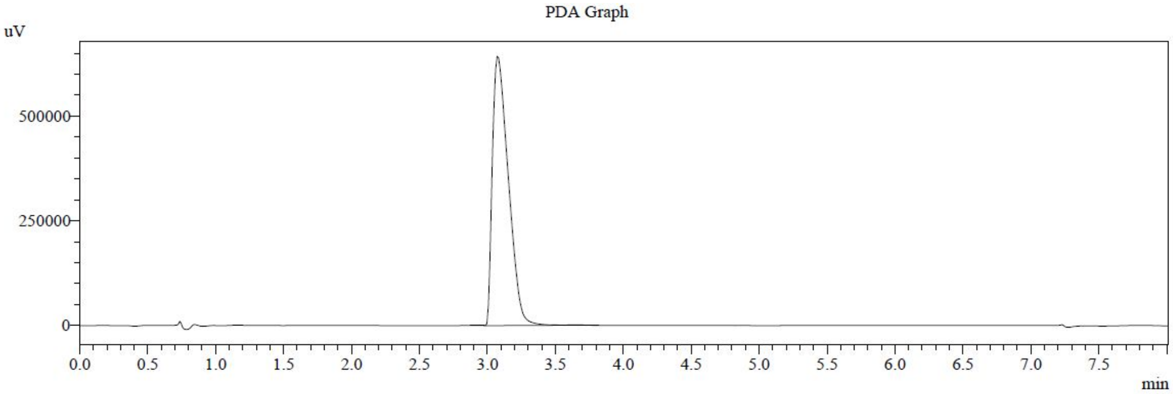

PDA Ch1 230nm 4nm

Peak#	Name	Ret. Time	Area	Area %
1		2.937	3351	0.064
2		3.071	5256516	99.655
3		3.610	9044	0.171
4		3.681	5823	0.110

Compound 3c

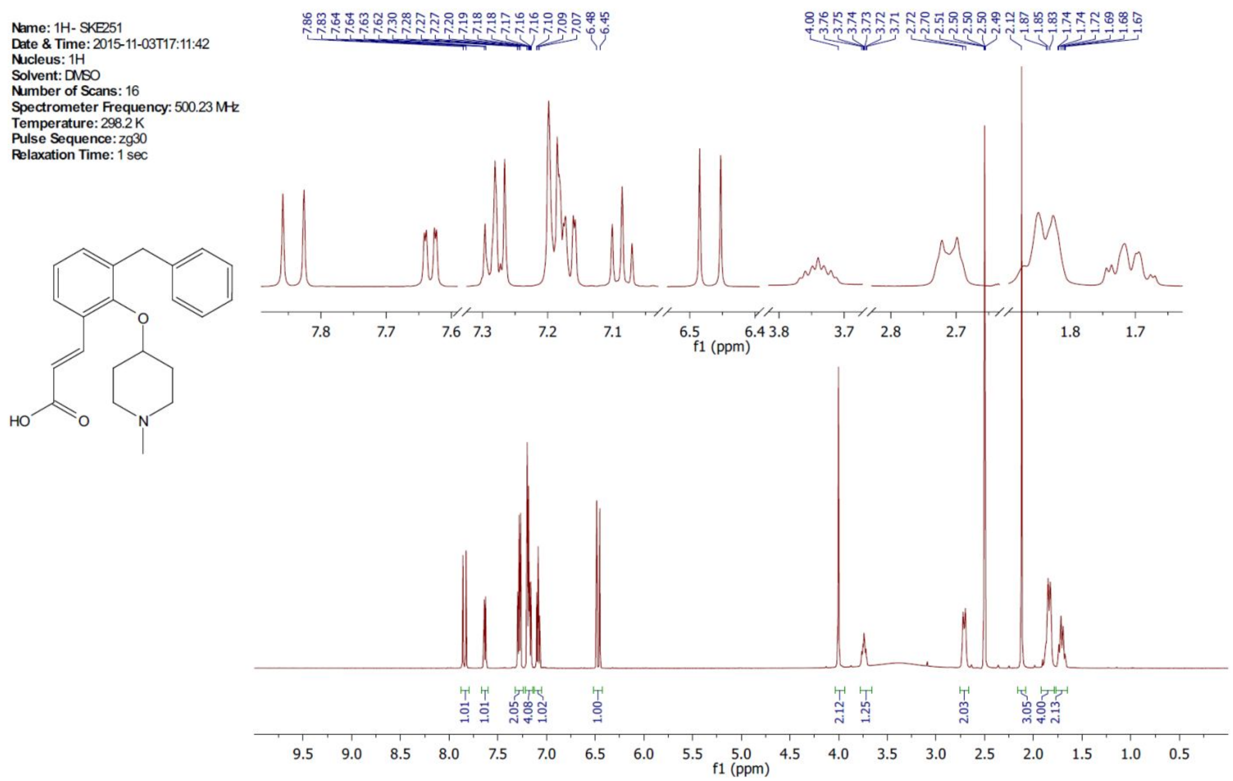
Name: 1H- SKE251
Date & Time: 2015-11-03T17:11:42
Nucleus: 1H
Solvent: DMSO
Number of Scans: 16
Spectrometer Frequency: 500.23 MHz
Temperature: 298.2 K
Pulse Sequence: zg30
Relaxation Time: 1 sec
7.86 7.83 7.64 7.64 7.63 7.62 7.30 7.28 7.27 7.27 7.20 7.19 7.18 7.18 7.17 7.16 7.16 7.10 7.09 7.07 6.48 6.45
4.00 3.76 3.75 3.74 3.73 3.72 3.71 2.72 2.70 2.51 2.50 2.50 2.50 2.49 2.12 1.87 1.85 1.83 1.74 1.74 1.72 1.69 1.68 1.67
HO O N O
1.01 1.01 2.05 4.08 1.02 1.00 2.12 1.25 2.03 3.05 4.00 2.13
f1 (ppm)

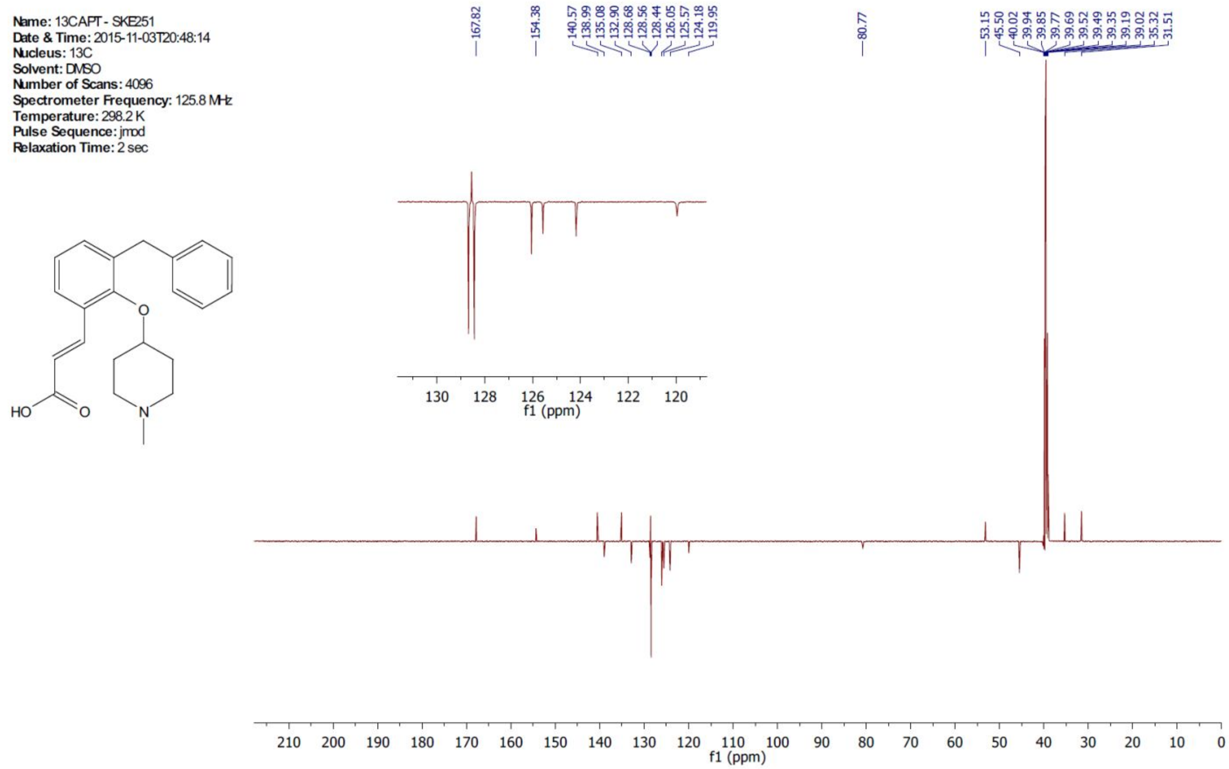
Name: 13CAPT - SKE251
Date & Time: 2015-11-03T20:48:14
Nucleus: 13C
Solvent: DMSO
Number of Scans: 4096
Spectrometer Frequency: 125.8 MHz
Temperature: 298.2 K
Pulse Sequence: jmod
Relaxation Time: 2 sec
167.82 154.38 140.57 138.99 135.08 132.90 128.68 128.56 128.44 126.05 125.57 124.18 119.95 80.77
53.15 45.50 40.02 39.94 39.85 39.77 39.69 39.52 39.49 39.35 39.19 39.02 35.32 31.51
HO O N O
f1 (ppm)

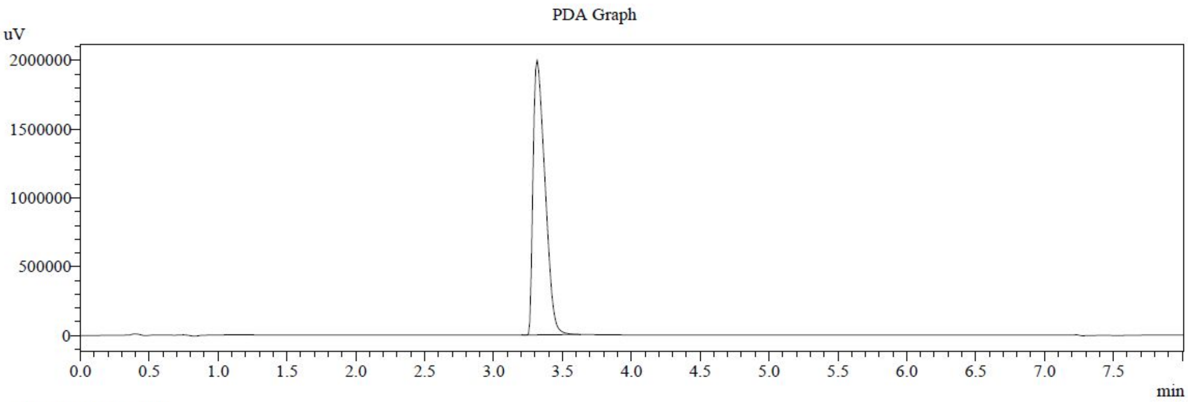

PDA Ch1 230nm 4nm

Peak#	Name	Ret. Time	Area	Area %
1		3.312	11971234	100.000

Compound 3d

Name: 1H- RIRI050
Date & Time: 2015-02-27T18:59:54
Nucleus: 1H
Solvent: DMSO
Number of Scans: 16
Spectrometer Frequency: 500.23 MHz
Temperature: 293.2 K
Pulse Sequence: zg30
Relaxation Time: 1 sec

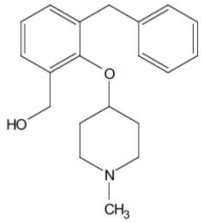

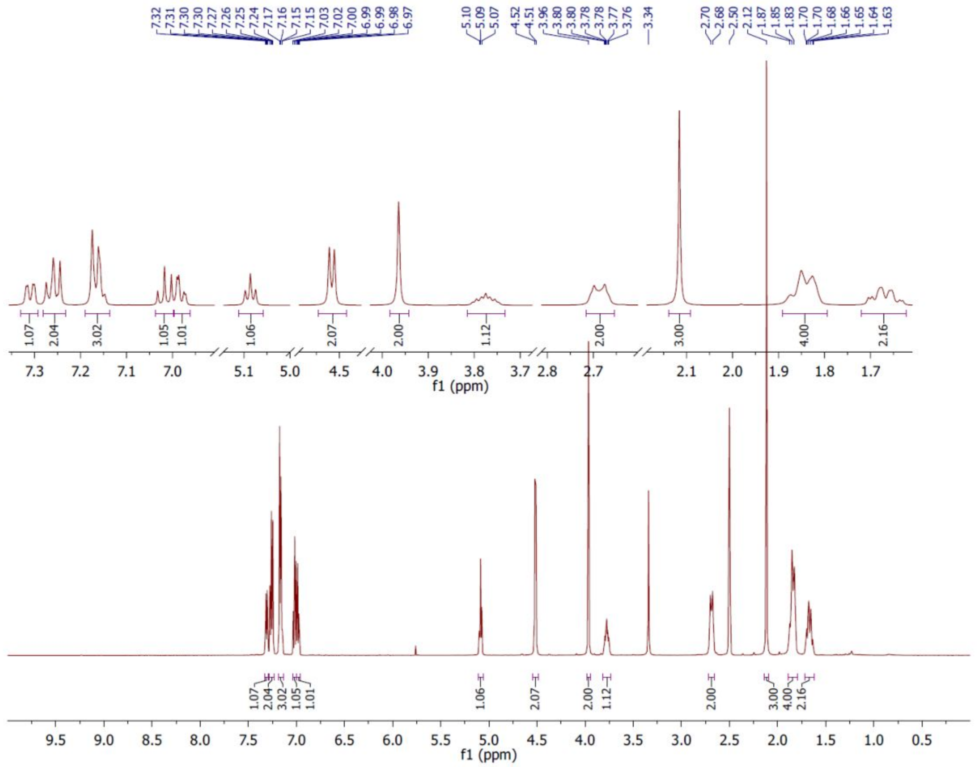

Name: 13CAPT - RIRI050
Date & Time: 2015-02-27T20:48:44
Nucleus: 13C
Solvent: DMSO
Number of Scans: 2048
Spectrometer Frequency: 125.8 MHz
Temperature: 293.2 K
Pulse Sequence: jmod
Relaxation Time: 2 sec

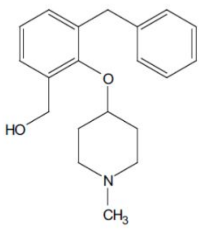

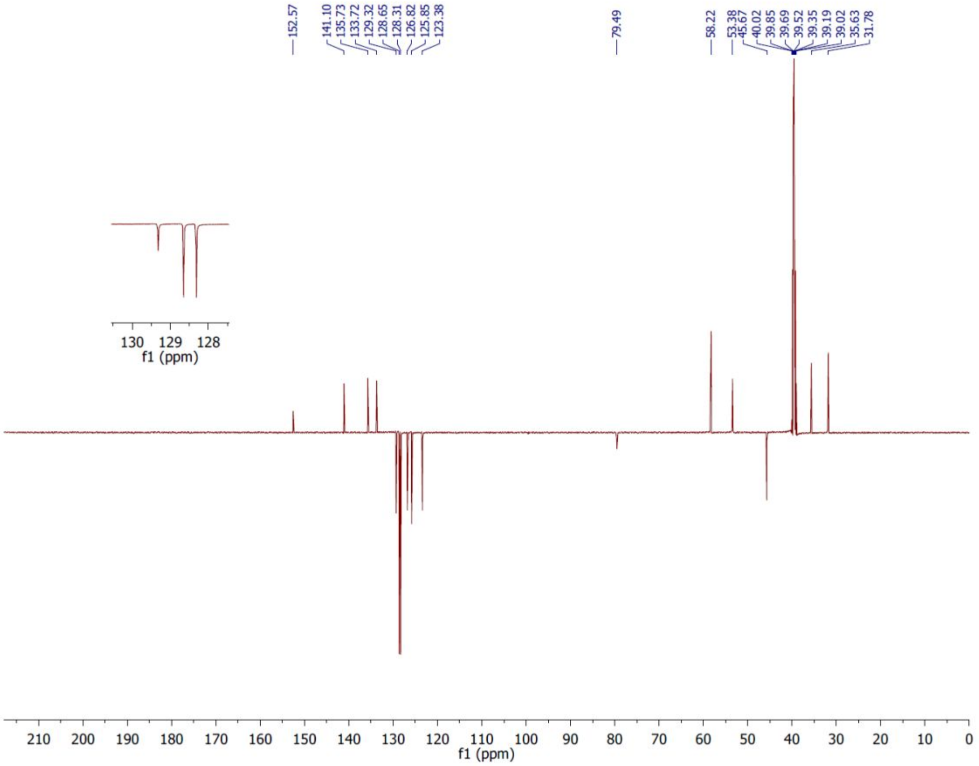

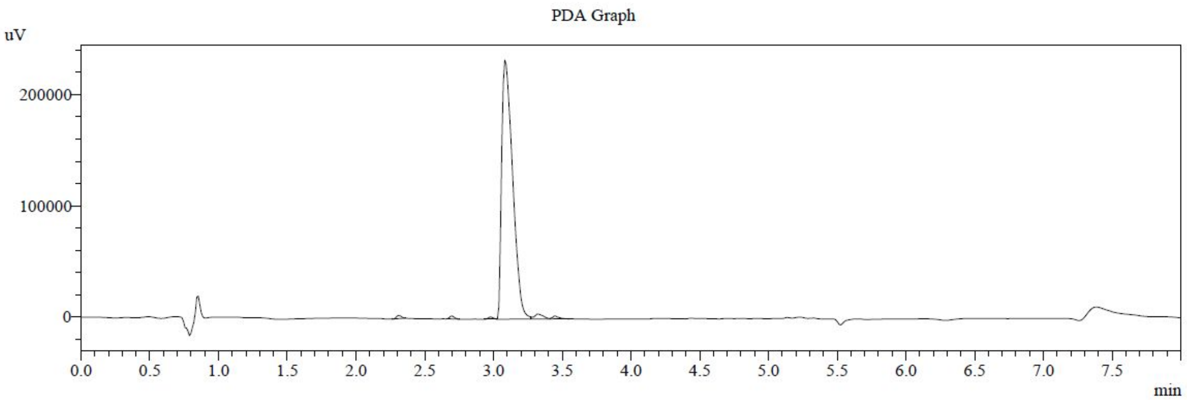

PDA Ch1 230nm 4nm

Peak#	Name	Ret. Time	Area	Area %
1		2.307	7362	0.547
2		2.692	5932	0.441
3		2.974	5000	0.372
4		3.081	1299638	96.581
5		3.318	19683	1.463
6		3.442	8031	0.597

Compound 4a

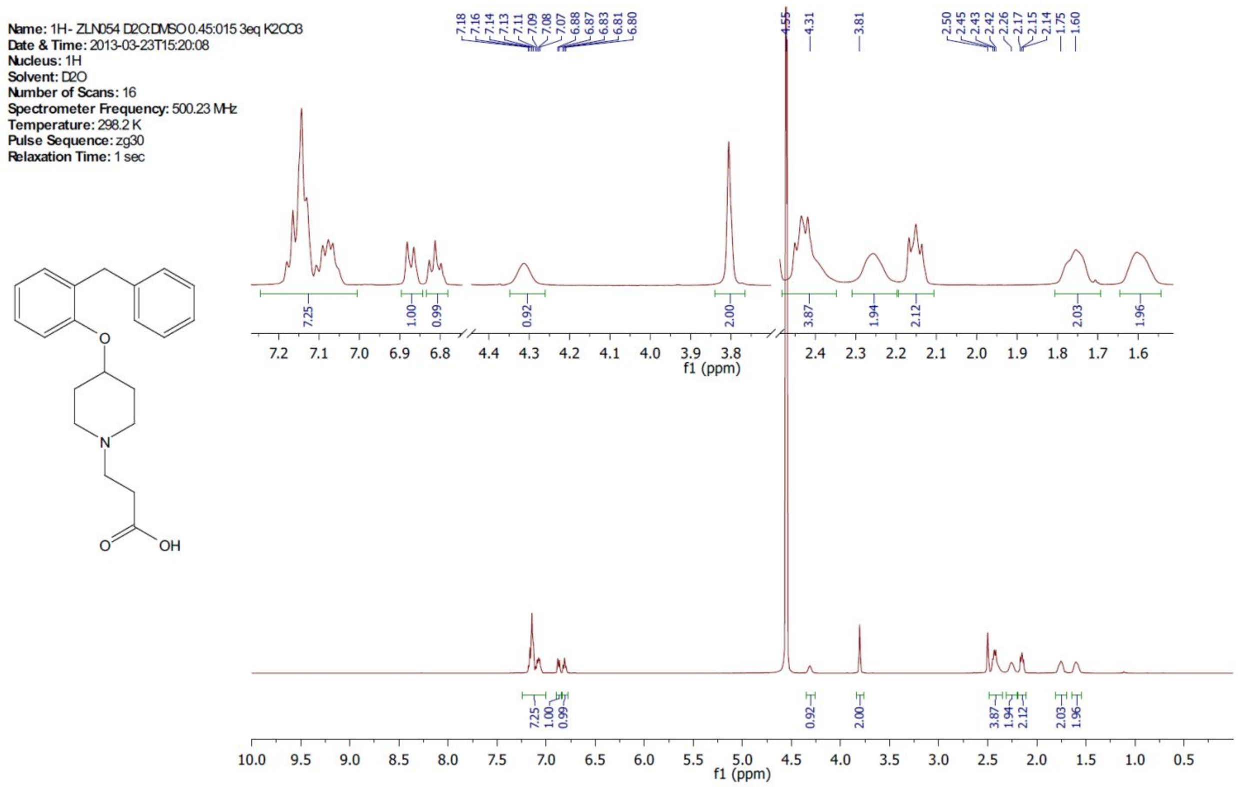
Name: 1H- ZLN054 D2O:DMSO 0.45:015 3eq K2CO3
Date & Time: 2013-03-23T15:20:08
Nucleus: 1H
Solvent: D2O
Number of Scans: 16
Spectrometer Frequency: 500.23 MHz
Temperature: 298.2 K
Pulse Sequence: zg30
Relaxation Time: 1 sec
OH
f1 (ppm)

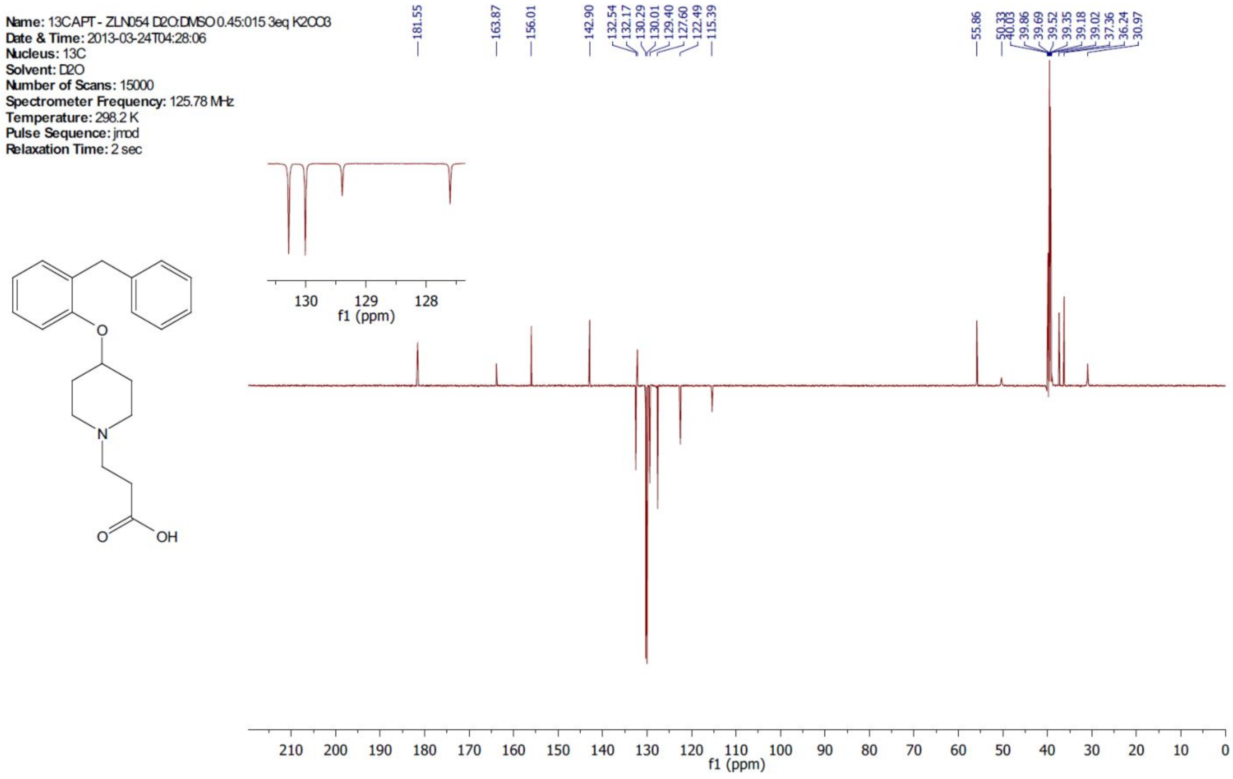
Name: 13CAPT - ZLN054 D2O:DMSO 0.45:015 3eq K2CO3
Date & Time: 2013-03-24T04:28:06
Nucleus: 13C
Solvent: D2O
Number of Scans: 15000
Spectrometer Frequency: 125.78 MHz
Temperature: 298.2 K
Pulse Sequence: jmod
Relaxation Time: 2 sec
OH
f1 (ppm)

Chromatogram
ZLN054_P_Acid C:\LabSolutions\Data\2013\2013-wk35\ZLN054_P_Acid.lcd

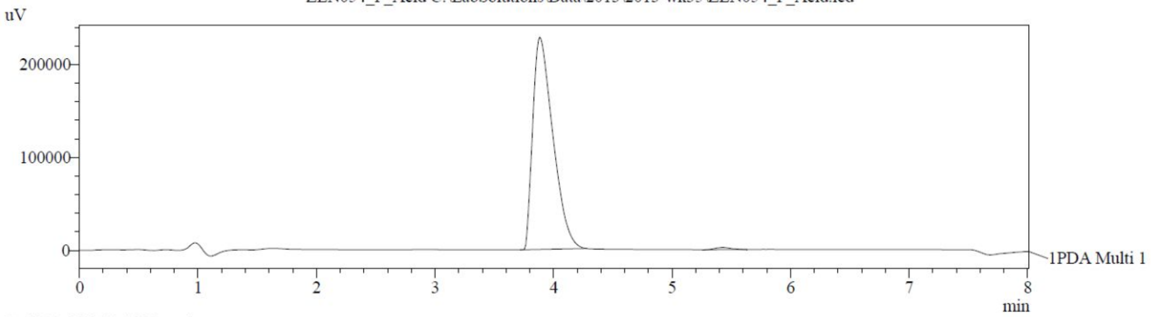

1 PDA Multi 1 / 230nm 4nm

PeakTable

PDA Ch1 230nm 4nm

Peak#	Name	Ret. Time	Area	Area %
1		3.879	2695523	99.217
2		5.423	21279	0.783

Compound 4b

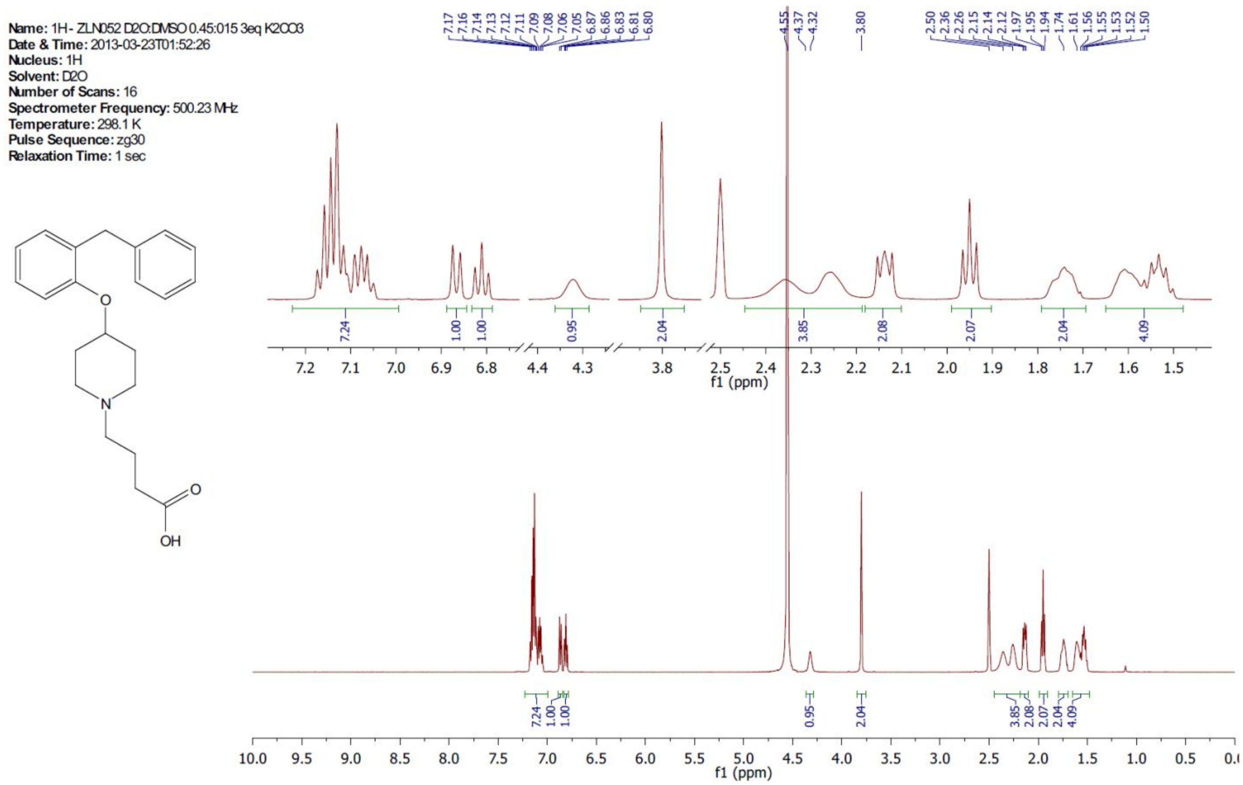
Name: 1H - ZLN052 D2O:DMSO 0.45:015 3eq K2CO3
Date & Time: 2013-03-23T01:52:26
Nucleus: 1H
Solvent: D2O
Number of Scans: 16
Spectrometer Frequency: 500.23 MHz
Temperature: 298.1 K
Pulse Sequence: zg30
Relaxation Time: 1 sec
7.17 7.16 7.14 7.13 7.12 7.11 7.09 7.08 7.06 7.05 6.87 6.86 6.83 6.81 6.80
4.55 4.37 4.32
3.80
2.50 2.36 2.26 2.15 2.14 2.12 1.97 1.95 1.94 1.74 1.61 1.56 1.55 1.53 1.52 1.50
7.24 1.00 1.00 0.95 2.04 3.85 2.08 2.07 2.04 4.09
f1 (ppm)
O
N
O
OH

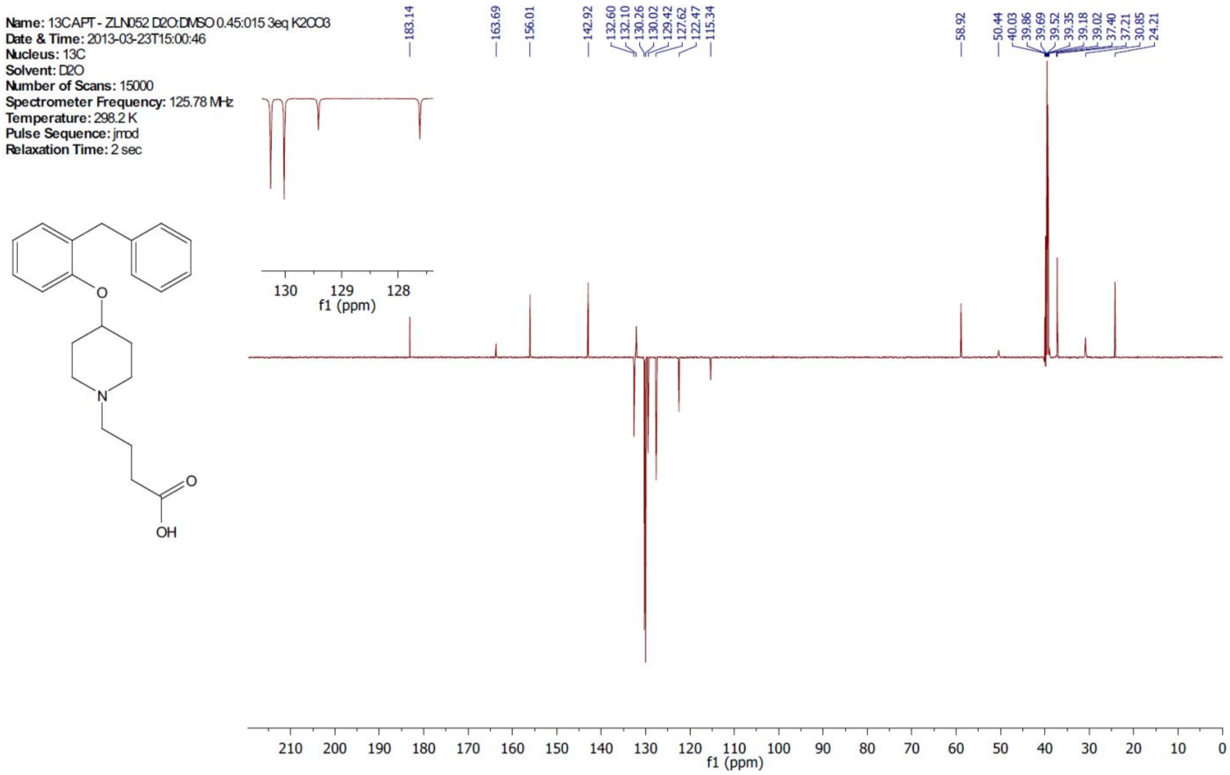
Name: 13CAPT - ZLN052 D2O:DMSO 0.45:015 3eq K2CO3
Date & Time: 2013-03-23T15:00:46
Nucleus: 13C
Solvent: D2O
Number of Scans: 15000
Spectrometer Frequency: 125.78 MHz
Temperature: 298.2 K
Pulse Sequence: jmod
Relaxation Time: 2 sec
183.14
163.69
156.01
142.92
132.60 132.10 130.26 130.02 129.42 127.62 122.47 115.34
58.92
50.44 40.03 39.86 39.69 39.52 39.35 39.18 39.02 37.40 37.21 30.85 24.21
f1 (ppm)
O
N
O
OH

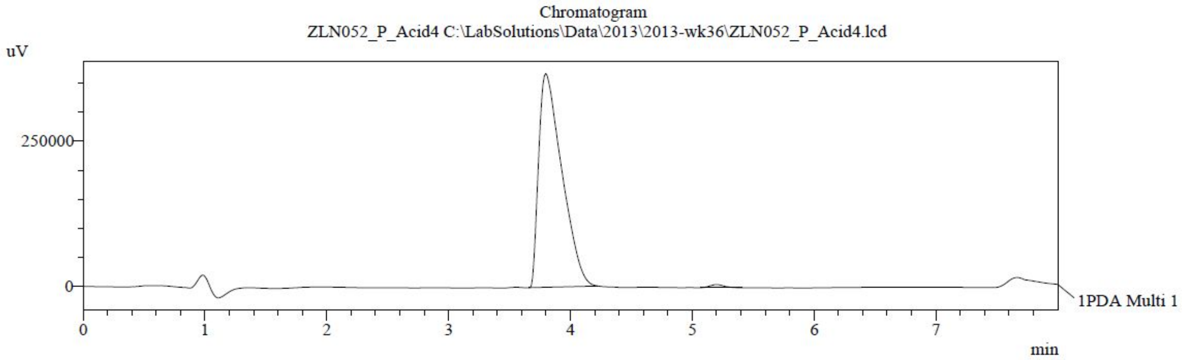

1 PDA Multi 1 / 230nm 4nm

PeakTable

PDA Ch1 230nm 4nm

Peak#	Name	Ret. Time	Area	Area %
1		3.794	4865382	99.155
2		5.193	41487	0.845

Compound 4c

Name: 1H - SKE067
Date & Time: 2012-09-14T19:31:52
Nucleus: 1H
Solvent: D2O
Number of Scans: 8
Spectrometer Frequency: 500.23 MHz
Temperature: 296.1 K
Pulse Sequence: zg30
Relaxation Time: 1 sec

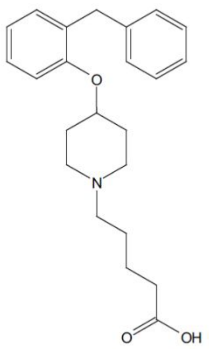

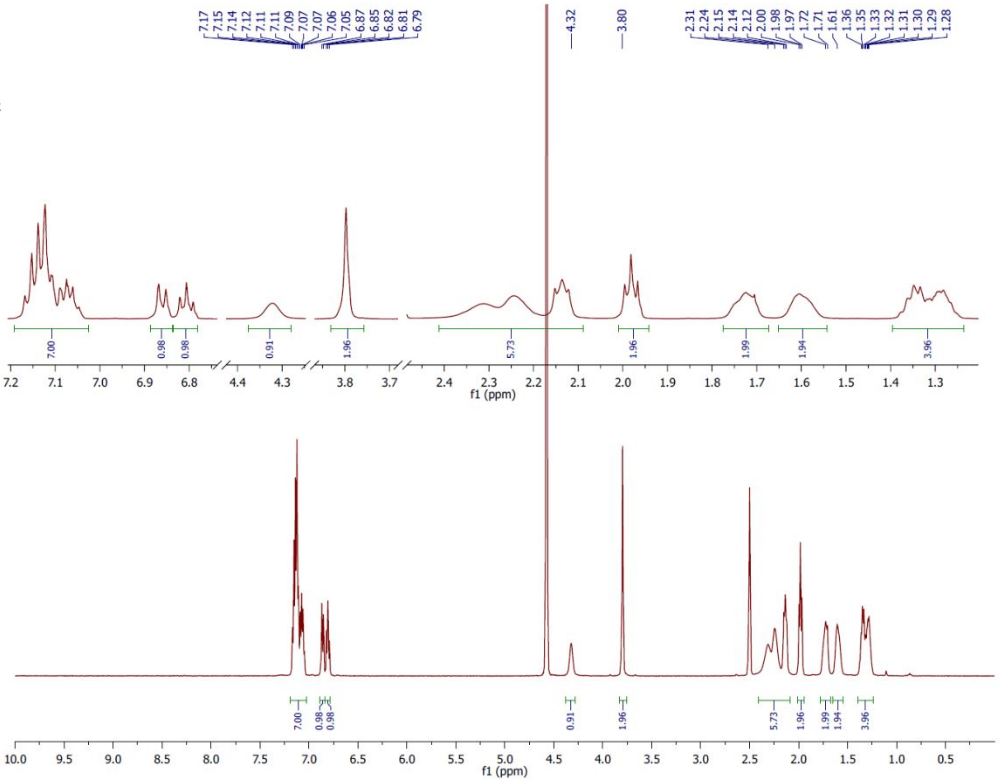

Name: 13CAPT - SKE067
Date & Time: 2012-09-15T13:26:55
Nucleus: 13C
Solvent: D2O
Number of Scans: 9725
Spectrometer Frequency: 125.78 MHz
Temperature: 296.1 K
Pulse Sequence: jmod
Relaxation Time: 2 sec

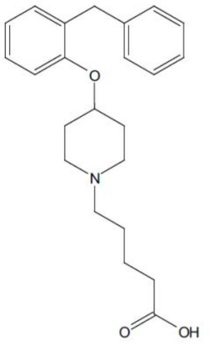

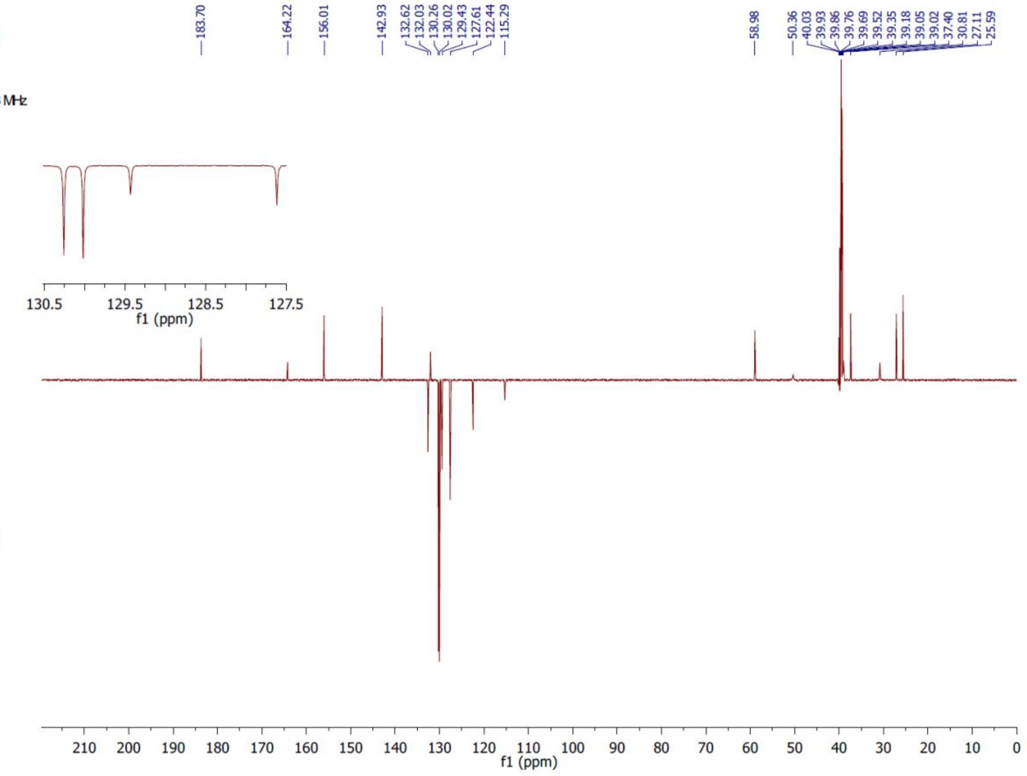

Chromatogram
SKE067_P_Acid C:\LabSolutions\Data\2013\2013-wk35\SKE067_P_Acid.lcd

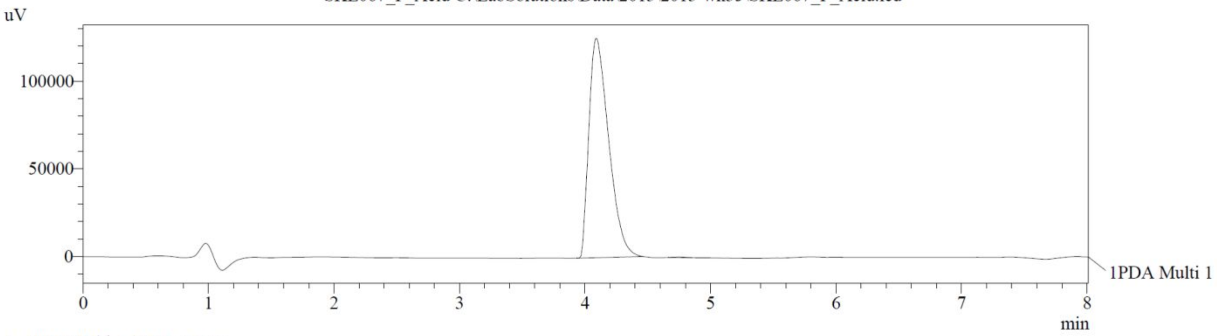

1 PDA Multi 1 / 230nm 4nm

PeakTable

PDA Ch1 230nm 4nm

Peak#	Name	Ret. Time	Area	Area %
1		4.085	1369939	99.870
2		4.750	1779	0.130

Compound 4d

Name: 1H - SKE073
Date & Time: 2012-09-14T19:40:08
Nucleus: 1H
Solvent: D2O
Number of Scans: 16
Spectrometer Frequency: 500.23 MHz
Temperature: 296 K
Pulse Sequence: zg30
Relaxation Time: 1 sec

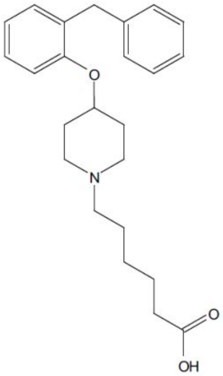

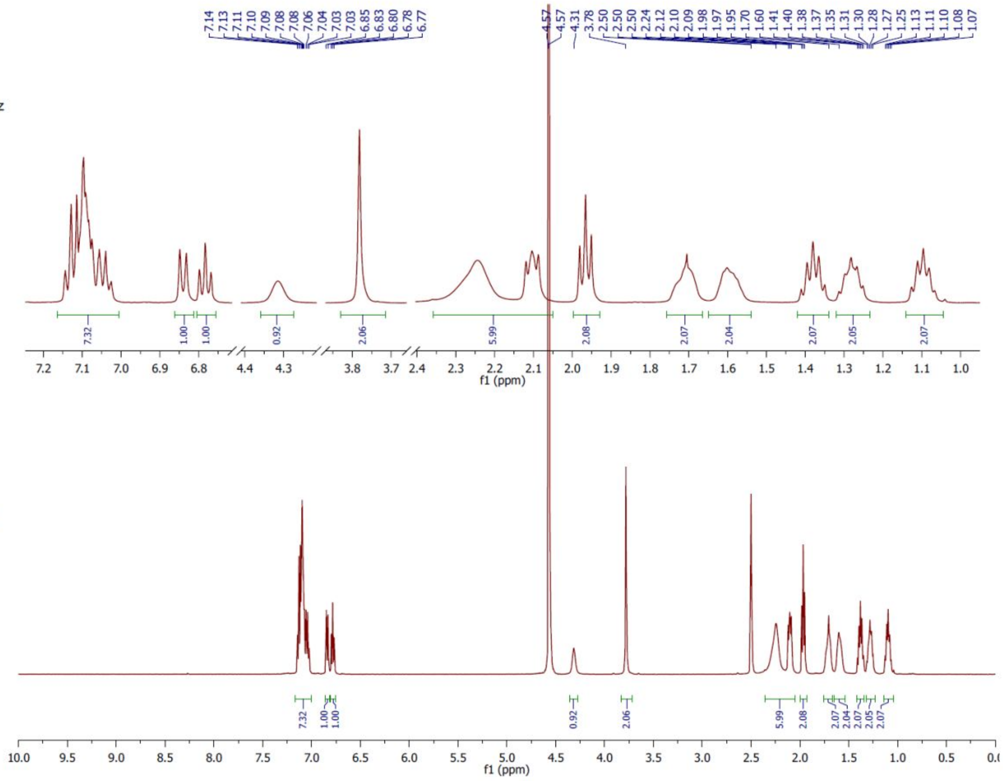

Name: 13CAPT - SKE073
Date & Time: 2012-09-15T22:17:01
Nucleus: 13C
Solvent: D2O
Number of Scans: 9725
Spectrometer Frequency: 125.78 MHz
Temperature: 296 K
Pulse Sequence: jmod
Relaxation Time: 2 sec

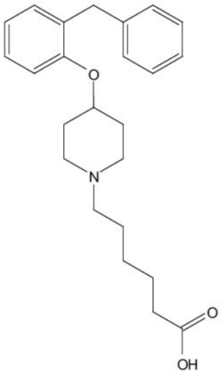

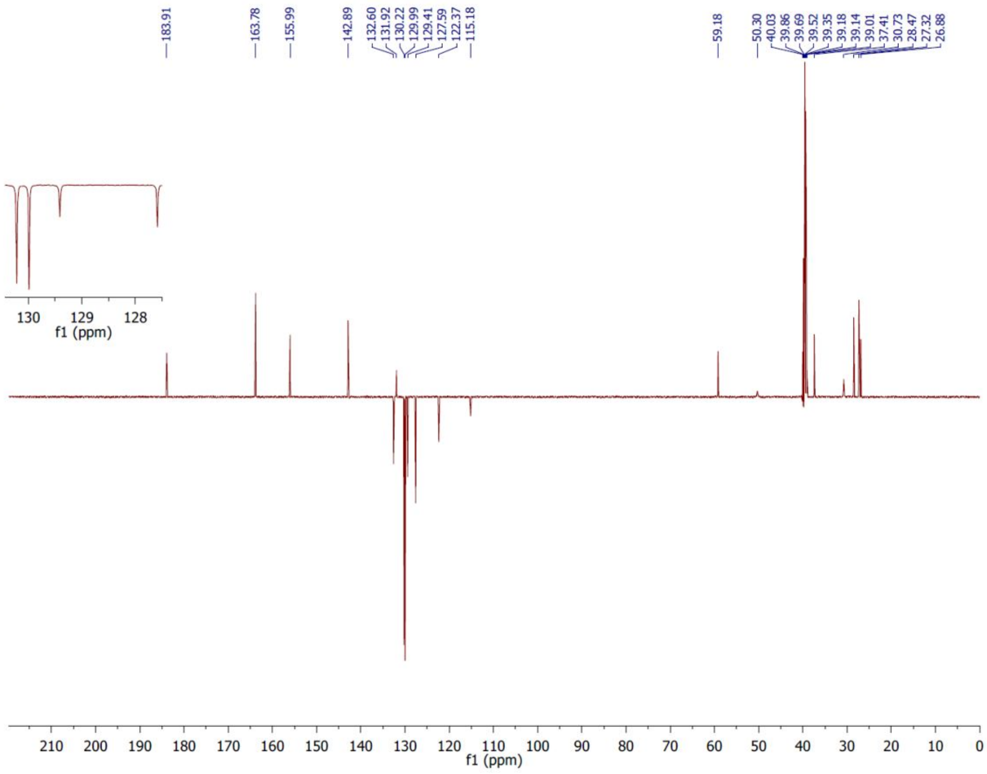

Chromatogram
SKE073_P_Acid C:\LabSolutions\Data\2013\2013-wk35\SKE073_P_Acid.lcd

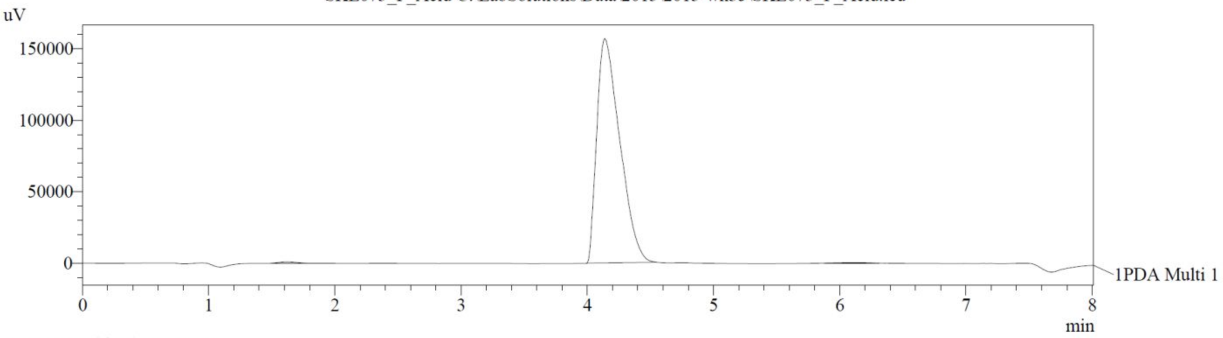

1 PDA Multi 1 / 230nm 4nm

PeakTable

PDA Ch1 230nm 4nm

Peak#	Name	Ret. Time	Area	Area %
1		1.624	9423	0.471
2		4.134	1984542	99.254
3		6.107	5489	0.275

Compound 4e

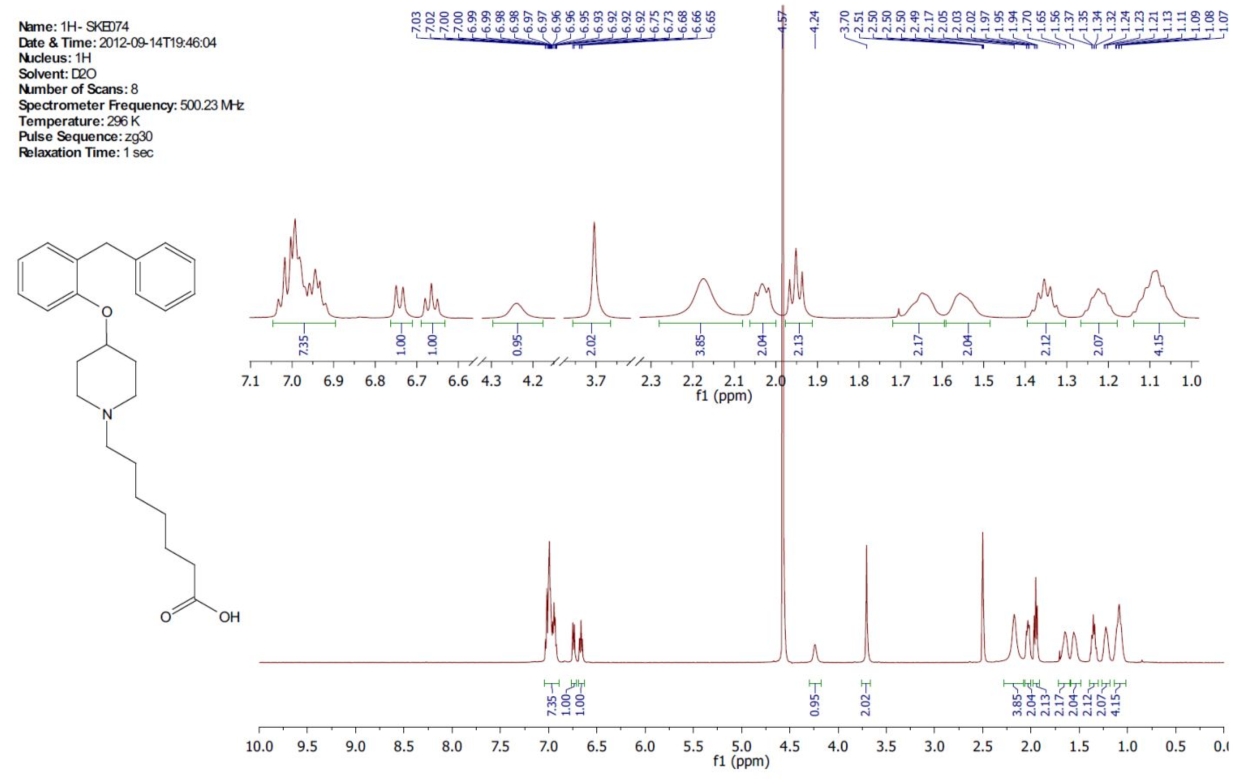
Name: 1H- SKE074
Date & Time: 2012-09-14T19:46:04
Nucleus: 1H
Solvent: D2O
Number of Scans: 8
Spectrometer Frequency: 500.23 MHz
Temperature: 296 K
Pulse Sequence: zg30
Relaxation Time: 1 sec
f1 (ppm)

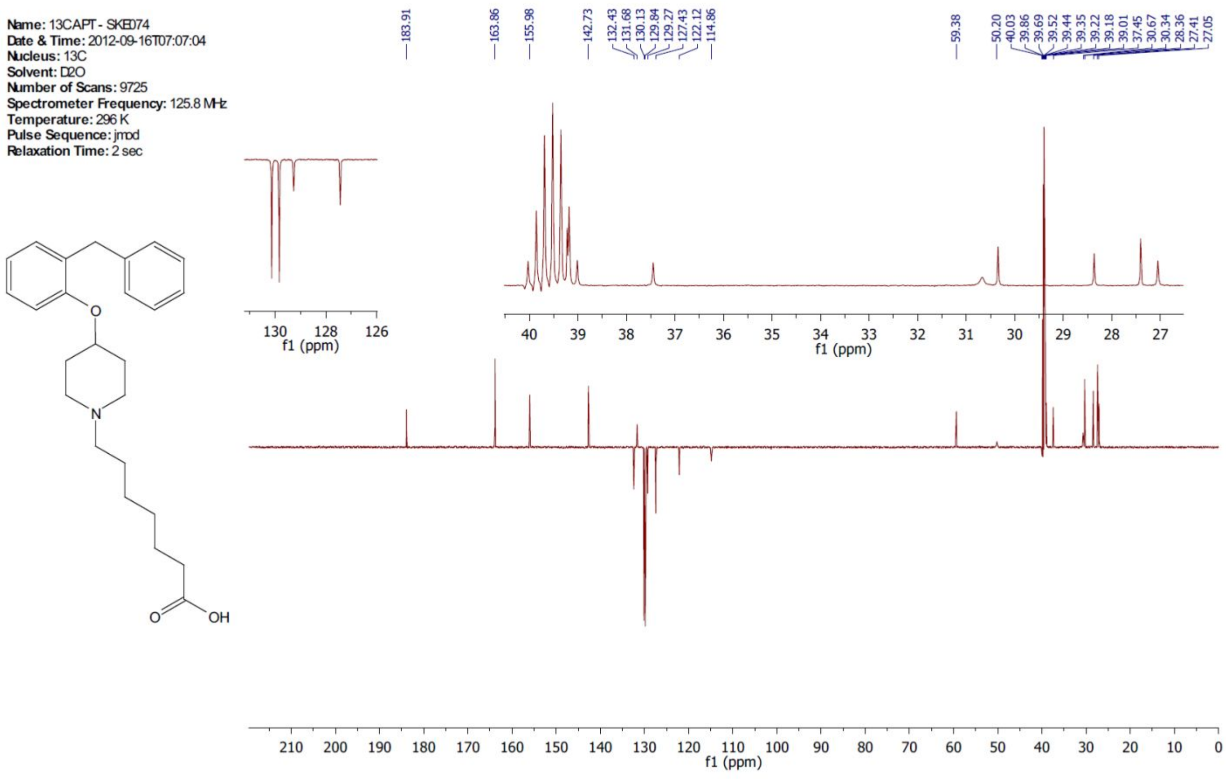
Name: 13CAPT - SKE074
Date & Time: 2012-09-16T07:07:04
Nucleus: 13C
Solvent: D2O
Number of Scans: 9725
Spectrometer Frequency: 125.8 MHz
Temperature: 296 K
Pulse Sequence: jmod
Relaxation Time: 2 sec
f1 (ppm)

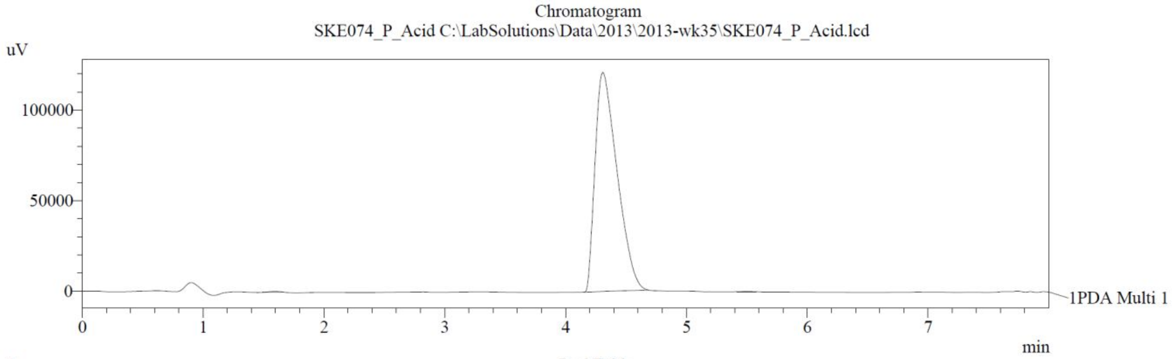

1

PeakTable

PDA Ch1 230nm 4nm

Peak#	Name	Ret. Time	Area	Area %
1		1.588	2092	0.136
2		4.304	1533862	99.822
3		5.482	638	0.042

Compound 4f

Name: 1H- ZLN055 3EQ K2CO3 D2O:DMSO 0,45:0,15
Date & Time: 2013-03-27T19:40:36
Nucleus: 1H
Solvent: D2O
Number of Scans: 16
Spectrometer Frequency: 400.13 MHz
Temperature: 293 K
Pulse Sequence: zg30
Relaxation Time: 1 sec

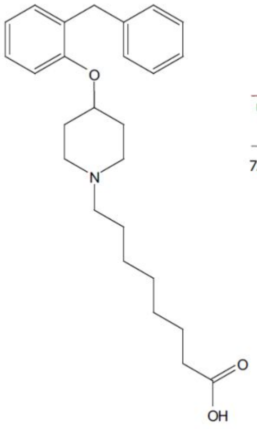

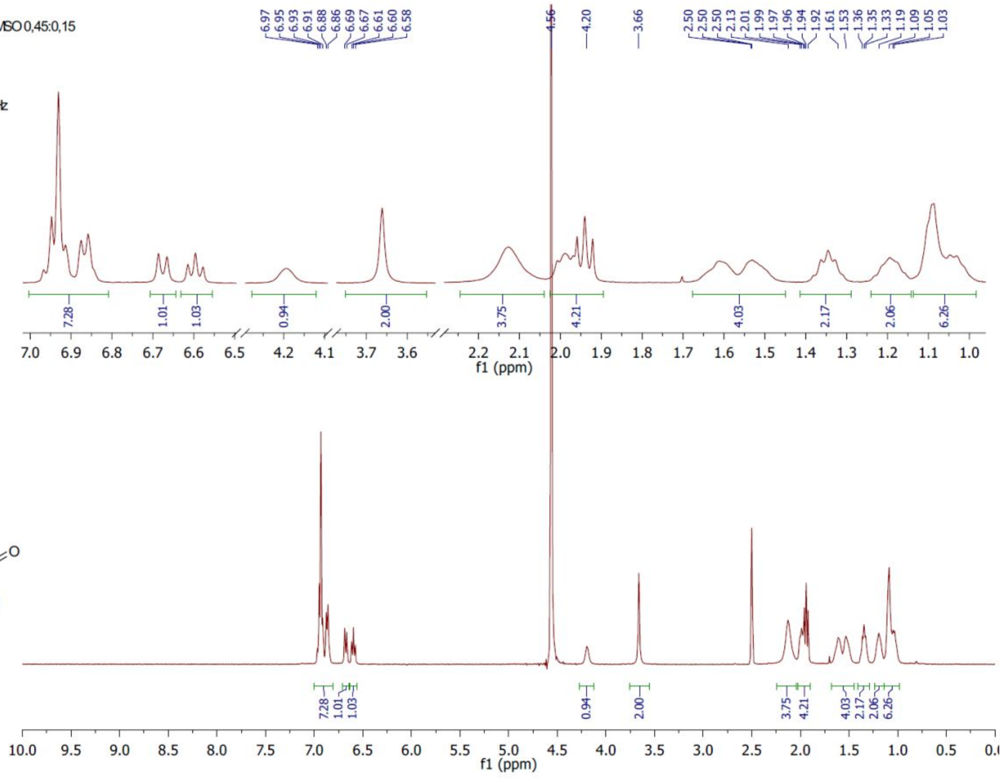

Name: C13 APT - ZLN055 3EQ K2CO3 D2O:DMSO 0,45:0,15
Date & Time: 2013-03-28T07:21:20
Nucleus: 13C
Solvent: D2O
Number of Scans: 12288
Spectrometer Frequency: 100.61 MHz
Temperature: 293 K
Pulse Sequence: jmod
Relaxation Time: 2 sec

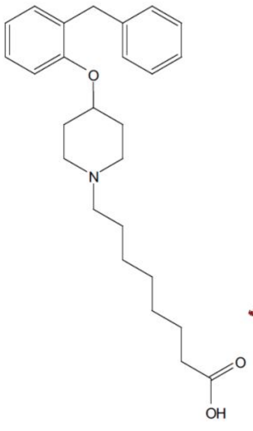

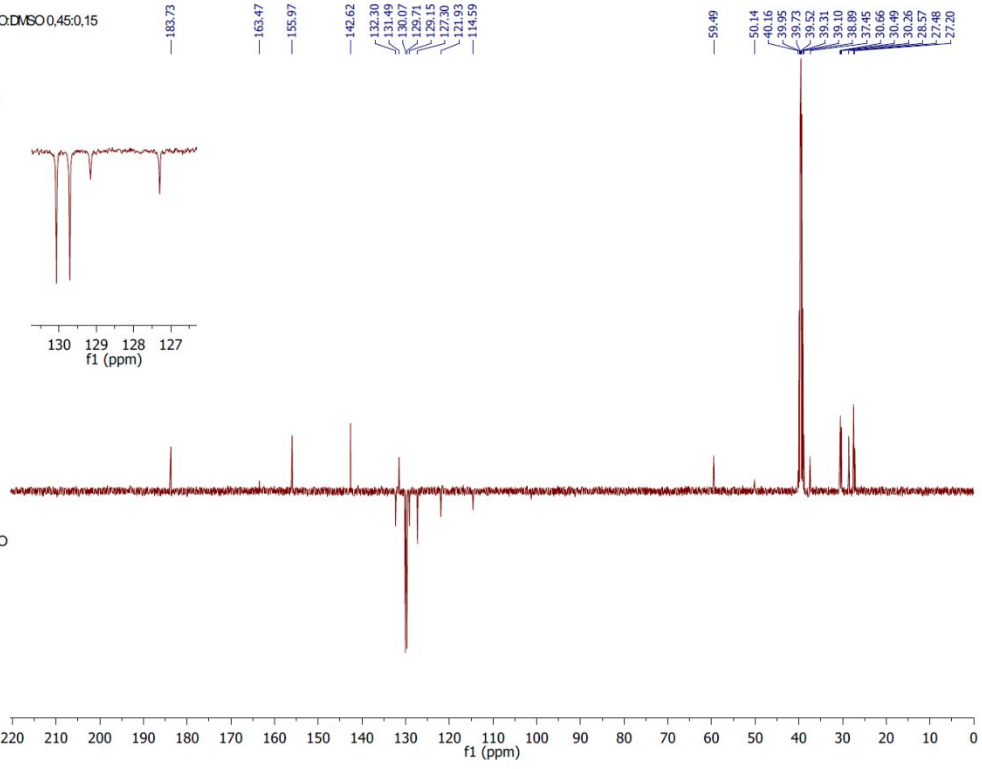

Chromatogram
ZLN055_P_Acid2 C:\LabSolutions\Data\2013\2013-wk36\ZLN055_P_Acid2.lcd

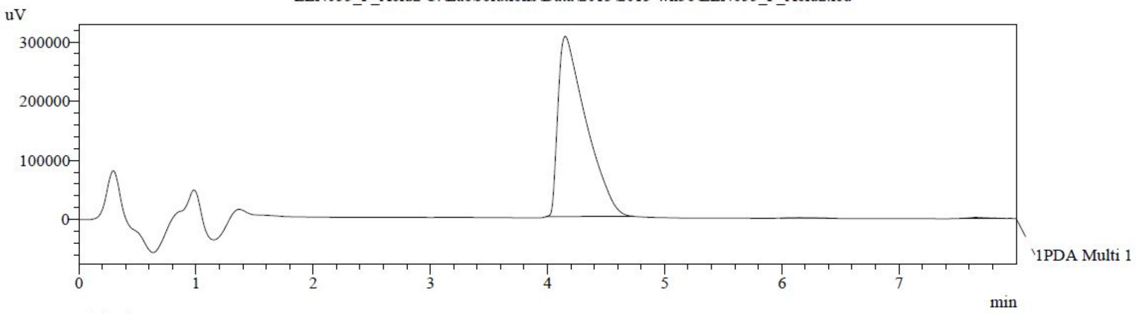

1 PDA Multi 1 / 230nm 4nm

PeakTable

PDA Ch1 230nm 4nm

Peak#	Name	Ret. Time	Area	Area %
1		4.147	5064031	99.533
2		6.135	12125	0.238
3		7.658	11657	0.229

Compound 27

Name: 1H - MAST070 after high vacuum
Date & Time: 2015-04-23T00:14:39
Nucleus: 1H
Solvent: DMSO
Number of Scans: 16
Spectrometer Frequency: 500.23 MHz
Temperature: 300 K
Pulse Sequence: zg30
Relaxation Time: 1 sec

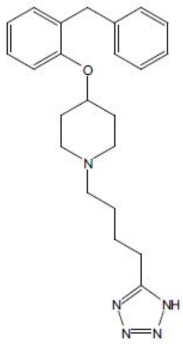

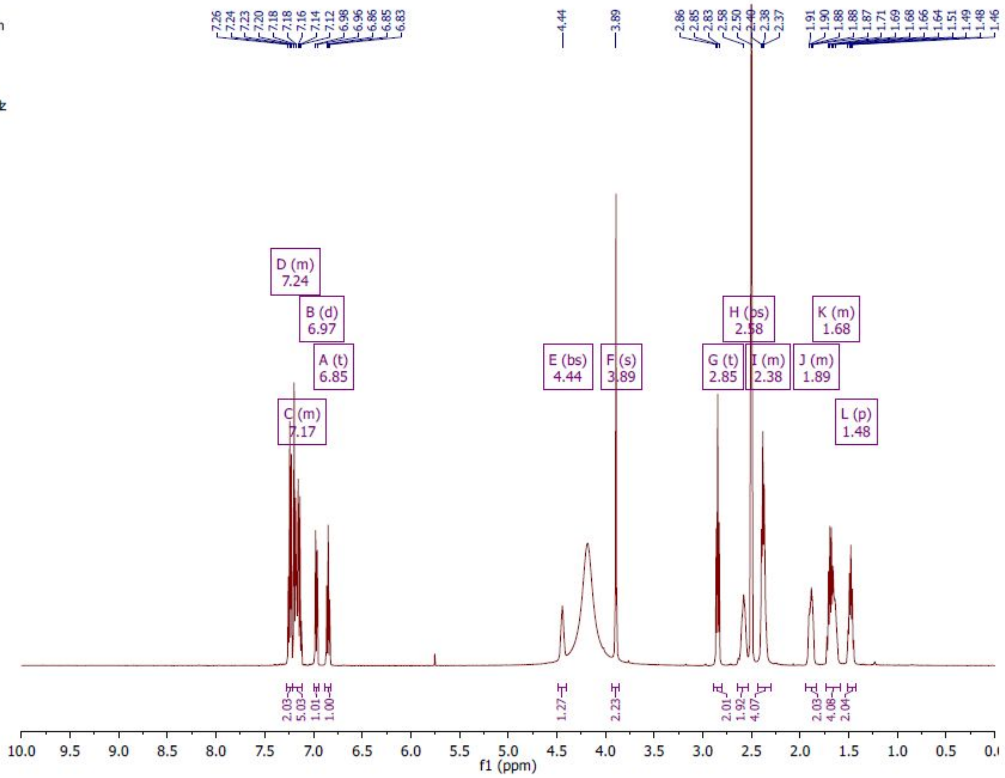

Name: 13CAPT - MAST070 after high vacuum
Date & Time: 2015-04-23T02:03:00
Nucleus: 13C
Solvent: DMSO
Number of Scans: 2048
Spectrometer Frequency: 125.8 MHz
Temperature: 300.1 K
Pulse Sequence: jmod
Relaxation Time: 2 sec

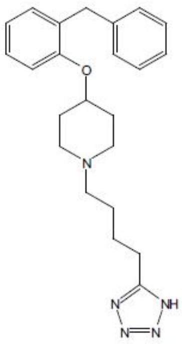

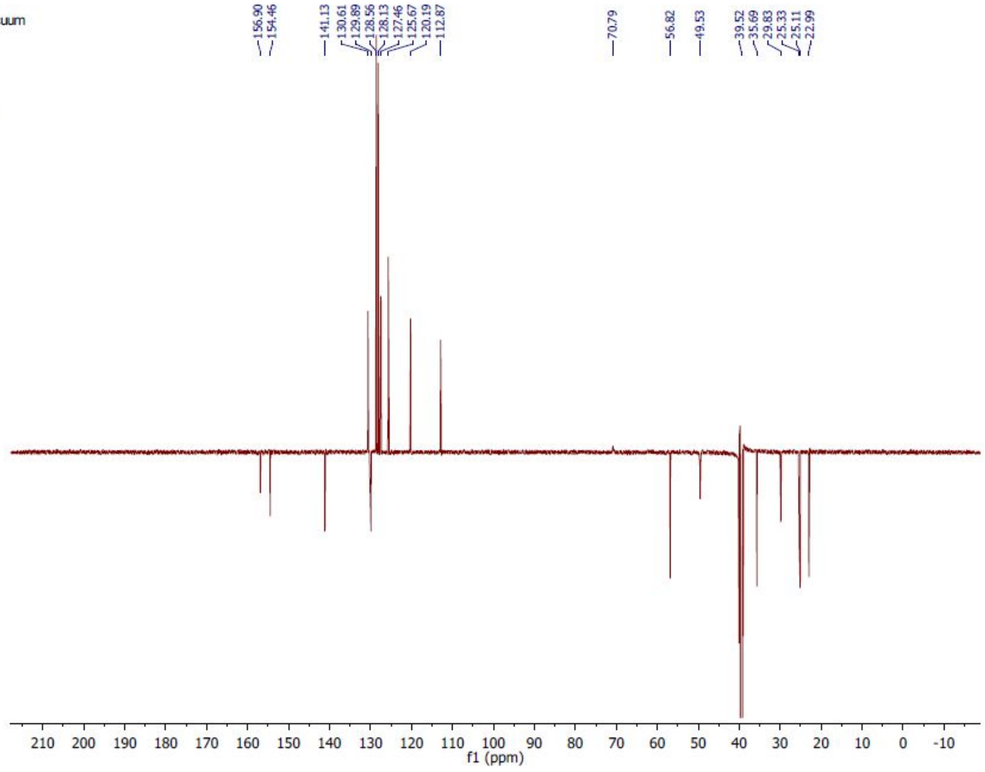

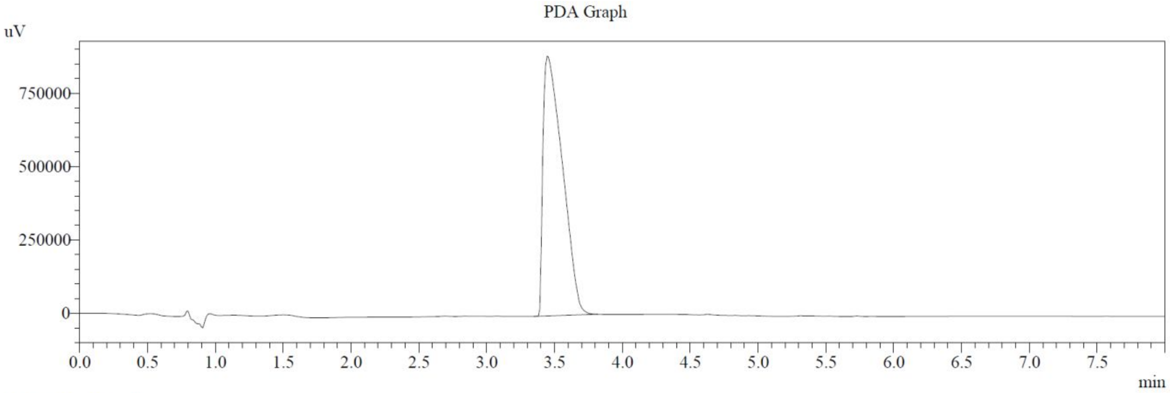

PDA Ch1 230nm 4nm

Peak#	Name	Ret. Time	Area	Area %
1		3.444	8835322	100.000
